# Anaerobiosis influences virulence properties of *Pseudomonas aeruginosa* cystic fibrosis isolates and the interaction with *Staphylococcus aureus*

**DOI:** 10.1038/s41598-019-42952-x

**Published:** 2019-05-01

**Authors:** Ross Pallett, Laura J. Leslie, Peter. A. Lambert, Ivana Milic, Andrew Devitt, Lindsay J. Marshall

**Affiliations:** 1 School of Life and Health Sciences, Aston University, Birmingham, UK; 2 Aston Institute of Materials Research, School of Engineering and Applied Science, Aston University, Birmingham, UK; 3 Present Address: Humane Society International, The Humane Society of the United States, 700 Professional Drive, Gaithersburg, MD 20879 USA

**Keywords:** Microbiology, Bacteriology

## Abstract

The airways of individuals with cystic fibrosis (CF) are abundantly colonised by *Staphylococcus aureus* and *Pseudomonas aeruginosa*. Co-infecting hypoxic regions of static mucus within CF airways, together with decreases in pulmonary function, mucus plugging and oxygen consumption by host neutrophils gives rise to regions of anoxia. This study determined the impact of anaerobiosis upon *S. aureus*-*P. aeruginosa* interactions in planktonic co-culture and mixed species biofilms *in vitro*. Whilst anoxia reduced the ability for *P. aeruginosa* CF isolates to dominate over *S. aureus*, this occurred in an isolate dependent manner. Investigations into the underlying mechanisms suggest that the anti-staphylococcal compound facilitating *P. aeruginosa* dominance under normoxia and anoxia is greater than 3 kDa in size and is heat-stable. Not all interspecies interactions studied were antagonistic, as *S. aureus* exoproducts were shown to restore and enhance *P. aeruginosa* motility under normoxia and anoxia in an isolate dependent manner. Collectively, this study suggests changes in oxygen availability within regions of the CF lung is likely to influence interspecies interactions and in turn, potentially influence disease progression.

## Introduction

Cystic fibrosis (CF) is the most common inherited genetic condition in the Caucasian population. An autosomal recessive disorder, CF arises due to mutations in the cystic fibrosis transmembrane conductance regulator (CFTR)^1^, an ion channel belonging to the ATP-Binding Cassette Transporter family of membrane proteins. Mutations within the CFTR lead to defective apical chloride ion transport, sodium hyperabsorption and a decrease in cell surface liquid^2,3^. This ultimately results in the production of highly viscous dehydrated mucus, affecting the normal physiology of numerous organs^3^. Impairment in mucociliary clearance within CF airways predisposes individuals to the development of several opportunistic respiratory infections, typically occurring in a highly-sequential, age-dependent manner^4^.

A commensal of the anterior nares^5^, the Gram-positive coccus *Staphylococcus aureus* (*S. aureus*) is often the first bacterium to be isolated from the sputum of children with CF and remains one of the most prevalent pathogens throughout the first decade of life^6,7^. CF airways are colonised by a complex polymicrobial community, with acquisition of the Gram-negative organism *Pseudomonas aeruginosa* (*P. aeruginosa*) typically occurring through environmental colonisation^8^, although patient-patient transmission has been shown to facilitate the spread of drug resistant epidemic strains^9,10^. As an individual progresses through adolescence and into early adulthood, *P. aeruginosa* replaces *S. aureus* as the dominant pathogen in CF airways^7,11^. There is temporal phenotypic diversity of *P. aeruginosa* during the course of CF airway infection^12,13^. Compared to the early infecting environmental strain, chronic infection with *P. aeruginosa* is associated with a loss of motility, loss of quorum sensing, the acquisition of the mucoid phenotype and increased resistance to antibiotics^14,15^.

The ability of *P. aeruginosa* to dominate has been attributed to an arsenal of extracellular virulence properties, several of which target *S. aureus*. The staphylolytic protease elastase A (LasA) degrades the peptidoglycan cell wall of *S. aureus*^16^, whilst the phenazine pyocyanin and secondary metabolites hydrogen cyanide and 2-heptyl-4-hydroxyquinoline N-oxide (HQNO) inhibit *S. aureus* oxidative respiration, consequently slowing its growth^17–19^. Despite this antagonism, *S. aureus* is detected in a third of CF adults who are culture positive for *P. aeruginosa*^7,20^, with two recent studies suggesting *S. aureus* and *P. aeruginosa* occupy identical regional niches of the CF lung^21,22^. Co-infection of CF airways with *S. aureus* and *P. aeruginosa* has been associated with decreased pulmonary function and an increased frequency of pulmonary exacerbations compared to mono-infection^20,23^.

Studies assessing polymicrobial CF airway infection to date, including *S. aureus*-*P. aeruginosa* interactions, have been conducted under normoxia (21% environmental oxygen), with vigorous culture aeration (200–250 rpm)^18,24–29^. However, increases in CF epithelia respiration, thick mucus plugging, decreased pulmonary function and oxygen consumption by polymicrobial communities and host neutrophils encourage the development of a static growth environment, with very low to absent levels of oxygen^30–34^. The presence of obligate anaerobes has also been shown to contribute to disease severity and inflammation in CF airways^35–37^.

Though *P. aeruginosa* is a facultative anaerobe that preferably uses aerobic respiration, its ability to undergo anaerobiosis is due to the presence of nitrate (NO_3_^−^), which acts as an end terminal electron acceptor^38,39^. Nitrate has been detected in both airway surface liquid (ASL) and in CF airway sputum^31,32,40^. A microarray study of *P. aeruginosa* obtained from CF sputum detected genes essential for *P. aeruginosa* denitrification^41^, whilst sera from individuals with CF have also been shown to contain antibodies to *P. aeruginosa* proteins involved in denitrification^42^.

Whilst anaerobiosis has previously been shown to influence the *P. aeruginosa* phenotype including growth, virulence and sensitivity to antibiotics^31,43–48^, its impact upon the interactions of *P. aeruginosa* with other CF pathogens has yet to be investigated. Initial experiments in this study sought to determine the impact of normoxia and anoxia upon the *in vitro* interactions between *S. aureus* and CF clinical isolates of *P. aeruginosa* in planktonic co-culture and mixed species biofilms, the two modes of *P. aeruginosa* growth known to occur in the CF lung. To provide insight into the mechanisms driving interspecies interactions under different oxygen conditions, the impact of anaerobiosis upon the production of several anti-staphylococcal exoproducts produced by *P. aeruginosa* were studied, which was further assessed using size fractionation and heat-treatment. Lastly, the impact of anaerobiosis and *S. aureus* exoproducts upon *P. aeruginosa* motility was determined. Data from this study suggest that oxygen availability exerts an isolate dependent effect upon *S. aureus*-*P. aeruginosa* interactions that influences the predominance of each organism within different regions of the CF lung.

## Results

### Effect of oxygen availability upon *S. aureus-P. aeruginosa* competition in planktonic co-culture

We sought to identify whether changes in oxygen availability influenced *S. aureus*-*P. aeruginosa* interactions in planktonic (free-swimming) culture. Reference strain *P. aeruginosa* PAO1 and *P. aeruginosa* CF isolates 5, 6 and 7 were selected for analysis following the growth inhibition results of the agar plate cross-streak assay (shown in Supplementary Fig. S1). PAO1 and *P. aeruginosa* CF clinical isolates 5 and 7 were able to inhibit *S. aureus* under normoxia, whilst CF isolate 6 had no effect upon the growth of *S. aureus*. Additionally, preliminary experiments demonstrated that these CF isolates differed with regards to protease production (assessed on skimmed milk agar) and pyocyanin (following bacterial culture upon Pseudomonas isolation agar). *S. aureus* ATCC 6538 was chosen due to it being an accessible and widely used laboratory reference strain. In addition to being known to produce biofilm, (important for the co-species biofilm studies)^49^, it has also been previously used to study *S. aureus*-*P. aeruginosa* interactions^50^. Furthermore, it has had its complete genome sequenced^51^, which may aid in determining the virulence properties it is likely to exhibit.

Density matched *S. aureus* and *P. aeruginosa* cultures were inoculated at an equal ratio, with planktonic growth competition being assessed over 24 h, comparing the colony counts of the bacteria grown in pure culture, to those grown in co-culture. To clearly compare the differences of growth in monoculture with co-culture, the competitive index (CI) and relative increase ratio (RIR) were calculated.

**Figure 1:**
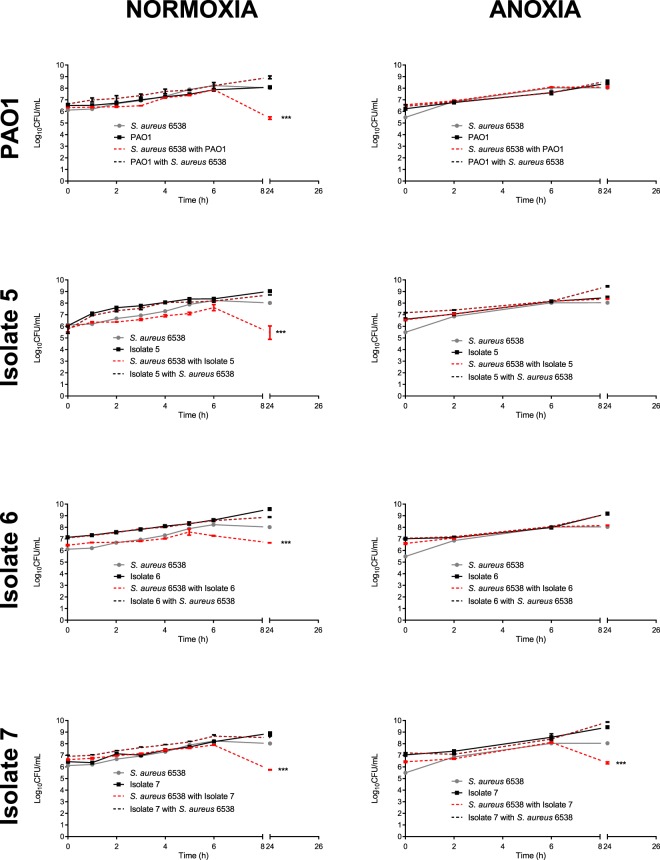
*S. aureus-P*. *aeruginosa* competition in planktonic co-culture under normoxia and anoxia. *S. aureus and P*. aeruginosa were grown statically at 37 °C for 24 h in either single or dual culture, under normoxia or anoxia. At regular intervals, aliquots were taken and plated onto selective agar. Each data point represents the mean ± S.E.M of three independent experiments (*N* = 3), each performed in triplicate. Statistical differences were determined using a two-way ANOVA with Bonferroni *post-hoc*, comparing the CI to the RIR at each time point ***P < 0.001.

As shown in Fig. 1 (left panel), all *P. aeruginosa* isolates tested were able to outcompete *S. aureus* under normoxia. PAO1 and CF isolates 5 and 7 caused an approximate 2.5 log reduction in viable *S. aureus* at 24 h (*P* < 0.001), whilst CF isolate 6 caused an approximate 1.5 log reduction in *S. aureus* at 24 h (*P* < 0.001). The CI of PAO1 versus *S. aureus* was significantly different from the RIR at 2, 3, 5, 6 and 24 h, whilst the CI for all CF isolates was significantly higher than the RIR at 6 and 24 h time points only (Supplementary Fig. S2). *S. aureus* killing was incomplete under normoxia for PAO1 and the CF isolates tested, with *S. aureus* still being detected at high bacterial counts at 24 h.

Similar experiments were conducted to determine the effects of anoxia upon co-culture competition (Fig. 1, right panel). *S. aureus* growth was unaffected by the presence of *P. aeruginosa* PAO1 and CF isolates 5 and 6 at all time points tested (see Supplementary Fig. S2), whilst CF isolate 7 retained its ability to dominate, causing an approximate 1 log reduction in the growth of *S. aureus* at 24 h compared to monoculture (*P* < 0.001).

### Oxygen availability and community composition in mixed species biofilms

As *S. aureus* and *P. aeruginosa* often co-exist within a polymicrobial biofilm within the CF lung, we determined the impact of oxygen availability upon the number of viable bacteria within single and mixed biofilms.

**Figure 2:**
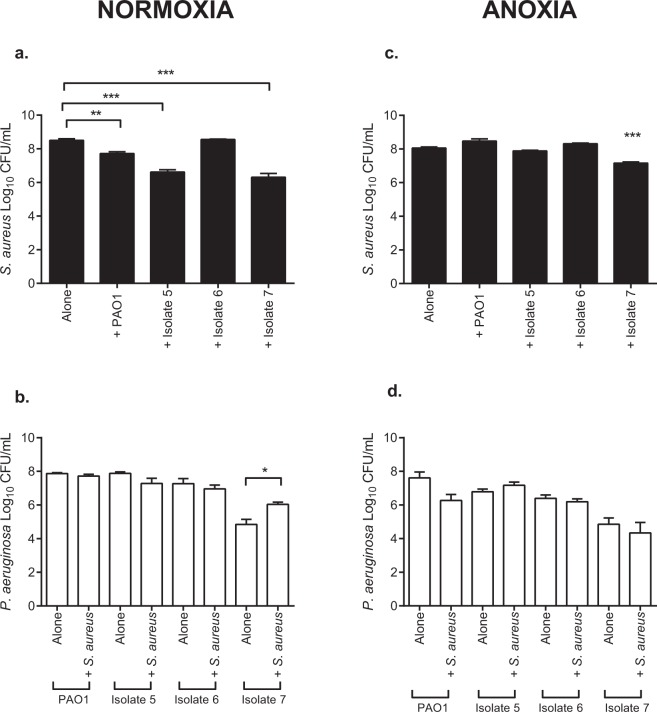
*S. aureus* and *P. aeruginosa* sessile viability in mono- and co-culture biofilms under normoxia and anoxia. Bacteria were grown in 96-well flat bottom plates either individually or in a 1:1 ratio for 24 h at 37 °C under static normoxia or anoxia. Biofilms were washed, detached, vortexed, serially diluted and plated onto selective agar for quantification. Bars represent the mean ± S.E.M of three individual experiments (*N* = 3), each performed in triplicate. Statistical differences were determined using one-way ANOVA with Tukey’s *post-hoc*. *P < 0.05, **P < 0.01, ***P < 0.001.

Under normoxia (Fig. 2a), *S. aureus* numbers were reduced in the presence of PAO1 (*P* < 0.01) and CF isolates 5 and 7 (*P* < 0.001), compared to *S. aureus* alone. Unlike in planktonic culture (Fig. 1), CF isolate 6 was unable to outcompete *S. aureus* in mixed species biofilm. Furthermore, *S. aureus* was unable to exert an antagonistic affect upon any of the *P. aeruginosa* isolates tested (Fig. 2b), with isolates of *P. aeruginosa* being recovered in numbers comparable to those isolated in single species biofilm. CF isolate 7 however, demonstrated a significant increase in CFU/mL in mixed species biofilm compared to single culture (*P* < 0.05).

Following growth under anoxia, PAO1 and CF isolates 5 and 6 were no longer able to outcompete *S. aureus* in mixed species biofilm (Fig. 2c) whilst CF isolate 7 retained its ability to reduce *S. aureus* viability (*P* < 0.001). All CF isolates tested were unaffected by the presence of *S. aureus* under anoxia with *P. aeruginosa* being enumerated at numbers similar to those in *P. aeruginosa* monoculture biofilms (Fig. 2d). The data for *S. aureus*-*P. aeruginosa* competition in planktonic co-culture and mixed species biofilms is summarised in a table in Supplementary Table S1.

### Impact of oxygen upon anti-staphylococcal exoproducts

It is evident that anoxia facilitates *S. aureus*-*P. aeruginosa* co-existence in an isolate dependent manner, in both planktonic co-culture and mixed species biofilms. To determine how oxygen availability permits bacterial co-existence and why *P. aeruginosa* CF isolate 7 was still able to reduce *S. aureus* viability, the production of several known anti-staphylococcal exoproducts in cell-free supernatants of *P. aeruginosa* were assessed following growth under normoxia and anoxia. This study focused upon the production of proteases, the lysis of *S. aureus*, the production of the respiratory inhibitor pyocyanin, the siderophore pyoverdine and biosurfactants.

**Figure 3:**
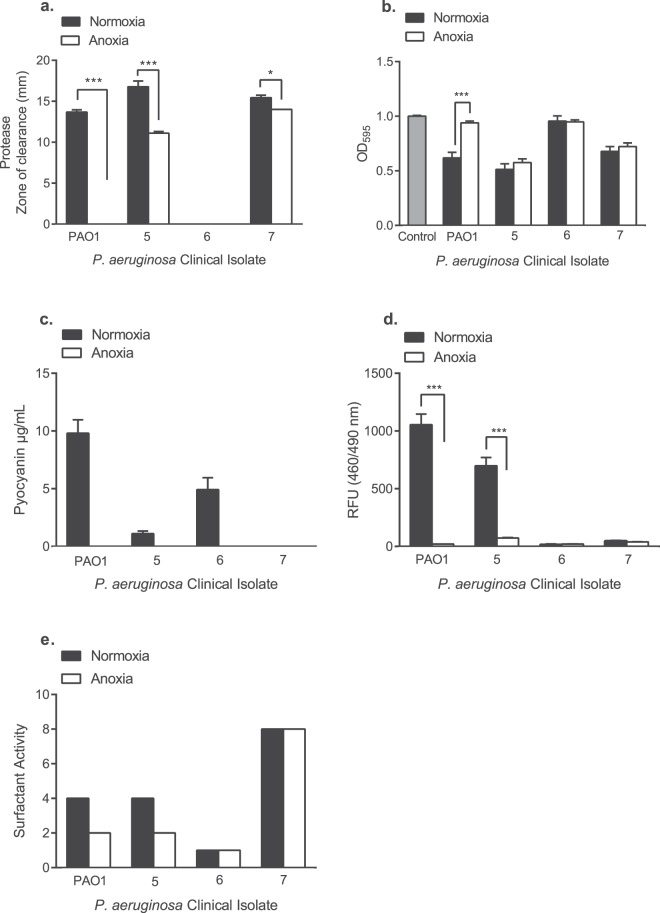
Impact of normoxia and anoxia upon the production of anti-staphylococcal exoproducts by *P. aeruginosa*. (**a**) Protease production was determined following the addition of *P. aeruginosa* cell-free supernatants to wells in milk agar, prior to the diameter of the zones of clearance (mm) being measured following 24 h incubation. (**b**) *P. aeruginosa* cell-free supernatants were added to heat-killed *S. aureus* and staphylolytic activity was determined by measuring changes in the OD_595_ after 60 min. The control consisted of heat-killed *S. aureus* with sterile LBN broth only. (**c**) Pyocyanin was extracted from *P. aeruginosa* cell-free supernatants using chloroform and 0.2 M hydrochloric acid. Spectrophotometric measurements were taken at OD_520_ and multiplied by extinction co-efficient of pyocyanin at this wavelength (17.072) to express pyocyanin in µg/mL. (**d**) Pyoverdine production was quantified by measuring the relative fluorescence units (RFU) of *P. aeruginosa* cell-free supernatants following excitation at 460 nm and emission at 490 nm. (**e**) Surfactant activity was measured using the established drop collapse assay, where cell-free supernatants were serially diluted (1:1) with deionised water containing 0.005% crystal violet to aid visualisation. Surfactant scores are equal to the reciprocal of the greatest dilution at which there was surfactant activity. Data shown are the mean ± S.E.M. of three independent experiments (*N* = 3), each performed in triplicate. In panels A, B and D, statistical differences were determined using one-way ANOVA with Tukey**’**s *post-hoc* test *P < 0.05, **P < 0.01 and ***P < 0.001.

As *P. aeruginosa* is known to secrete a number of proteases, skimmed milk agar has previously been used to study the total protease activity of *P. aeruginosa*^50,52^. The diameter of the zones of clearance were measured in millimetres (mm). As shown in Fig. 3a, PAO1 and CF isolates 5 and 7 produced detectable levels of protease under normoxia. Following growth under anoxia, CF isolates 5 and 7 retained their protease activity, whilst this was below the limit of detection for PAO1. CF isolate 6 failed to produce detectable proteases following growth under normoxia and anoxia.

*P. aeruginosa* PAO1 has previously been shown to lyse *S. aureus*^16^. Thus, the ability of *P. aeruginosa* CF isolates to lyse heat-killed *S. aureus* was also determined following growth under normoxia and anoxia, a method used previously^53–55^. As shown in Fig. 3b, CF isolate 6 was the only isolate unable to significantly lyse heat-killed *S. aureus* under normoxia (*P* > 0.05). Following growth under anoxia (Fig. 3b), PAO1 lost its ability to significantly lyse heat-killed *S. aureus* compared to normoxia (*P* < 0.001), whilst CF isolates 5 and 7 retained their significant staphylolytic activity under anoxia. CF isolate 6 did not lyse heat-killed *S. aureus* under anoxia.

**Figure 4:**
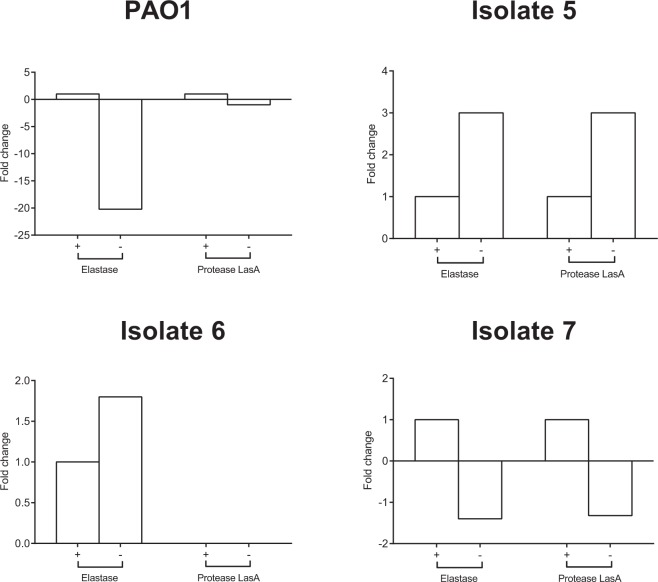
Detection of elastase and protease LasA in the *P. aeruginosa* cell-free secretome. Cell-free supernatants obtained from 24 h cultures of PAO1 and CF isolates 5, 6 and 7 grown under normoxia (+) and anoxia (−) were subject to protein precipitation, SDS-PAGE electrophoresis and MS analysis. Data represents *N* = 1 from five pooled samples. The abundance of elastase and protease LasA is expressed as fold-change compared to normoxia.

The complete cell-free secretomes of *P. aeruginosa* PAO1 and CF isolates 5, 6 and 7 are shown in Supplementary Tables S2 to S5. Expression levels of elastase and protease LasA following growth under normoxia and anoxia were studied by mass spectrometry (Fig. 4) to determine whether there was any correlation with the protease and staphylolytic activity shown in Fig. 3a,b. As shown in Fig. 4, whilst all of the isolates tested secreted detectable levels of elastase under both environmental conditions, CF isolate 6 was the only isolate which did not produce detectable levels of protease LasA under both normoxia and anoxia. Analysis of the PAO1 cell-free secretome demonstrated a 20.2-fold decrease in the relative abundance of elastase under anoxia, whilst exhibiting a minimal fold change in protease LasA. Conversely, CF isolates 5 and 7 exhibited a 3.0-fold increase and 1.4-fold decrease in elastase respectively, along with a 3.0-fold increase and 1.3-fold decrease in protease LasA. Once more, CF isolate 6 failed to exhibit staphylolytic activity under anaerobiosis (Fig. 4b), with no protease LasA being detected in its secretome (Fig. 4).

The production of the respiratory inhibitor pyocyanin was also determined. Under normoxia, PAO1 and CF isolates 5 and 6 produced varying degrees of pyocyanin following phenol-chloroform extraction, whilst this was below the limit of detection for CF isolate 7 (Fig. 3c). No pyocyanin was detected for any of the *P. aeruginosa* isolates following growth under anoxia.

As shown in Fig. 3d, *P. aeruginosa* PAO1 and CF isolate 5 produced pyoverdine under normoxia, with this being significantly reduced under anoxia (*P* < 0.001). CF isolates 6 and 7 produced minimal levels of pyoverdine, which was unaffected by anoxia. Lastly, PAO1 and all CF isolates tested exhibited varying degrees of biosurfactant activity, with this being the greatest for CF isolate 7 (Fig. 3e). Anoxia reduced the surfactant activity of PAO1 and CF isolate 5, but not for CF isolates 6 and 7. The data shown in Fig. 3 demonstrates that each *P. aeruginosa* CF isolate produces a varying array of anti-staphylococcal exoproducts under normoxia, with their production being further influenced by anoxia.

Using these data, we sought to greater determine the nature of the *P. aeruginosa* anti-staphylococcal compound which mediates *P. aeruginosa* dominance under both normoxia and anoxia. Cell-free supernatants from *P. aeruginosa* cultures were passed through 3 kDa molecular weight cut-off filters, in an effort to determine the size of the anti-staphylococcal compound. Whilst the 20 kDa protease LasA would be retained within the >3 kDa fraction, smaller virulence factors such as pyocyanin, rhamnolipids and pyoverdine would pass into the <3 kDa fraction. Heat-treatment was used to determine whether the anti-staphylococcal compound was proteinaceous in nature, as boiling would be expected to abolish its anti-staphylococcal activity. Cell-free culture supernatants were subsequently added to log-phase cultures of *S. aureus* to assess their ability to inhibit *S. aureus* planktonic growth over 14 h.

**Figure 5:**
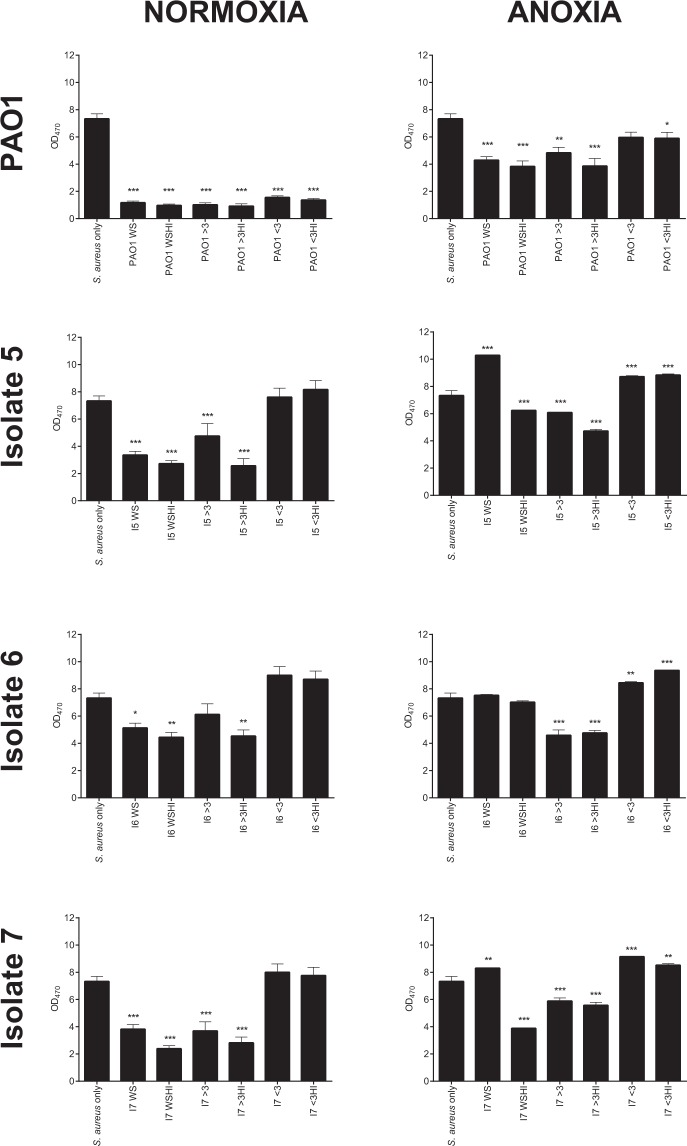
Heat-treatment and size fractionation of *P. aeruginosa* cell-free supernatants upon the growth of *S. aureus*. Cell-free supernatants from *P. aeruginosa* grown under normoxia or anoxia were added to standardised *S. aureus* directly or were first subjected to 3 kDa size fractionation, with select fractions then being subjected to boiling. Plates were incubated at 37 °C for 14 h and the OD_470_ of *S. aureus* cultures read at hourly intervals. Plots represent the mean OD at 14 h ± S.D. of two individual experiments (*N* = 2), each performed in duplicate. WS: Whole supernatant, WSHI: Whole supernatant heat-inactivated, >3: greater than 3 kDa, >3HI: greater than 3 kDa heat-inactivated, <3: less than 3 kDa, <3HI: less than 3 kDa heat-inactivated. Statistical differences were determined using one-way ANOVA with Dunnett**’**s *post-hoc* (versus *S. aureus* only control). *P < 0.05, **P < 0.01 ***P < 0.001.

As shown in Fig. 5, under normoxia, *P. aeruginosa* PAO1 whole supernatant, >3 kDa and <3 kDa fractions were able to significantly reduce *S. aureus* growth compared to *S. aureus* alone (*P* < 0.001). This anti-staphylococcal activity was not lost following heat-treatment of the fractions. Interestingly, *S. aureus* antagonism across all the CF clinical isolates tested under normoxia were restricted to the whole supernatant and >3 kDa fractions only, which was unaffected by heat-treatment.

Under anoxia (Fig. 5) significant anti-staphylococcal activity was detected in all but the untreated <3 kDa fraction of PAO1. Once again, the anti-staphylococcal activity of the fractions was unaffected by heat-treatment. The heat-treated whole culture supernatant and both the untreated and heat-treated >3 kDa fractions obtained from CF isolate 5 significantly reduced *S. aureus* growth. For CF isolate 6, only the >3 kDa fractions significantly reduced *S. aureus* growth, where once again, heat-treatment failed to abolish this activity. As seen with CF isolate 5, the heat-treated whole culture supernatant and the untreated and heat-treated >3 kDa fractions obtained from CF isolate 7 were the most inhibitory towards *S. aureus* growth. Collectively, this suggests that the major anti-staphylococcal exoproduct produced by *P. aeruginosa* is heat-stable and >3 kDa in size.

Proteomic analysis of the cell-free secretomes of PAO1 and CF isolates 5, 6 and 7 (Supplementary Tables S2 to S5) did not identify any obvious lead candidate for the active factor which is likely to mediate *P. aeruginosa* dominance under normoxia and anoxia. Whilst the mass spectrometry experiments performed were used to generate a proteomic dataset only, it is possible that the extracellular anti-staphylococcal factor is not proteinaceous in nature. Thus, further work is required in order to identify the active anti-staphylococcal factor.

### Impact of *S. aureus* exoproducts and anoxia upon *P. aeruginosa* motility

Lastly, as *S. aureus* infection precedes *P. aeruginosa*, we sought to determine the influence of *S. aureus* exoproducts upon *P. aeruginosa* swimming and swarming motilities. Both free and surface attached forms of motility are likely to play important roles in airway colonisation and disease progression. The impact of anoxia upon *P. aeruginosa* motility in the presence and absence of *S. aureus* exoproducts was also determined.

**Figure 6:**
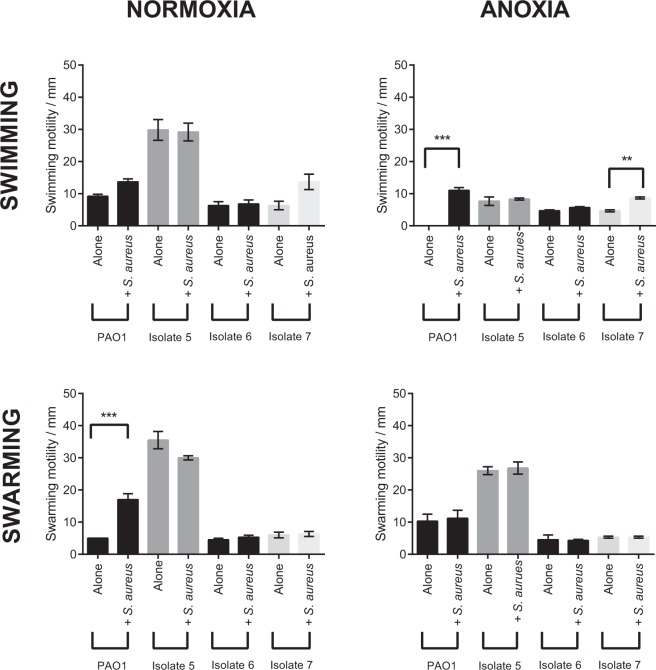
Effect of *S. aureus* culture supernatant and oxygen upon *P. aeruginosa* motility. Swimming and swarming motilities of *P. aeruginosa* isolates were assessed following an overnight incubation on swimming and swarming agar plates. To test the effects of *S. aureus* culture supernatant upon these two forms of motility, a 1:100 dilution of the culture supernatant was added to plates before the agar set. Data are presented as mean ± S.E.M from three independent experiments each performed in duplicate. Statistical differences were determined using one-way ANOVA with Tukey**’**s *post-hoc*. **P < 0.01 ***P < 0.001.

PAO1 and the CF isolates tested exhibited varying degrees of swimming motility under normoxia (Fig. 6). CF isolate 5 alone exhibited significantly greater swimming motility under normoxia compared to PAO1, CF isolate 6 and CF isolate 7 alone (*P* < 0.001). *P. aeruginosa* motility was unaffected by the inclusion of *S. aureus* exoproducts. Under anoxia, CF isolate 5 alone exhibited the greatest swimming motility compared to PAO1 alone (*P* < 0.01) and CF isolates 6 and 7 alone (*P* < 0.05). Compared to normoxia, anoxia reduced the swimming motility of PAO1 alone (*P* < 0.01), CF isolate 5 alone (*P* < 0.001) and CF isolate 5 in the presence of *S. aureus* (*P* < 0.001). PAO1 alone lost its swimming motility under anoxia whilst the addition of *S. aureus* cell-free supernatant significantly restored the swimming motility of PAO1 (*P* < 0.001). *S. aureus* cell-free supernatant also enhanced the swimming motility of CF isolate 7 compared to CF isolate 7 alone (*P* < 0.01).

Under normoxia, CF isolate 5 alone exhibited the greatest swarming motility compared to PAO1 and CF isolates 6 and 7 alone (*P* < 0.001) (Fig. 6). The addition of *S. aureus* exoproducts only enhanced the swarming motility of PAO1 (*P* < 0.001). Under anoxia, CF isolate 5 exhibited the greatest swarming motility compared to PAO1, including CF isolates 6 and 7 alone (*P* < 0.001). *S. aureus* exoproducts did not modulate *P. aeruginosa* swarming motility under anoxia. Only CF isolate 5 alone exhibited a significant reduction in swarming motility under anoxia compared to normoxia (*P* < 0.01).

**Figure 7:**
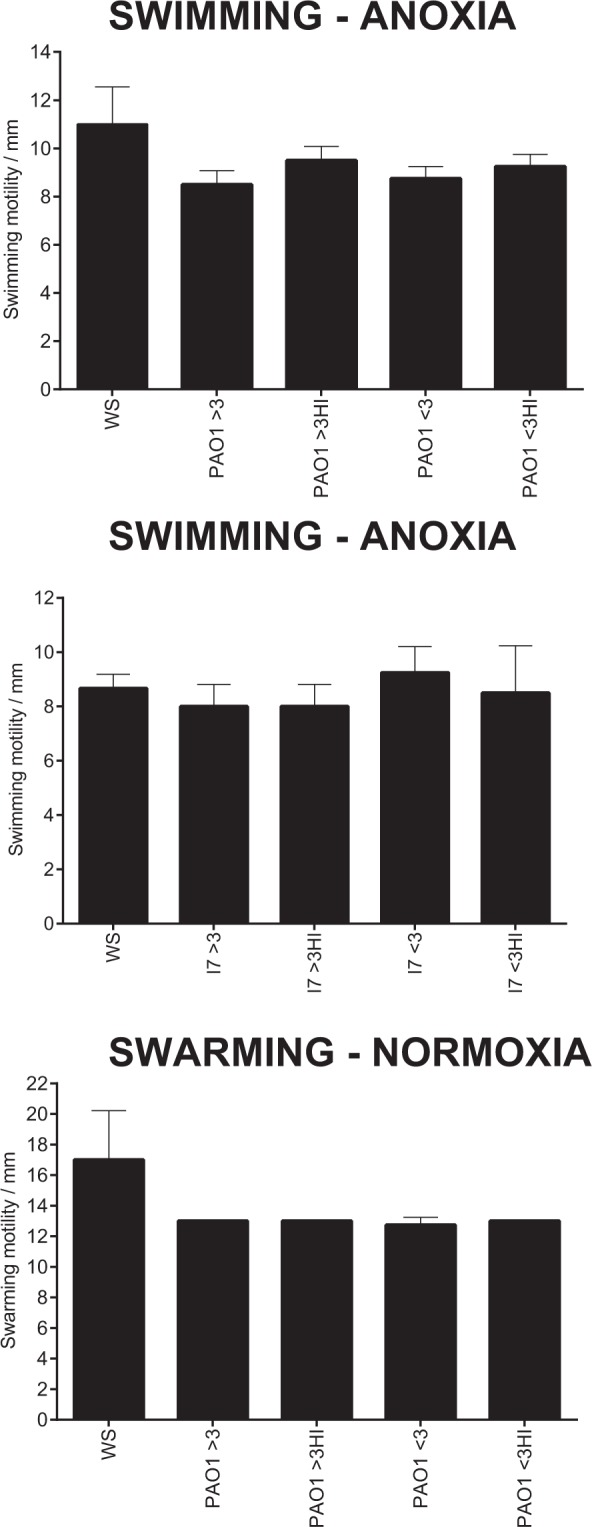
Heat-treatment and size fractionation of *S. aureus* cell-free supernatants upon *P. aeruginosa* swimming and swarming motility. Swimming and swarming motilities of the select *P. aeruginosa* isolates were assessed following an overnight incubation on swimming and swarming agar plates. *S. aureus* only motility values were taken from Fig. 6. WS: whole supernatant fractions of *S. aureus* culture supernatant were taken from Fig. 6. Plates were supplemented with a 1:100 dilution of *S. aureus* culture supernatant fractions (>3 or <3 kDa), with select fractions also being subject to heat-treatment. >3: greater than 3 kDa, >3HI: greater than 3 kDa heat-inactivated, <3:less than 3 kDa, <3HI: less than 3 kDa heat-inactivated. Data are presented as mean ± S.D from two independent experiments (*N* = 2) each performed in duplicate. Statistical differences were determined using one-way ANOVA with Dunnett’s *post-hoc* (versus WS: whole supernatant only).

As *S. aureus* exoproducts were shown to modulate the swimming motility of PAO1 and CF isolate 7 under anoxia, including the swarming motility of PAO1 under normoxia (Fig. 6), *S. aureus* cell-free supernatants were subjected to size fractionation and heat-treatment to determine the size and nature of the effector compound(s). As shown in Fig. 7, all fractions regardless of size and heat-treatment were able to modulate *P. aeruginosa* motility to the same extent as the whole culture supernatants (Fig. 6).

## Discussion

It is now appreciated that mucus plugging, decreases in pulmonary function and the consumption of oxygen by polymicrobial communities and host neutrophils create microenvironments in CF airways which are devoid of oxygen^31,56^. Whilst previous studies have demonstrated anaerobiosis attenuates the growth and virulence of *P. aeruginosa* laboratory strains and clinical isolates^43,44^, this study aimed to provide novel insights into the impact of anaerobiosis upon the interactions of *S. aureus* and CF clinical isolates of *P. aeruginosa* in planktonic co-culture and mixed species biofilms, including the possible mechanisms governing interspecies interactions under both environmental conditions.

Our competition data showed oxygen availability exerts a major role in *S. aureus* and *P. aeruginosa* competition in both planktonic co-culture and mixed species biofilms. Under normoxia (Fig. 1), PAO1 and all CF isolates 5, 6 and 7 were all able to outcompete *S. aureus* in planktonic co-culture at 24 h, without their own growth being adversely affected. This finding is supported by other *in vitro* studies conducted under normoxia where *P. aeruginosa* CF isolates were shown to reduce the viability of *S. aureus* in planktonic culture^25,27,57^. In all planktonic 24 h co-culture experiments, *P. aeruginosa* was unable to completely kill *S. aureus*.

PAO1 and CF isolates 5 and 7 retained their ability to antagonise *S. aureus* growth in mixed species biofilms under normoxia, whilst CF isolate 6 did not (Fig. 2). The inability of CF isolate 6 to outcompete *S. aureus* in mixed species biofilms as in planktonic co-culture (Figs 1 and 2) is potentially explained by previous studies which demonstrate differences in bacterial virulence following growth in planktonic and biofilm culture^58–60^.

The introduction of anoxia caused PAO1 and CF isolates 5 and 6 to lose their ability to outcompete *S. aureus* in both planktonic co-culture and mixed species biofilms (Figs 1 and 2), where *S. aureus* was enumerated in densities seen in monoculture. These results support those of a previous study which demonstrated anaerobiosis reduced the ability of *P. aeruginosa* PAO1 to induce zones of clearance upon confluent bacterial lawns of *Staphylococcus epidermidis*^61^. Furthermore, the culture of *P. aeruginosa* strains under hypoxia (1% oxygen) has previously been shown to attenuate *P. aeruginosa* virulence in an *in vivo* model of infection compared to normoxia through decreased expression of several virulence factors^62^. From these data, it is possible that anoxia is one of several mechanisms facilitating isolate specific *S. aureus-P. aeruginosa* co-existence in the CF lung.

Interestingly, CF isolate 7 was the only *P. aeruginosa* isolate able to reduce *S. aureus* viability under anoxia, in both planktonic co-culture and mixed species biofilm (Figs 1 and 2). To greater understand this phenomenon, the production of several anti-staphylococcal exoproducts was assessed across isolates, following growth under normoxia and anoxia. The use of molecular weight cut off filters and heat-treatment of the cell-free fractions served to determine the size and nature of the anti-staphylococcal compound likely to be mediate *P. aeruginosa* dominance under normoxia and anoxia.

PAO1 has previously been shown to produce LasA, responsible for cleaving the peptidoglycan cell wall of *S. aureus*^24,63^. Unlike CF isolate 6, PAO1 and CF isolates 5 and 7 were able to lyse heat-killed *S. aureus* under normoxia (Fig. 3b). PAO1 lost its staphylolytic ability under anoxia however (Fig. 3b), a finding supported by reports of a decrease in the transcription of LasA for PAO1 under anoxia^64^ and a reduction in PAO1 elastase B (LasB) production under anoxia^44^. Perhaps the LasA produced by PAO1 following growth under anoxia is below a threshold to exert a considerable affect upon *S. aureus* viability compared to that produced by CF isolates 5 and 7, although further work is required to determine this. *S. aureus* has also previously been shown to exhibit an increase in cell wall thickness under anaerobiosis^65^ and whilst this was not investigated in this study, this mechanism may further reduce *S. aureus* susceptibility to *P. aeruginosa* LasA under anoxia.

Whilst LasA may be advantageous to *P. aeruginosa* under growth in polymicrobial communities with *S. aureus*, collectively, LasA mediated lysis of *S. aureus* alone does not appear to be essential for facilitating *P. aeruginosa* dominance under normoxia. All CF isolates were able to outcompete *S. aureus* in planktonic co-culture (Fig. 1), yet CF clinical isolate 6 failed to exhibit detectable staphylolytic activity (Fig. 3b) or secrete detectable protease LasA as determined by mass spectrometry (Fig. 4). LasA also does not appear to be essential for modulating *P. aeruginosa* dominance under anoxia, as CF isolates 5 and 7 both retained their staphylolytic activity under anoxia (Fig. 3b). Only CF isolate 7 was able to outcompete in planktonic co-culture (Fig. 1) and mixed species biofilm (Fig. 2). Furthermore, whilst whole culture supernatants and >3 kDa fractions from PAO1 and the CF isolates were able to antagonise *S. aureus* growth, heat-treatment failed to abolish their inhibitory activity (Fig. 5). As staphylolysis is known to be mediated by LasA, boiling such fractions would be expected to denature this 20 kDa protease.

The role of LasA in governing interspecies interactions may also be complicated by previous findings which demonstrate how the growth of *S. aureus* in the presence of *P. aeruginosa* LasA and staphylolysin produced by *Staphylococcus staphylolyticus*, selects for *S. aureus* colonies which lack a cell wall^66,67^. Whilst the emergence of such colonies in CF airways requires further study, selection of such a phenotype may serve to facilitate *S. aureus* persistence under both normoxia and anoxia in the presence of *P. aeruginosa*.

As previously mentioned, pyocyanin inhibits *S. aureus* oxidative respiration, causing a reduction in growth. PAO1 and CF isolates 5 and 6 produced detectable levels of pyocyanin under normoxia following phenol-chloroform extraction, whilst this was below the limit of detection for CF isolate 7 (Fig. 3c). Despite this, CF isolate 7 was still able to dominate *S. aureus* under normoxia in planktonic co-culture (Fig. 1) and mixed species biofilms (Fig. 2). Furthermore, pyocyanin was below the limit of detection for all culture supernatants cultured under anoxia following phenol-chloroform extraction (Fig. 3c), yet CF isolate 7 was still able to dominate. Whilst the inability to detect pyocyanin under anoxia may be due to the redox nature of the phenazine^68^, a previous study demonstrated that hypoxia significantly reduced pyocyanin production by *P. aeruginosa*^69^.

Proteins involved in phenazine biosynthesis were only detected in the secretomes of *P. aeruginosa* PAO1 and *P. aeruginosa* CF isolate 5. As shown in Supplementary Table S2, *P. aeruginosa* PAO1 exhibited a 10.4-fold decrease in phenazine biosynthesis protein PhzB 1 and a 12.3-fold decrease in phenazine biosynthesis protein PhzB 2 respectively under anoxia. Additionally, there was a 21.0-fold decrease in Phenazine biosynthesis protein PhzB 1 for *P. aeruginosa* CF isolate 5 under anoxia (Supplementary Table S3). These data support the phenotypic data relating to pyocyanin production shown in Fig. 3c.

Size exclusion and heat-treatment of the *P. aeruginosa* cell-free supernatants also demonstrated that *S. aureus* antagonism was restricted to the >3 kDa fraction and was not abolished following heat-treatment (Fig. 5). However, it is expected that pyocyanin would be present in the <3 kDa fraction and that heat-treatment would abolish its activity.

Pyoverdine production by *P. aeruginosa* is known to reduce *S. aureus* growth, and enhance *P. aeruginosa* virulence^27,29,70^. As shown in Fig. 3d, PAO1 and CF isolate 5 produced large amounts of pyoverdine. Furthermore, anoxia was shown to significantly reduce pyoverdine production by PAO1 and CF isolate 5 compared to normoxia, a finding also reported following the growth of *P. aeruginosa* under hypoxia^69^. Despite CF isolates 6 and 7 producing minimal amounts of pyoverdine under normoxia (Fig. 3d), all *P. aeruginosa* CF isolates were able to outcompete *S. aureus* in planktonic co-culture (Fig. 1). Additionally, CF isolate 7 was able to outcompete *S. aureus* under anoxia despite producing minimal amounts of pyoverdine. As the antimicrobial activity of *P. aeruginosa* cell-free supernatants was restricted to the >3 kDa fractions (Fig. 5), it is unlikely that siderophore production in isolation drives *S. aureus* antagonism. However, we recognise the importance of pyoverdine in governing interspecies interactions may be underestimated in this study as iron-depleted growth media was not used to culture *P. aeruginosa*.

The surfactant activity of *P. aeruginosa* rhamnolipids has been previously shown to target *S. aureus* and reduce its viability^71–73^. Our data demonstrated that PAO1 and the CF *P. aeruginosa* isolates tested harboured biosurfactant activity under normoxia, with this being the greatest for CF isolate 7 (Fig. 3e). Whilst CF isolate 7 retained its high surfactant score under anoxia, size exclusion studies demonstrated that the anti-staphylococcal activity for all isolates was restricted to the >3 kDa fraction under both normoxia and anoxia (Fig. 5) and yet it is expected that the rhamnolipids would be retained within the <3 kDa fractions.

Collectively, it is reasonable to suggest from the results of this study that the anti-staphylococcal compound which facilitates *P. aeruginosa* dominance under both normoxia and anoxia is heat-stable, >3 kDa in size and is likely not one of the anti-staphylococcal factors studied. An interesting observation in Fig. 5 is that the heat-treated whole supernatant and >3 kDa fractions for most *P. aeruginosa* CF isolates under normoxia and anoxia appeared to more greatly reduce *S. aureus* growth compared to those which had not been heat treated. It is reasonable that heating may either increase the activity of the anti-staphylococcal factor, or alternatively lead to the loss of a factor within the supernatant which may otherwise protect *S. aureus* directly or indirectly. We also recognise that a limitation of this study is that the absolute concentration of the *P. aeruginosa* exoproducts in cell-free culture supernatants was not determined and it may be that some of the virulence factors were at concentrations too low to exert an antagonistic effect upon *S. aureus*.

*P. aeruginosa* is known to employ several other virulence properties which were not characterised in this study and may contribute to the differences seen in bacterial competition. This includes the respiratory inhibitor hydrogen cyanide^74^. However, its role in governing *S. aureus*-*P. aeruginosa* interactions is likely to be minimal in this study as it would be found in the <3 kDa fraction, and yet these fractions did not exert considerable anti-staphylococcal activity upon exposure to planktonic *S. aureus* under normoxia (Fig. 5). Furthermore, previous research has demonstrated that hydrogen cyanide is rapidly inactivated under strict anoxia^75^ and thus its ability to reduce *S. aureus* viability under anoxia is considered minimal.

*P. aeruginosa* also produces the respiratory inhibitor HQNO, which is known to reduce *S. aureus* viability^19,27,76^. Its production is regulated by *Pseudomonas* quinolone signal (PQS)^77^. PQS production is known to be suppressed under anoxia^61,78^. As shown in Supplementary Table S5, PQS proteins were only detected in the secretome of *P. aeruginosa* CF isolate 7. Whilst there was a 9.4-fold decrease in PqsB under anoxia, there was a 4.5-fold increase in PqsC under anoxia. Thus, further work is required to determine the role of HQNO in governing *S. aureus-P. aeruginosa* interspecies interactions.

It is also reasonable to suggest that the ability of *P. aeruginosa* to dominate under both normoxia and anoxia is not due to the secretion of a single anti-staphylococcal factor, but is due to the production of many^20,27,79–81^. A previous study has reported that deletion of a single virulence property has been shown to reduce *P. aeruginosa* antagonism towards *S. aureus*^82^. The production of alginate by *P. aeruginosa* has been reported to facilitate *S. aureus*-*P. aeruginosa* co-existence within the CF lung, with the overproduction of alginate inhibiting the synthesis secretion of pyoverdine, HQNO and rhamnolipids^82^. However, as none of the *P. aeruginosa* CF isolates used in this study were mucoid, alginate is unlikely to play a role in facilitating interspecies co-existence.

Finally, we also show how not all *S. aureus-P. aeruginosa* interactions studied are antagonistic. Whilst *S. aureus* has previously been shown to enhance *P. aeruginosa* antibiotic resistance^83,84^, we have demonstrated that *S. aureus* exoproducts were able to restore and enhance *P. aeruginosa* swimming and swarming motility in an isolate dependent manner under normoxia and anoxia (Fig. 6).

Previous research has demonstrated that the intracellular accumulation of the signalling molecule bis-(3′-5′)-cyclic dimeric GMP (c-di-GMP) favours *P. aeruginosa* biofilm formation^85^ through the production of exopolysaccharide, whilst repressing flagella motility. Conversely, intracellular decreases in c-di-GMP inhibits exopolysaccharide production and promotes flagella mediated motility^86,87^. These effects of intracellular c-di-GMP are mediated through its ability to bind and modulate the transcriptional regulator FleQ^88,89^. It is possible that one or more *S. aureus* exoproducts within the cell-free supernatant may lower the intracellular concentration c-di-GMP in *P. aeruginosa*, promoting motility (Fig. 6). Additional work is required as the modulating ability of *S. aureus* culture supernatant upon *P. aeruginosa* motility was not seen in all isolates and could not be restricted to a single fraction (Fig. 7).

Twitching motility in the presence and absence of *S. aureus* exoproducts was also investigated but in our hands no twitching motility was detected in any strains. As anoxia was also shown to influence *P. aeruginosa* motility, the presence of *S. aureus* and changes in oxygen availability are both likely to influence *P. aeruginosa* colonisation and dissemination into microenvironments within the CF lung.

## Conclusion

This study provides novel findings concerning the impact of anoxia upon interactions between *S. aureus* and CF isolates of *P. aeruginosa* in mixed planktonic culture and mixed species biofilms. We hypothesise from our data that anoxia is one of several potential mechanisms facilitating *S. aureus*-*P. aeruginosa* co-existence in the CF lung, with co-infection having been associated with poor clinical outcomes and a worsening of pulmonary function in CF^20,90^. However, not all *P. aeruginosa* CF isolates lose their ability to antagonise *S. aureus* growth under anoxia. Further work is required to determine the nature of the major anti-staphylococcal compound which facilitates *P. aeruginosa* dominance under both normoxia and anoxia, although this study provides evidence that the factor is likely to be heat-stable and >3 kDa in size. The ability of select *P. aeruginosa* CF isolates to retain their virulence and motility under anoxia is likely to influence disease progression differently across the CF population. The ability of *S. aureus* exoproducts to further modulate *P. aeruginosa* motility in an isolate dependent manner, reinforces the complex interspecies interactions known occur within the CF lung.

As co-isolated strains of *S. aureus* and *P. aeruginosa* are shown to be less antagonistic than those from mono-infected patients^82^, a longitudinal study of select *S. aureus* and *P. aeruginosa* co-isolates could be performed. This would allow interspecies interactions and the pathoadaptive mechanisms that occur overtime in both *S. aureus* and *P. aeruginosa* to be studied in greater detail and allow comparisons to be made under different environmental conditions. It is hoped that this study encourages interspecies interactions of other key CF pathogens to be determined under anoxia, an environmental condition shown to be important in modulating bacterial virulence and interspecies interactions.

## Materials and Methods

### Bacterial strains and growth conditions

All bacterial strains used in this study are listed in Table 1, with Table 2 describing their colony morphotype following growth upon LB agar. Eight clinical isolates of *P. aeruginosa* were cultured from paediatric CF sputum samples from Birmingham Children’s Hospital, UK between 1990–1999. All bacterial strains were stored at −80 °C in 50% (v/v) glycerol/water. Bacteria were streaked from frozen stocks to Luria Bertani (LB) agar plates (Fisher Scientific, UK) and incubated at 37 °C for 48 h. Strains were identified using selective agar, Gram staining and biochemical testing.

**Table 1:** *S. aureus* and *P. aeruginosa* strains used in this study.

Strain	Species	Source	Reference
6538	*S. aureus*	American Type Culture Collection (ATCC)	^99^
PAO1	*P. aeruginosa*	Wound exudate, Melbourne, Australia	^100^
Isolate 1	*P. aeruginosa*	CF Sputum from Birmingham Children’s Hospital, Birmingham, UK	This study
Isolate 2			
Isolate 3			
Isolate 4			
Isolate 5			
Isolate 6			
Isolate 7			
Isolate 8

**Table 2:** Colony morphology of *P. aeruginosa* laboratory strain PAO1 and clinical CF isolates upon LB agar plates.

Isolate	Pigmentation				Colony Size		Mucoidy status		Form		Optical Property	Autolysis	Surface Texture	
	Brown	Green	White	Opaque	Small	Large	Mucoid	Non-mucoid	Circular	Irregular	Opaque		Rough	Smooth
PAO1		+				+		+	+		+	−		+
1			+	+		+		+	+		+	−		+
2				+		+		+	+		+	−		+
3			+			+		+	+		+	−		+
4	+	+				+	+		+		+	−		+
5		+				+		+	+		+	−		+
6		+				+		+	+		+	−		+
7			+			+		+	+		+	−		+
8	+					+		+	+		+	−		+

The production of four colony pigmentations were studied. Colonies were positive for mucoidy status if they exhibited a slimy appearance. Colony form assessed the basic shape of the colonies. Autolysis assessed whether the bacteria grew as colonies that were lysed in their centres, whilst margin determined the edge of the colonies.

All assays were carried out using standardised bacterial inoculum. Single well isolated colonies of *S. aureus* or *P. aeruginosa* were routinely inoculated into 10 mL of sterile LBN broth (LB broth supplemented with 1% (w/v) potassium nitrate) (Fisher Scientific, UK) and grown at 37 °C under static conditions of normoxia or anoxia (miniMACS anaerobic workstation, Don Whitley Scientific, UK) for approximately 16 h.

### Cross-Streak assay on solid agar

Overnight cultures of *S. aureus* and *P. aeruginosa* grown separately under normoxia or anoxia were pelleted, resuspended in fresh broth and adjusted to an OD_470_ of 1.0. A sterile cotton swab was immersed in a given *P. aeruginosa* standardised culture and streaked horizontally across the surface of a LB agar plate. After air drying for 20 min, a sterile cotton swab was immersed in the adjusted *S. aureus* culture and cross-streaked vertically across the surface of the agar plate. Plates were then incubated either under normoxia or anoxia at 37 °C for 18 h, prior to being visually inspected for growth inhibition.

### Mono-culture and co-culture planktonic growth curves

All growth curve experiments were conducted in 250 mL conical flasks containing 50 mL of LBN broth at 37 °C under static conditions. Overnight cultures of *S. aureus* and *P. aeruginosa* CF isolates grown under normoxia or anoxia were pelleted, resuspended in fresh medium and adjusted to an OD_470_ of 1.0. For co-culture growth curves, the bacteria were inoculated at an equal ratio (1:1, *S. aureus* to *P. aeruginosa*) and incubated under static conditions at 37 °C for 24 h. Samples were taken at regular intervals, serially diluted in sterile PBS (Fisher, UK) and 20 µL spots plated onto *Pseudomonas* isolation agar (PIA) (Fisher Scientific, UK) and mannitol salt agar (MSA) (Fisher Scientific, UK) to allow species differentiation. The plates were incubated for approximately18 h, prior to enumerating the colony forming units (CFU/mL).

The competition index (CI) and Relative Increase Ratio (RIR) were calculated. The RIR was calculated on single growth curve data using the *P. aeruginosa-S. aureus* ratio at a given time point, divided by the same ratio at time point 0 h (inoculum). The same ratio was used to calculate the CI, although this used data from the mixed culture. A CI that differs statistically from the RIR indicates competition between the two organisms. This method was adapted from Macho *et al*.^91^.

### Mono-culture and co-culture biofilm formation

Overnight cultures of *S. aureus* and *P. aeruginosa* grown under normoxia or anoxia were centrifuged and adjusted to an OD_470_ of 1.0. Cultures were diluted tenfold and 100 µL added to the central wells of a 96-well tissue culture treated flat bottom plate, either individually or in a 1:1 ratio for an hour under static conditions, at 37 °C. An equal volume of broth was added to the individual culture to compensate for any dilution effect. After 60 min, the well contents were aspirated and replaced with fresh LBN broth. Plates were incubated for a further 24 h at 37 °C under static conditions. Following this, biofilms were washed twice using 200 µL of PBS, detached using 100 µL of 0.25% trypsin-EDTA (Fisher Scientific, UK), collected, vortexed for 70 s, serially diluted and plated onto PIA (Fisher, UK) and MSA (Fisher Scientific, UK). The plates were incubated for approximately18 h, prior to enumerating the colony forming units (CFU/mL).

### Preparation of *P. aeruginosa* cell-free culture supernatant

Overnight cultures of *P. aeruginosa* grown under normoxia or anoxia were centrifuged at 4,000 x *g* for 10 min at 4 °C. Each supernatant was sterile filtered with a low-binding 0.22 µm polyethersulfone membrane filter (Corning, USA) and stored at −20 °C until needed. To confirm sterility after each preparation, a small volume of supernatant was streaked onto LB agar plates and incubated for approximately 20 h prior to reading. For size exclusion experiments, 10 mL of cell-free supernatant was added to a 3 kDa molecular weight cut off protein concentrator (ThermoFisher, UK) and centrifuged at 4,000 × *g* for 1 h. Apical and basal volumes were subsequently added to 2.0 mL sterile microcentrifuge tubes (Fisher Scientific, UK). For heat-treatment, microcentrifuge tubes containing cell-free supernatant were added to a heat block and boiled at 95 °C for 10 mins, prior to cooling.

### Determination of total protease production

Protease production was determined using skimmed milk agar. Cell-free supernatants (40 µL) from overnight cultures were loaded into wells in agar plates and incubated at 37 °C for 24 h. Hydrolysis of the milk protein casein results in a clear zone surrounding the bacterial supernatant and would show evidence of protease production. LBN medium was also loaded as a negative control. Clearance zone diameters were measured with a ruler in millimetres (mm).

### Staphylolytic activity

This method was adapted from^92,93^. An overnight culture of *S. aureus* grown under static normoxia was centrifuged at 4,000 × *g* for 10 min at 4 °C, prior to the pellet being resuspended in 250 µL of 25 mM diethanolamine buffer (Sigma, UK), pH 9.5. The bacteria were then heated to 100 °C for 10 min, before being diluted to a final optimal density OD_595_ of 1.0. 400 µL of the adjusted heat-killed *S. aureus* was then added to each microtube (Fisher, UK). The cell-free supernatant from each *P. aeruginosa* isolate was diluted 1:10 with diethanolamine buffer, prior to 100 µL being added to the heat-killed *S. aureus*. Bacterial lysis was determined by measuring decreases in heat killed *S. aureus* OD_595_ after 60 min on a MultiSkan Go plate reader (ThermoFisher, UK). LBN broth alone was used as a control.

### Pyocyanin extraction and quantification

This method was adapted from^94,95^. Overnight cultures of *P. aeruginosa* grown under normoxia or anoxia were pelleted, resuspended in fresh medium and adjusted to an OD_470_ of 1.0. For single cultures, 500 µL of *P. aeruginosa* was then added to a 250 mL conical flask containing 50 mL of LBN broth (1:100 dilution). The flasks were then incubated at 37 °C for 24 h, under static normoxia or anoxia. To quantify pyocyanin production, bacterial cells were pelleted by 4,000 × *g* centrifugation for 25 min at 4 °C and the supernatant sterile filtered with a low-binding 0.22 µm polyethersulfone membrane filter (Corning, USA). 7.5 mL of the sterile supernatant was added to 4.5 mL of chloroform and vortexed for ten, 2 s intervals. The sample was centrifuged at 4,000 × *g* for 1 min at 4 °C, prior to 3 mL of the blue-green phase (chloroform phase) being aspirated into a new tube. 1.5 mL of 0.2 M hydrochloric acid was then added to the tube and vortexed again for ten, 2 s intervals, prior to centrifugation at 4,000 × *g* for 1 min at 4 °C. 100 µL of the pink coloured phase was then transferred into a 96-well plate in triplicate. 100 µL of hydrochloric acid was added in triplicate as a control. The plate was then read at OD_520_ and multiplied by the extinction co-efficient 17.072 to determine the concentration of pyocyanin per mL of supernatant^94^.

### Pyoverdine fluorescence spectroscopy

A total of 100 µL of each *P. aeruginosa* cell-free supernatant was added to a black 96-well plate (Griener Bio One, UK) in triplicate and read at excitation and emission wavelengths of 400/460 nm as performed previously^96,97^ on a Gemini XS Spectramax (Molecular Devices, USA) fluorescence plate reader. The background level of fluorescence was measured using 100 µL LBN broth only.

### Drop collapse assay

Cell-free supernatants from overnight cultures of *P. aeruginosa* were serially diluted (1:1) in sterile dH_2_O containing 0.005% crystal violet to aid visualisation. A total of 20 µL of each dilution (including neat supernatant) were spotted onto the underside of a lid of a 96-well plate and the plate titled at 90°, as performed previously^82^. The assay works on the principle that if the droplet contains surfactants, the drops spread. However, as the quantity of surfactants decrease by dilution, the droplet eventually beads up due to an increase in surface tension. Surfactant scores are equal to the reciprocal of the greatest dilution at which there was surfactant activity (a collapsed drop).

### Secretome analysis

Pooled overnight cultures of *P. aeruginosa* grown under either normoxia or anoxia were pelleted following centrifugation at 4,000 × *g* for 30 min at 4 °C. The supernatant containing extracellular proteins was sterile filtered through a 0.22 µm polyethersulfone membrane filter (Corning, USA). Supernatants were concentrated using Amicon 3 kDa cut-off filters (Millipore, UK) prior to precipitation with 25% trichloroacetic acid (TCA; w/v) for 15 min on ice. Proteins were pelleted at 14000 × *g* for 10 min, and pellets washed with acetone. Protein pellets were solubilised in 50 mM pH 7.4 Tris-HCl containing 2 mM CHAPS, 7 M urea and 7 M thiourea using an ultrasonicating probe (30 s sonication per cycle, 65% full power, 2 cycles), and quantified against a BSA calibration curve using the Bradford assay (BioRad, UK). Proteins (30 µg) were reduced in Laemmli buffer (Sigma Aldrich, UK) for 15 min at 65 °C, and separated by SDS-PAGE on a 10% gel. Separated proteins were stained with Coomassie G250 blue (0.5% w/v in 40% aqueous methanol and 10% glacial acetic acid) for 1 h and destained in an aqueous solution of 10% ethanol and 7.5% glacial acetic acid.

Protein in-gel digestion was performed following the method of Schevchenko *et al*.^98^, with minor modifications. Each sample lane was divided into 5 bands, gel bands were excised and diced in a clean polypropylene tube using a sterile scalpel. Gel pieces were subsequently destained with 50% acetonitrile in 50 mM ammonium bicarbonate, dehydrated with acetonitrile and vacuum dried. Proteins were in-gel digested using trypsin in 3 mM ammonium bicarbonate (25:1 protein to trypsin ratio; Trypsin Gold, sequencing, Promega, UK) coupled with shaking at 550 rpm at 37 °C overnight. Peptides were extracted for 15 min in an ultrasonic bath initially using a volume of acetonitrile equivalent to 50% of sample volume, two times with 150 µL of 50% acetonitrile in 50 mM ammonium bicarbonate. Finally 400 µL of pure acetonitrile was used to fully dehydrated the gel pieces and maximise peptide extraction. Each time the complete peptide extract was collected in a polypropylene tube. Finally, peptide extracts were vacuum dried and stored at −20 °C prior to analysis.

Samples were reconstituted in 50 µL of 3% aqueous acetonitrile and 0.1% formic acid for liquid chromatography-coupled tandem mass spectrometry (LC-MS/MS) analysis. Peptides were separated and analysed using an nLC system (Dionex 3000, ThermoScientific, UK) coupled to 5600 TripleTof (AB Sciex, UK) operating in information dependent (IDA) mode. Peptide solution (10 µL, ~5 pmol) was injected onto a trap column (PepMapTM, C18, 5 µm, 100 Å, 300 µm × 1 mm, ThermoScientific, UK) using 2% of eluent B (98% acetonitrile in aqueous 0.1% formic acid) at a flow rate of 30 µL/min. Peptides were subsequently separated on an analytical column (Acclaim^TM^, PepMap^TM^ C18, 3 µm, 100 Å, 75 µm × 150 mm, ThermoScientific, UK) with the following gradient: 0–3 min 2% B, 3–48 min 2–45% B, 48–52 min 45–90% B, 52–55 min 90% B, 55–70 min 2% B). Electrospray was formed by spraying the nLC eluate at 2500 V using a PicoTipTM emitter (New Objective, Germany). The 10 most intense ions from each MS survey scan were selected for MS/MS, while acquired ions were temporarily excluded from MS/MS acquisition for 30 s. The mass spectrometer was calibrated prior to acquisition to ensure a high mass accuracy (<10 ppm) on both MS and tandem mass spectrometry (MS/MS) levels. Relative quantification was done using Progenesis QI for proteomics software (version 4, Nonlinear Dynamics, UK). Only protein unique peptides were used for relative quantification. MS/MS data were searched using MasconDeamon (ver 2.5) against the SwissProt database, with the following search restriction parameters: mass tolerance of 0.1 Da for MS and 0.6 Da for MS/MS spectra, a maximum of 2 trypsin miscleavages, *Pseudomonas aeruginosa* taxonomy, variable modifications of methionine oxidation and cysteine carbamidomethylation.

### Bacterial motility

Swimming motility of *P. aeruginosa* was investigated using 0.3% (w/v) nutrient agar plates supplemented with nutrient broth and 1% (w/v) potassium nitrate. Swarming of *P. aeruginosa* was determined using 0.5% (w/v) nutrient agar plates supplemented with nutrient broth, dextrose and 1% (w/v) potassium nitrate. Overnight cultures of *P. aeruginosa* grown under normoxia or anoxia were adjusted to an OD_470_ of 1.0 with a total of 5 µL of culture added to the centre of each plate. To measure the effects of *S. aureus* on *P. aeruginosa* motility, a 1:100 diluted *S. aureus* cell-free culture supernatant was added to the agar plate. Plates were incubated under normoxia or anoxia for 24 h at 37 °C. The diameter of the zone travelled by *P. aeruginosa* was then measured using a ruler in mm.

### Statistical analysis

All results unless otherwise stated are expressed as mean ± S.E.M. Data for each experiment were collected from three independent experiments (*N* = 3), each performed in triplicate. All statistical analyses were performed using GraphPad Prism 6 software (Graphpad, La Jolla, CA, USA) with significance being set to *P* < 0.05. The specific tests used for each experiment are described in the figure legends.

## Supplementary information

Supplementary Information

## Data Availability

The datasets generated during and/or analysed during the current study are available from the corresponding author on reasonable request.
