# Supplementary material for: Anaerobiosis influences virulence properties of Pseudomonas aeruginosa cystic fibrosis isolates and the interaction with Staphylococcus aureus
Source: Sci Rep. 2019 May 1;9:6748. doi: 10.1038/s41598-019-42952-x (PMC6494883; doi:10.1038/s41598-019-42952-x)
Supplement: Supplementary file 1 — Supplementary Information [file 41598_2019_42952_MOESM1_ESM.pdf]

Anaerobiosis influences virulence properties of *Pseudomonas aeruginosa* cystic fibrosis isolates and the interaction with *Staphylococcus aureus*

Ross Pallett[1], Laura J. Leslie[2], Peter. A. Lambert[1], Ivana Milic[1], Andrew Devitt[1] and Lindsay J. Marshall[1]

[1]School of Life and Health Sciences, Aston University, Birmingham, UK
[2]Aston Institute of Materials Research, School of Engineering and Applied Science, Aston University, Birmingham, UK

__Supplementary information__

Inhibition of *S. aureus* growth by *P. aeruginosa* CF clinical isolates

Reference strain PAO1 and the eight CF clinical isolates of *P. aeruginosa* were tested for growth inhibition of *S. aureus* under normoxia, on an agar plate based cross-streak assay. After standardising the bacterial densities of overnight cultures, *P. aeruginosa* isolates were streaked horizontally across the surface of the agar plate and allowed to dry, prior to cross-streaking *S. aureus* vertically. Plates were then incubated at 37 °C and 5% CO_2 for 24 h, prior to the intersection of the cross-streak being visually inspected.

As shown in **Supplementary Fig. S1** following 24 h incubation, PAO1 and CF isolates 1, 2, 4, 5 and 7 all inhibited *S. aureus* growth in co-culture (dashed white box). Conversely, CF isolates 3, 6 and 8 had no effect upon *S. aureus* growth.

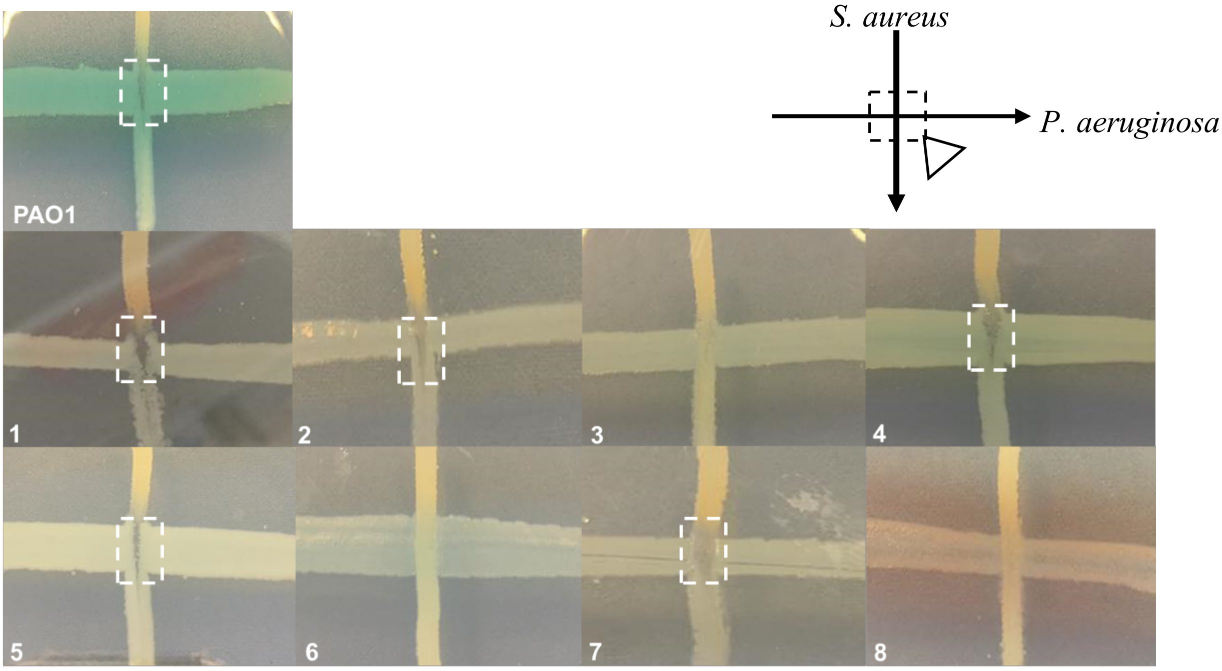

Supplementary Figure S1. Cross-streak growth inhibition assay between CF clinical isolates of *P. aeruginosa* with *S. aureus* on solid LB agar plates. Overnight bacterial cultures of *S. aureus* and *P. aeruginosa* were standardised to an OD_{470} of 1.0. *P. aeruginosa* was inoculated horizontally across the centre of the agar plate and allowed to dry. *S. aureus* was subsequently streaked vertically. Plates were incubated overnight, prior to being read. Zones of growth inhibition are marked by white dashed boxes. Images are representative of three individual experiments (*N*=3), each performed in duplicate.

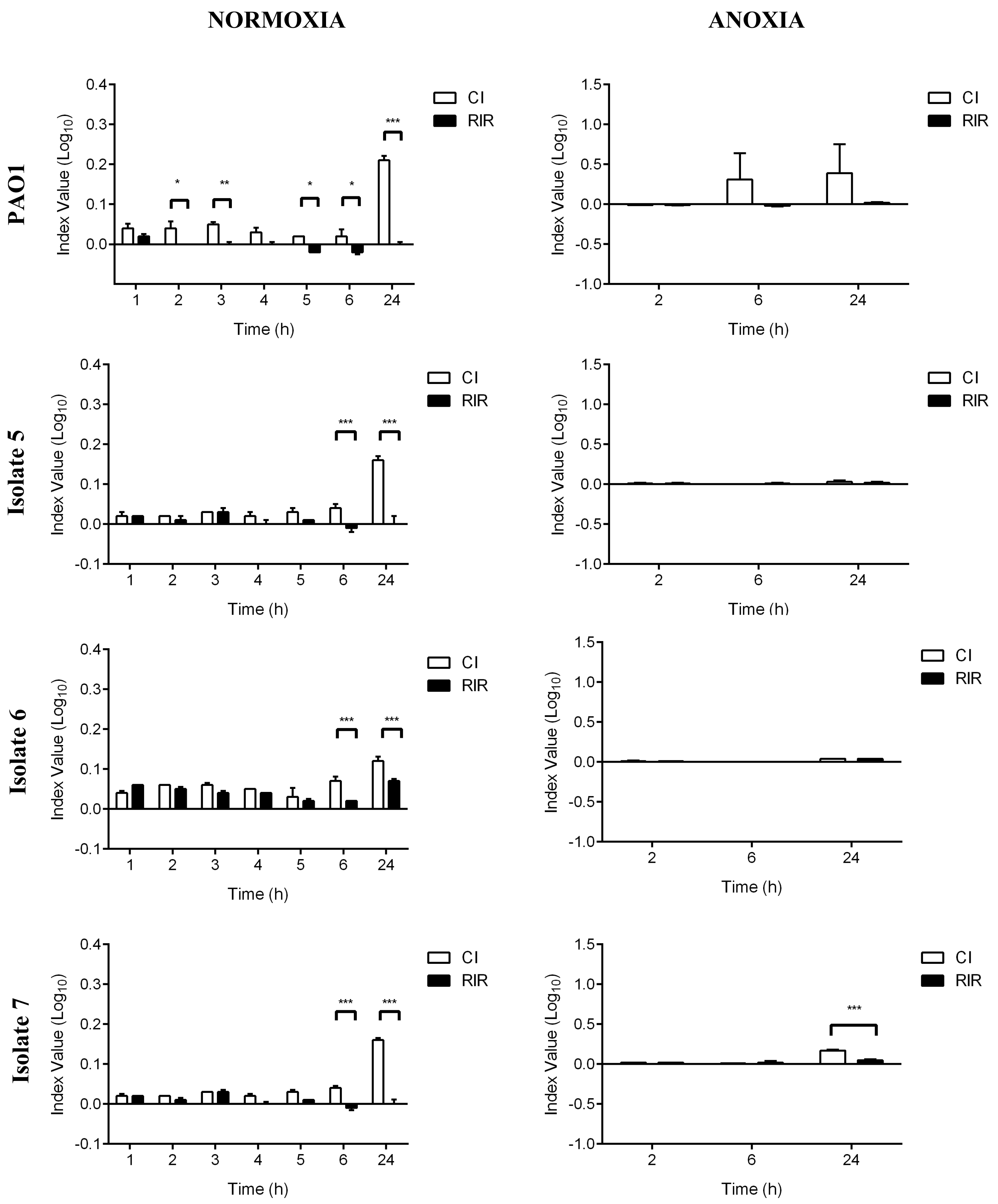

Supplementary Figure S2. ***S. aureus*** **and** ***P. aeruginosa*** **mono and co-culture growth curves under normoxia and anoxia.** Each value represents the mean of the CI and RIR ± S.E.M calculated from single and dual cultures. Each value is the mean of three independent experiments (N=3), each performed in triplicate. Statistical differences were determined using two-way ANOVA comparing the CI to the RIR, with Bonferroni's multiple comparisons *post-hoc* test. *P<0.05, **P<0.01 and ***P<0.001.

Supplementary Table S1. Summary of planktonic co-culture and mixed species biofilm competition data for *P. aeruginosa* PAO1 and CF isolates 5, 6 and 7 following growth under normoxia and anoxia. Data is obtained from Figure 1, Figure 2 and Supplementary Fig. S2.

**Isolate**	**Outcompetes *S. aureus* in planktonic co-culture**		**Outcompetes *S. aureus* in mixed species biofilm**	
	**Normoxia**	**Anoxia**	**Normoxia**	**Anoxia**
**PAO1**	Yes	No	Yes	No
**Isolate 5**	Yes	No	Yes	No
**Isolate 6**	Yes	No	No	No
**Isolate 7**	Yes	Yes	Yes	Yes

Supplementary Table S2. Cell-free secretome of *P. aeruginosa* PAO1 following growth under normoxia and anoxia. Data represents the average normalised abundances and fold increase from *N*=1. A fold change of 1.0 is indicative of no change between normoxia and anoxia. O1A = *P. aeruginosa* PAO1 grown under anoxia. O1O = *P. aeruginosa* PAO1 grown under normoxia.

Accession	Peptides (unique)	Score	Description	Average Normalised Abundances		Fold increase	
				O1A	O1O	O1A	O1O
CH60_PSEA7	63 (6)	4162.84	60 kDa chaperonin	3264.99	2802.59	1.16	
NIRS_PSEAE	49 (43)	3286.73	Nitrite reductase	5.70E+04	1.61E+05		2.82
EFG1_PSEAE	57 (14)	3226.98	Elongation factor G 1	4.38E+04	4.35E+04	1.01	
CATA_PSEAE	42 (24)	2795.92	Catalase	1.05E+05	2.78E+05		2.65
DNAK_PSEAB	41 (17)	2657.45	Chaperone protein DnaK	5.39E+04	1.30E+04	4.15	
AMPC_PSEAE	36 (31)	2369.11	Beta-lactamase	1.24E+05	1.76E+05		1.42
DLDH1_PSEAE	35 (22)	2195.21	Dihydrolipoyl dehydrogenase	3351.71	6.00E+04		17.90
RPOB_PSEAB	46 (10)	2072.97	DNA-directed RNA polymerase subunit beta	1.62E+04	5416.61	2.99	
EFTU_PSEAB	28 (9)	1999.82	Elongation factor Tu	4.47E+04	1914.24	23.35	
PNP_PSEAB	37 (12)	1995.68	Polyribonucleotide nucleotidyltransferase	1.26E+04	2.42E+04		1.92
CLPB_PSEAE	38 (19)	1974.4	Chaperone protein ClpB	3654.37	1.63E+04		4.46
MASZ_PSEAB	34 (11)	1917.01	Malate synthase G	2.20E+04	3.21E+04		1.46
FLID2_PSEAE	29 (24)	1882.05	B-type flagellar hook-associated protein 2	6164.14	3.39E+04		5.50
RPOC_PSEA7	34 (6)	1843.37	DNA-directed RNA polymerase subunit beta'	2.21E+04	485.62	45.51	
NOSZ_PSEAE	35 (19)	1841.49	Nitrous-oxide reductase	4.78E+04	4.24E+04	1.13	
ATPB_PSEAB	30 (6)	1817.99	ATP synthase subunit beta	2724.83	1.03E+04		3.78
ODP1_PSEAE	38 (32)	1800.49	Pyruvate dehydrogenase E1 component	4.85E+04	1610.8	30.11	
Y807_PSEAB	26 (19)	1772.35	Putative prophage major tail sheath protein	1.22E+05	5.25E+04	2.32	
FLICB_PSEAE	23 (15)	1715.76	B-type flagellin	7976.19	6.00E+04		7.52
DHE2_PSEAE	38 (29)	1674.5	NAD-specific glutamate dehydrogenase	1.64E+04	4843.73	3.39	
SAHH_PSEAB	32 (14)	1543.97	Adenosylhomocysteinase	1.30E+04	4.08E+04		3.14
TIG_PSEAB	26 (2)	1463.62	Trigger factor	6632.97	304.39	21.79	

ACNB_PSEAE	27 (24)	1463.21	Aconitate hydratase B	3.22E+04	3138.99	10.26	
SYI_PSEAE	29 (5)	1455.14	Isoleucine--tRNA ligase	2541.73	6700.29		2.64
FABY_PSEAE	21 (18)	1447.88	Beta-ketoacyl-[acyl-carrier-protein] synthase FabY	2307.24	2.14E+04		9.28
ODP2_PSEAE	23 (15)	1444.42	Dihydrolipoyllysine-residue acetyltransferase component of pyruvate dehydrogenase complex	4.02E+04	1.10E+04	3.65	
BRAC_PSEAE	22 (15)	1427.49	Leucine-, isoleucine-, valine-, threonine-, and alanine-binding protein	1.02E+04	8.87E+04		8.70
DAVT_PSEAE	24 (14)	1418.89	5-aminovalerate aminotransferase DavT	4.70E+04	5.18E+04		1.10
ATPA_PSEAB	24 (4)	1415.29	ATP synthase subunit alpha	5576.59	1.38E+04		2.47
OTCC_PSEAE	24 (13)	1399.22	Ornithine carbamoyltransferase, catabolic	5.79E+04	4.30E+04	1.35	
IDH_PSEAB	30 (22)	1376.12	Isocitrate dehydrogenase [NADP]	3.28E+04	4364.97	7.51	
Y421_PSEA8	21 (18)	1351.24	UPF0312 protein PLES_04211+D263:D323	2.03E+04	5.70E+04		2.81
AMPA_PSEAE	18 (5)	1328.21	Cytosol aminopeptidase	3646.13	1.86E+04		5.10
DAVD_PSEAE	27 (17)	1301.4	Glutarate-semialdehyde dehydrogenase DavD	3.93E+04	2.47E+04	1.59	
ATOB_PSEAE	25 (22)	1297.87	Acetyl-CoA acetyltransferase	4060.25	5.38E+04		13.25
HUTU_PSEAB	23 (9)	1293.98	Urocanate hydratase	5284.34	4.26E+04		8.06
HTPG_PSEAE	23 (2)	1290.88	Chaperone protein HtpG	1.06E+04	9989.09	1.06	
ARUC_PSEAE	21 (18)	1278.7	Succinylornithine transaminase/acetylornithine aminotransferase	6341.14	3.00E+04		4.73
ARCA_PSEAE	21 (13)	1239.64	Arginine deiminase	3.89E+04	1573.49	24.72	
AMPA_PSEAB	16 (1)	1227.93	Probable cytosol aminopeptidase	642.25	2534.71		3.95
ASPQ_PSEAE	22 (14)	1203.8	Glutaminase-asparaginase	1298.62	8.62E+04		66.38
RECA_PSEAB	17 (3)	1198.9	Protein RecA	7568.96	105.58	71.69	
SYL_PSEAB	22 (11)	1192.86	Leucine--tRNA ligase	2078.07	1.38E+04		6.64
CISY_PSEAE	20 (14)	1141.85	Citrate synthase	5384.88	1.99E+04		3.70
SYV_PSEAE	27 (12)	1113.4	Valine--tRNA ligase	1908.66	2.90E+04		15.19
Y3922_PSEAE	21 (17)	1109.43	Uncharacterized protein PA3922	2393.8	3.89E+04		16.25
EFG2_PSEAE	19 (3)	1102.25	Elongation factor G 2	1179.76	1562.63		1.32
SYI_PSEAB	25 (3)	1083.23	Isoleucine--tRNA ligase	482.49	486.09		1.01
GYRA_PSEAE	20 (12)	1042.48	DNA gyrase subunit A	6841	8170.72		1.19
DSBA_PSEAB	16 (12)	1003.81	Thiol:disulfide interchange protein DsbA	2.70E+04	5.85E+04		2.17
PGK_PSEAB	19 (10)	975.67	Phosphoglycerate kinase	4638.13	1.76E+04		3.79
ETFB_PSEAE	13 (12)	963.33	Electron transfer flavoprotein subunit beta	7.26E+04	2.15E+04	3.38	

AZUR_PSEAE	13 (13)	943.35	Azurin	1.10E+05	4.98E+05		4.53
PCKA_PSEAE	19 (6)	936.19	Phosphoenolpyruvate carboxykinase (ATP)	2.17E+04	1.24E+04	1.75	
RS1_PSEAE	20 (17)	923.42	30S ribosomal protein S1	3.49E+04	3750.78	9.30	
ODB2_PSEAE	18 (13)	917.1	Lipoamide acyltransferase component of branched-chain alpha-keto acid dehydrogenase complex	1686.32	3.11E+04		18.44
EFTS_PSEAB	17 (5)	886.77	Elongation factor Ts	3.65E+04	1.01E+04	3.61	
GCSP1_PSEAE	19 (6)	868.57	Glycine dehydrogenase (decarboxylating) 1	6384.8	2218.39	2.88	
GATA_PSEAE	14 (3)	867.68	Glutamyl-tRNA(Gln) amidotransferase subunit A	845.11	3118.8		3.69
GPMI_PSEAE	15 (6)	852.95	2,3-bisphosphoglycerate-independent phosphoglycerate mutase	4117.86	8031.69		1.95
P5217_PSEAE	15 (9)	840.95	Probable binding protein component of ABC iron transporter PA5217	5646.18	4.77E+04		8.45
METE_PSEAE	20 (10)	835.19	5-methyltetrahydropteroyltriglutamate--homocysteine methyltransferase	5653.51	1.30E+04		2.30
PHHC_PSEAE	15 (12)	832.54	Aromatic-amino-acid aminotransferase	3985.79	1.60E+04		4.01
DPO3B_PSEAE	12 (7)	816.52	Beta sliding clamp	8036.12	1.20E+04		1.49
ELAS_PSEAE	17 (10)	793.21	Elastase	6184.42	1.25E+05		20.21
SUCC_PSEAE	17 (4)	770.47	Succinate--CoA ligase [ADP-forming] subunit beta	2.36E+04	8540.9	2.76	
FUMC2_PSEAE	14 (12)	770.14	Fumarate hydratase class II 2	1581.58	1.45E+04		9.17
DPO1_PSEAE	16 (15)	769.54	DNA polymerase I	5727.45	2258.88	2.54	
FUMC1_PSEAE	10 (8)	757.77	Fumarate hydratase class II 1	1306.67	9571.43		7.33
RPOA_PSEAB	17 (3)	755.89	DNA-directed RNA polymerase subunit alpha	5807.02	3456.45	1.68	
AMPA_PSEA7	14 (1)	740.85	Probable cytosol aminopeptidase	362.07	2347.13		6.48
IF2_PSEA7	20 (4)	714.05	Translation initiation factor IF-2	1891.11	716.75	2.64	
SYR_PSEA7	14 (5)	706.2	Arginine--tRNA ligase	3650.91	323.37	11.29	
DCTP_PSEAE	17 (11)	700.98	C4-dicarboxylate-binding periplasmic protein DctP	2.32E+04	2.90E+04		1.25
ARCC_PSEAE	14 (12)	678.24	Carbamate kinase	8.91E+04	5654.23	15.76	
RL1_PSEA7	11 (5)	675.4	50S ribosomal protein L1	2.48E+04	256.9	96.54	
SUCD_PSEAE	13 (10)	675.23	Succinate--CoA ligase [ADP-forming] subunit alpha	9.12E+04	3.58E+04	2.55	
NADE_PSEAB	13 (2)	669.36	NH(3)-dependent NAD(+) synthetase	1.40E+04	1.21E+04	1.16	
PORF_PSEAE	12 (10)	668.37	Outer membrane porin F	2.28E+04	2.32E+04		1.02
SURA_PSEAE	11 (10)	664.87	Chaperone SurA	4289.48	6630.36		1.55
STHA_PSEAB	13 (9)	657.46	Soluble pyridine nucleotide transhydrogenase	5524.25	6580.99		1.19

RL5_PSEA8	11 (3)	655.31	50S ribosomal protein L5	6.44E+04	1.79E+04	3.60	
ACNA_PSEAE	12 (6)	654.55	Aconitate hydratase A	7970.97	1464.29	5.44	
RL6_PSEA7	12 (4)	640.47	50S ribosomal protein L6	4.33E+04	684.66	63.24	
BFR_PSEAE	14 (10)	634.92	Bacterioferritin	1.57E+04	1.88E+04		1.20
ILVC_PSEAB	16 (8)	630.16	Ketol-acid reductoisomerase (NADP(+))	2977.29	2952.71	1.01	
ODO2_PSEAE	11 (7)	630.11	Dihydrolipoyllysine-residue succinyltransferase component of 2-oxoglutarate dehydrogenase complex	1.43E+04	6441.83	2.22	
ARGC_PSEAE	13 (4)	627.95	N-acetyl-gamma-glutamyl-phosphate reductase	1987.28	5454.47		2.74
TAL_PSEAB	12 (10)	617.93	Transaldolase	8172.41	1.77E+04		2.17
RRF_PSEAB	12 (11)	616.96	Ribosome-recycling factor	1.53E+04	3244.5	4.72	
SYP_PSEAE	11 (4)	615.53	Proline--tRNA ligase	5671.83	959.23	5.91	
AHPC_PSEAB	13 (12)	611.95	Alkyl hydroperoxide reductase C	8.73E+04	2.40E+05		2.75
GLN1B_PSEAE	10 (9)	611.68	Glutamine synthetase	1.97E+04	2948.92	6.68	
METK_PSEAB	15 (4)	607.62	S-adenosylmethionine synthase	7311.51	2.05E+04		2.80
MMSA_PSEAE	11 (10)	604.8	Methylmalonate-semialdehyde dehydrogenase [acylating]	8494.93	1.85E+04		2.18
TPX_PSEAE	8 (6)	603.69	Thiol peroxidase	1.36E+04	4429.23	3.07	
RECA_PSEST	10 (1)	597.49	Protein RecA	3700.22	108.16	34.21	
GGT_PSEAE	12 (7)	584.06	Gamma-glutamyltranspeptidase	1205.15	1.31E+04		10.87
THIO_PSEAE	11 (9)	580.98	Thioredoxin	3.26E+04	9.35E+04		2.87
GSHR_PSEAE	13 (10)	569.23	Glutathione reductase	1704.51	9414.46		5.52
DAPA_PSEAE	10 (6)	568.87	4-hydroxy-tetrahydrodipicolinate synthase	1.51E+04	3.11E+04		2.06
ACSA1_PSEAE	15 (11)	568.43	Acetyl-coenzyme A synthetase 1	4129.3	2046.61	2.02	
DHAS_PSEAE	11 (6)	565.84	Aspartate-semialdehyde dehydrogenase	2629.25	9556.47		3.63
SPUD_PSEAB	14 (13)	555.06	Putrescine-binding periplasmic protein SpuD	1.22E+04	5.13E+04		4.20
ETFA_PSEAE	9 (9)	554.49	Electron transfer flavoprotein subunit alpha	5.30E+04	2.45E+04	2.16	
DAPB_PSEAB	10 (4)	552.17	4-hydroxy-tetrahydrodipicolinate reductase	1.09E+04	9560.14	1.14	
NADE_PSEA7	11 (1)	549.57	NH(3)-dependent NAD(+) synthetase	1.22E+04	1.39E+04		1.14
ENO_PSEAB	12 (8)	549.46	Enolase	9371.84	146.45	63.99	
HEM6_PSEA7	10 (7)	546.18	Oxygen-dependent coproporphyrinogen-III oxidase OS=Pseudomonas aeruginosa (strain PA7)	7.94E+04	4.65E+04	1.71	
LAP_PSEAB	10 (7)	536.63	Aminopeptidase	361.66	1.39E+04		38.43
SYY2_PSEAE	14 (3)	534.97	Tyrosine--tRNA ligase 2	3352.67	2189.08	1.53	

CARB_PSEAE	17 (5)	534.96	Carbamoyl-phosphate synthase large chain	4274.34	56.71	75.37	
BAUC_PSEAE	13 (8)	534.7	Putative 3-oxopropanoate dehydrogenase	1.16E+04	5702.66	2.03	
RPOD_PSEAE	12 (5)	529.54	RNA polymerase sigma factor RpoD	3536.64	449.03	7.88	
AROE_PSEAE	9 (6)	526.56	Shikimate dehydrogenase (NADP(+))	2.30E+04	4361.54	5.27	
HEM2_PSEAE	11 (7)	526.39	Delta-aminolevulinic acid dehydratase	7392.52	2.04E+04		2.76
MURD_PSEAE	10 (8)	521.15	UDP-N-acetylmuramoylalanine--D-glutamate ligase	2282.36	7232.95		3.17
ILVD_PSEAB	9 (2)	515.39	Dihydroxy-acid dehydratase	3035.39	1601.61	1.90	
LOLA_PSEAB	12 (10)	512.57	Outer-membrane lipoprotein carrier protein	4338.49	8008.01		1.85
PHZB1_PSEAE	11 (3)	511.74	Phenazine biosynthesis protein PhzB 1	3693.52	3.85E+04		10.42
CBPD_PSEAE	9 (8)	505.38	Chitin-binding protein CbpD	2281.24	7.36E+04		32.26
PYRG_PSEAB	8 (4)	498.33	CTP synthase	2109.01	238.92	8.83	
ISCS_PSEAB	11 (1)	494.04	Cysteine desulfurase IscS	3242.41	1215.2	2.67	
RL9_PSEA7	9 (5)	491.4	50S ribosomal protein L9	2.60E+04	2342.63	11.10	
ACCC_PSEAE	11 (8)	490.03	Biotin carboxylase	5405.52	684.58	7.90	
ACKA_PSEAB	12 (4)	489.17	Acetate kinase	1576.04	5418.9		3.44
SYK_PSEAB	11 (4)	487.08	Lysine--tRNA ligase	3734.84	702.91	5.31	
FPTA_PSEAE	9 (9)	482.99	Fe(3+)-pyochelin receptor	136.64	3619.43		26.49
GLMS_PSEAE	11 (7)	480.61	Glutamine--fructose-6-phosphate aminotransferase [isomerizing]	3509.06	2118.11	1.66	
RL25_PSEA8	8 (7)	479.28	50S ribosomal protein L25	3.44E+04	1172.63	29.34	
ASPA_PSEAE	11 (7)	472.83	Aspartate ammonia-lyase	7114.87	478.9	14.86	
PAL_PSEAE	8 (6)	468.2	Peptidoglycan-associated lipoprotein	4700	1.98E+04		4.21
AK_PSEAE	10 (4)	467.18	Aspartokinase	6926.54	1.29E+04		1.86
HISX_PSEAE	8 (5)	466.08	Histidinol dehydrogenase	2434.54	243.71	9.99	
IPYR_PSEAE	9 (7)	462.95	Inorganic pyrophosphatase	7680.67	1.38E+04		1.80
HIS1_PSEA8	8 (4)	449.5	ATP phosphoribosyltransferase	1569.01	2027.14		1.29
PROA_PSEAE	7 (4)	448.62	Gamma-glutamyl phosphate reductase	2913.78	8635.6		2.96
PYS2_PSEAE	12 (8)	440.27	Pyocin-S2	6820.92	227.67	29.96	
TOLB_PSEAE	9 (6)	437.59	Protein TolB	917.54	3460.78		3.77
Y1579_PSEAE	10 (9)	433.91	Uncharacterized protein PA1579	3092.79	1.61E+04		5.21
PURE_PSEAE	6 (5)	431.48	N5-carboxyaminoimidazole ribonucleotide mutase	6625.53	8908.4		1.34

GREA_PSEAE	8 (6)	429.23	Transcription elongation factor GreA 1	6852.59	282.36	24.27	
ZIPA_PSEAB	8 (7)	428.43	Cell division protein ZipA	1391.64	7446.9		5.35
PPSA_PSEAB	10 (8)	422.12	Phosphoenolpyruvate synthase	4710.88	484.98	9.71	
RS2_PSEA8	9 (3)	417.1	30S ribosomal protein S2	5398.89	29.49	183.08	
HUTH_PSEAB	8 (6)	404.58	Histidine ammonia-lyase	303.11	2950.2		9.73
PHZB2_PSEAB	9 (3)	400.05	Phenazine biosynthesis protein PhzB 2	819.05	1.01E+04		12.33
GATB_PSEAB	10 (3)	399.14	Aspartyl/glutamyl-tRNA(Asn/Gln) amidotransferase subunit B	1063.97	1.03E+04		9.68
CYSNC_PSEAE	8 (6)	399.14	Bifunctional enzyme CysN/CysC	256.56	4029		15.70
SSB_PSEAE	8 (4)	398.46	Single-stranded DNA-binding protein	1.76E+04	1.25E+04	1.41	
RS10_PSEA7	7 (4)	385.93	30S ribosomal protein S10	1.59E+04	619.34	25.67	
SPUE_PSEAE	12 (9)	385.69	Spermidine-binding periplasmic protein SpuE	3858.28	1.04E+04		2.70
MTIP_PSEAE	8 (7)	383.15	S-methyl-5'-thioinosine phosphorylase	1629.94	8302.92		5.09
CYSD_PSEAB	11 (2)	381.18	Sulfate adenylyltransferase subunit 2	4382.24	948.13	4.62	
PURA_PSEAB	8 (4)	375.32	Adenylosuccinate synthetase	2205.54	1766.46	1.25	
PPIA_PSEAE	7 (7)	375.06	Peptidyl-prolyl cis-trans isomerase A	5579.05	4801.79	1.16	
PYRC_PSEAB	7 (7)	374.63	Dihydroorotase	1907.53	1.11E+04		5.82
GUAA_PSEAB	10 (4)	374.54	GMP synthase [glutamine-hydrolyzing]	1.17E+04	3363.19	3.48	
ALGC_PSEAB	9 (7)	374.19	Phosphomannomutase/phosphoglucomutase	6450.39	5666.1	1.14	
RS8_PSEA7	6 (4)	371.88	30S ribosomal protein S8	1.52E+04	511.84	29.70	
MMSB_PSEAE	9 (7)	370.14	3-hydroxyisobutyrate dehydrogenase	2.29E+04	1.52E+04	1.51	
AAT_PSEAE	7 (7)	369.8	Aspartate aminotransferase	2845.42	1440.21	1.98	
HISZ_PSEAB	8 (2)	367.57	ATP phosphoribosyltransferase regulatory subunit	637.16	2875.68		4.51
RL3_PSEA7	10 (8)	367.52	50S ribosomal protein L3	4.71E+04	1.01E+04	4.66	
SODF_PSEAE	7 (5)	365.03	Superoxide dismutase [Fe]	4557.07	1.49E+04		3.27
FABG_PSEAE	7 (4)	363.29	3-oxoacyl-[acyl-carrier-protein] reductase FabG	4280.9	750.09	5.71	
EDD_PSEAE	8 (6)	363.17	Phosphogluconate dehydratase	2545.09	4853.12		1.91
GRPE_PSEAB	8 (3)	360.42	Protein GrpE	1.06E+04	192.55	55.05	
RS5_PSEA7	5 (2)	359.03	30S ribosomal protein S5	2189.79	189.49	11.56	
BAUA_PSEAE	10 (7)	356.41	Beta-alanine--pyruvate aminotransferase	7797.8	3972.97	1.96	
PUR8_PSEAE	9 (9)	356.25	Adenylosuccinate lyase	719.1	2851		3.96

ILVE_PSEAE	8 (7)	353.35	Branched-chain-amino-acid aminotransferase	1.11E+04	4.01E+04		3.61
NUSG_PSEAE	6 (1)	352.87	Transcription termination/antitermination protein NusG	3769.35	295.54	12.75	
ARGB_PSEAB	8 (2)	350.4	Acetylglutamate kinase	1407.19	843.43	1.67	
MURE_PSEAE	7 (3)	347.91	UDP-N-acetylmuramoyl-L-alanyl-D-glutamate--2,6-diaminopimelate ligase	233.14	2707.22		11.61
G6PI_PSEA8	8 (6)	347.69	Glucose-6-phosphate isomerase	900.53	7978.78		8.86
NOSZ_PSEAI	11 (1)	345.38	Nitrous-oxide reductase	102.48	816.92		7.97
DAPD_PSEAE	7 (7)	345.26	2,3,4,5-tetrahydropyridine-2,6-dicarboxylate N-succinyltransferase	4574.06	1041.22	4.39	
TOP1_PSEAE	9 (7)	342.14	DNA topoisomerase 1	5434.46	1.02E+04		1.88
PYRX_PSEAE	8 (5)	337.5	Dihydroorotase-like protein	1353.08	1692.15		1.25
SYGB_PSEAB	5 (4)	335.09	Glycine--tRNA ligase beta subunit	3381.6	727.16	4.65	
KAD_PSEAB	6 (3)	332.88	Adenylate kinase	9846.38	957.53	10.28	
FABZ_PSEAB	9 (7)	326.25	3-hydroxyacyl-[acyl-carrier-protein] dehydratase FabZ	2523.95	1.17E+04		4.64
APRA_PSEAE	6 (6)	324.37	Serralysin	665.08	3858.4		5.80
RL18_PSEA7	7 (3)	323.31	50S ribosomal protein L18	4313.93	2363.28	1.83	
SECB_PSEAB	5 (5)	321.76	Protein-export protein SecB	2.09E+04	7637.23	2.74	
RS7_PSEA7	8 (2)	319.43	30S ribosomal protein S7	8164.5	495.27	16.48	
THIG_PSEAB	6 (6)	313.52	Thiazole synthase	1.95E+04	2593.74	7.52	
SECA_PSEAB	8 (3)	312.31	Protein translocase subunit SecA	2165.09	147.59	14.67	
PANB2_PSEAE	5 (4)	310.09	3-methyl-2-oxobutanoate hydroxymethyltransferase 2	4646.27	7204.85		1.55
GSA_PSEAB	6 (3)	308.59	Esterase EstA	2324.77	566.97	4.10	
CYNS_PSEAE	7 (4)	308.59	Cyanate hydratase	767.64	3108.42		4.05
PUR9_PSEA8	9 (8)	308.29	Bifunctional purine biosynthesis protein PurH	2924.4	1755.24	1.67	
OPGG_PSEAB	7 (4)	307.03	Glucans biosynthesis protein G	4710.2	946.68	4.98	
RL19_PSEA7	5 (4)	301.84	50S ribosomal protein L19	1.15E+04	1085.68	10.59	
ATPG_PSEAB	11 (5)	301.6	ATP synthase gamma chain	2588.3	3084.65		1.19
NUOG_PSEAE	7 (4)	301.58	NADH-quinone oxidoreductase subunit G	1101.84	216.48	5.09	
CYC4_PSEAE	6 (4)	301.26	Cytochrome c4	5569.33	2223.33	2.50	
PYRE_PSEAB	6 (4)	296.96	Orotate phosphoribosyltransferase	3017.47	2618.6	1.15	
SYW_PSEAE	7 (6)	293.25	Tryptophan--tRNA ligase	2414.07	1554.06	1.55	
SYGA_PSEAB	5 (5)	291.48	Glycine--tRNA ligase alpha subunit	8805.31	979.9	8.99	

PUR4_PSEAE	10 (4)	288.88	Phosphoribosylformylglycinamidine synthase	5666.64	130.87	43.30	
RL7_PSEA7	9 (6)	288.85	50S ribosomal protein L7/L12	2.08E+04	7310.6	2.85	
GMHA_PSEAB	5 (5)	286.81	Phosphoheptose isomerase	4292.04	1.20E+04		2.80
UVRA_PSEAE	7 (4)	285.55	UvrABC system protein A	1266.38	693.49	1.83	
FUR_PSEAE	3 (2)	283.46	Ferric uptake regulation protein	1.29E+04	5121.44	2.52	
PDXJ_PSEAB	5 (2)	280.06	Pyridoxine 5'-phosphate synthase	1008.04	2902.45		2.88
SUHB_PSEAE	8 (6)	277.61	Inositol-1-monophosphatase	7710.16	4900.43	1.57	
CH10_PSEA7	5 (5)	273.93	10 kDa chaperonin	3.48E+04	2245.9	15.49	
FABB_PSEAB	6 (5)	273.58	3-oxoacyl-[acyl-carrier-protein] synthase 1	3605.89	1.24E+04		3.44
RL2_PSEA7	7 (3)	272.99	50S ribosomal protein L2	7562.94	1.39E+04		1.84
HPPD_PSEAE	6 (4)	270.19	4-hydroxyphenylpyruvate dioxygenase	2032.18	324.02	6.27	
NDK_PSEAB	9 (6)	269.16	Nucleoside diphosphate kinase	4.22E+04	5.43E+04		1.29
RL21_PSEA8	5 (5)	267.51	50S ribosomal protein L21	1.38E+04	189.32	72.89	
PH4H_PSEAE	4 (4)	265.85	Phenylalanine-4-hydroxylase	8581.27	8653.33		1.01
EFP_PSEAB	5 (3)	265.36	Elongation factor P	1.78E+04	1550.42	11.48	
GAP2_PSEAB	8 (6)	264.25	Glyceraldehyde-3-phosphate dehydrogenase-like protein	8538.69	6152.27	1.39	
MUCB_PSEAE	4 (3)	264.07	Sigma factor AlgU regulatory protein MucB	86.16	3448.83		40.03
PHS_PSEAB	5 (3)	262.73	Putative pterin-4-alpha-carbinolamine dehydratase	1.43E+04	3.91E+04		2.73
AHPF_PSEAE	5 (5)	261.53	Alkyl hydroperoxide reductase subunit F	129.79	3833.34		29.53
NUOCD_PSEAB	7 (4)	260.42	NADH-quinone oxidoreductase subunit C/D	1490.65	3686.29		2.47
IF3_PSEAE	4 (3)	258.3	Translation initiation factor IF-3	5083.4	1056.79	4.81	
FLICA_PSEAI	3 (1)	257.94	A-type flagellin	1183.32	1826.27		1.54
RISB_PSEAB	6 (6)	254.01	6,7-dimethyl-8-ribityllumazine synthase	8924.42	5092.8	1.75	
PSTS_PSEAB	5 (5)	249.8	Phosphate-binding protein PstS	3406.36	8035		2.36
DEF_PSEAE	4 (4)	248.69	Peptide deformylase	5040.04	5353.06		1.06
AGUA_PSEAE	4 (2)	245.35	Agmatine deiminase	719.85	2955.33		4.11
GCH12_PSEAE	4 (4)	244.05	GTP cyclohydrolase 1 2	650.87	4195.99		6.45
ISPF_PSEAE	4 (1)	242.82	2-C-methyl-D-erythritol 2,4-cyclodiphosphate synthase	1180.02	16.56	71.26	
TRPC_PSEAE	6 (2)	242.12	Indole-3-glycerol phosphate synthase	4666.46	1948.68	2.39	
SODM_PSEAE	6 (6)	240.83	Superoxide dismutase [Mn]	377.96	1.00E+04		26.46

CY551_PSEAE	4 (4)	237.68	Cytochrome c-551	4635	1.83E+04		3.95
FABI_PSEAE	5 (4)	236.8	Enoyl-[acyl-carrier-protein] reductase [NADH] FabI	471.25	2315.3		4.91
GCSP1_PSEPK	8 (1)	236.71	Glycine dehydrogenase (decarboxylating) 1	1826.72	1.07E+04		5.86
T23O_PSEAB	4 (2)	234.88	Tryptophan 2,3-dioxygenase	849.4	5067.08		5.97
LEU1_PSEA7	5 (4)	229.55	2-isopropylmalate synthase	2290.18	1607.79	1.42	
DNAK_THISH	5 (1)	228.67	Chaperone protein DnaK	2869.27	872.74	3.29	
NIRQ_PSEAE	5 (5)	227.06	Denitrification regulatory protein NirQ	9424.06	539.46	17.47	
RS6_PSEA7	6 (3)	222.55	30S ribosomal protein S6	9218.14	472.82	19.50	
RL23_PSEA7	6 (3)	222.36	50S ribosomal protein L23	8886.01	567.27	15.66	
LPXC_PSEAB	4 (3)	221.99	UDP-3-O-acyl-N-acetylglucosamine deacetylase	5291.29	1714.19	3.09	
GLMU_PSEAB	7 (5)	221.43	Bifunctional protein GlmU	1.13E+04	2.70E+04		2.39
F16PA_PSEA7	5 (3)	220.18	Fructose-1,6-bisphosphatase class 1	8668.13	1163.73	7.45	
ARLY_PSEAB	4 (4)	216.67	Argininosuccinate lyase	1963.57	3394.87		1.73
CCPR_PSEAE	5 (5)	214.24	Cytochrome c551 peroxidase	8534.34	607.98	14.04	
DHOM_PSEAE	6 (5)	214.23	Homoserine dehydrogenase	1579.1	2893.05		1.83
SYM_PSEAB	3 (2)	214.06	Methionine--tRNA ligase	2090.07	252.65	8.27	
KPRS_PSEAE	4 (3)	213.02	Ribose-phosphate pyrophosphokinase	4318.36	42.56	101.47	
CHMU_PSEAE	4 (4)	212.86	Monofunctional chorismate mutase	1215.21	3798.56		3.13
RL4_PSEA7	4 (1)	212.69	50S ribosomal protein L4	6925.56	102.42	67.62	
RL15_PSEA7	4 (2)	210.68	50S ribosomal protein L15	4124.69	332.46	12.41	
SYE_PSEAB	5 (2)	210.51	Glutamate--tRNA ligase	669.85	1862.97		2.78
P5CR_PSEAE	5 (5)	208.74	Pyrroline-5-carboxylate reductase	1917.75	209.7	9.15	
ALKD_PSEAE	5 (5)	208.03	2-dehydro-3-deoxy-phosphogluconate aldolase	1.47E+04	1925.18	7.64	
GSHB_PSEAE	5 (3)	207.1	Glutathione synthetase	3198.88	5093.39		1.59
PYRB_PSEAB	5 (3)	205.58	Aspartate carbamoyltransferase	5417.42	2886.34	1.88	
RL24_PSEA7	7 (3)	205.32	50S ribosomal protein L24	1.46E+04	103.14	141.56	
OADC_PSEAB	4 (3)	203.56	Oxaloacetate decarboxylase	1196.42	2466.08		2.06
ATPE_PSEAB	4 (3)	202.98	ATP synthase epsilon chain	2613.41	6176.13		2.36
DBHB_PSEAE	5 (3)	201.83	DNA-binding protein HU-beta	1.90E+04	1628.08	11.67	
NUOB_PSEAB	3 (1)	201.65	NADH-quinone oxidoreductase subunit B	1580.26	15.56	101.56	

HGD_PSEAE	6 (6)	201.51	Homogentisate 1,2-dioxygenase	699.17	2087.85		2.99
GLO2_PSEA8	4 (4)	201.45	Hydroxyacylglutathione hydrolase	3325.61	9252.39		2.78
CYSM_PSEAE	6 (3)	199.57	Cysteine synthase B	4694.64	2035.45	2.31	
UBIC_PSEAE	4 (4)	199.46	Probable chorismate pyruvate-lyase	464.65	802.59		1.73
HSCA_PSEAB	6 (3)	199.31	Chaperone protein HscA homolog	2119.42	240.11	8.83	
Y4395_PSEAE	3 (2)	199.04	UPF0234 protein PA4395	5852.11	314.49	18.61	
PAGL_PSEAE	4 (3)	196.43	Lipid A deacylase PagL	1112.22	2223.03		2.00
UPP_PSEAB	5 (3)	196.06	Uracil phosphoribosyltransferase	1364.21	21.56	63.28	
PYRR_PSEAB	4 (4)	196.02	Bifunctional protein PyrR	6638.94	301.71	22.00	
UVRA_PSEPK	5 (1)	190.1	UvrABC system protein A	2079.55	443.92	4.68	
DNAJ_PSEAB	9 (6)	189.58	Chaperone protein DnaJ	1108.13	962.7	1.15	
DUT_PSEAB	2 (2)	187.92	Deoxyuridine 5'-triphosphate nucleotidohydrolase	885.93	2594.76		2.93
ALGP_PSEAE	4 (3)	180.07	Transcriptional regulatory protein AlgP	3460.67	4305.7		1.24
PNCB2_PSEAE	3 (2)	179.69	Nicotinate phosphoribosyltransferase 2	57.57	581.27		10.10
HFQ_PSEAB	4 (2)	179.57	RNA-binding protein Hfq	6272.4	4.38E+04		6.98
NAPA_PSEAB	5 (2)	179.4	Periplasmic nitrate reductase	38.79	1531.11		39.47
APEB_PSEAB	3 (2)	179.26	Probable M18 family aminopeptidase 2	436.66	1239.79		2.84
FMNRE_PSEAE	4 (4)	177.67	NAD(P)H-dependent FMN reductase PA1204	1157.94	4471.91		3.86
ACEA_PSEAE	5 (4)	174.66	Isocitrate lyase	3426.69	269.08	12.73	
G3P_PSEAE	3 (1)	172.02	Glyceraldehyde-3-phosphate dehydrogenase	7721.55	556.35	13.88	
PDXY_PSEAB	3 (3)	170.72	Pyridoxal kinase PdxY	2925.26	2658.48	1.10	
Y3753_PSEAE	3 (3)	168.5	Uncharacterized protein PA3753	918.25	4390.79		4.78
NFUA_PSEAB	4 (3)	168.33	Fe/S biogenesis protein NfuA	2747.53	4075.29		1.48
DCDA_PSEAE	3 (3)	168.33	Diaminopimelate decarboxylase	430.26	3381.71		7.86
FTSZ_PSEAE	6 (5)	167.25	Cell division protein FtsZ	1400.12	46.79	29.92	
TYSY_PSEAB	4 (3)	166.33	Thymidylate synthase	4311.56	4173.57	1.03	
METZ_PSEAE	4 (3)	165.23	O-succinylhomoserine sulfhydrylase	800.28	2005.68		2.51
PNCB1_PSEAE	3 (1)	165.15	Nicotinate phosphoribosyltransferase	407.89	1227.82		3.01
PDXH_PSEA7	5 (1)	164.61	Pyridoxine/pyridoxamine 5'-phosphate oxidase	8556.53	587.49	14.56	
PUR5_PSEA8	4 (3)	163.41	Phosphoribosylformylglycinamidine cyclo-ligase	2.67E+04	1.36E+04	1.96	

FABA_PSEAB	4 (2)	163.39	3-hydroxydecanoyl-[acyl-carrier-protein] dehydratase	2941.59	3949.1		1.34
ALF_PSEAE	3 (3)	162.17	Fructose-bisphosphate aldolase	2480.29	221.86	11.18	
ACCD_PSEAB	6 (3)	161.08	Acetyl-coenzyme A carboxylase carboxyl transferase subunit beta	6844.21	783.29	8.74	
FDHE_PSEAE	3 (3)	159.76	Protein FdhE homolog	7444.65	736.46	10.11	
TRPB_PSEAE	5 (4)	157.19	Tryptophan synthase beta chain	1226.67	6153.16		5.02
GCH1L_PSEAE	4 (4)	153.56	GTP cyclohydrolase 1 type 2 homolog	2155.87	3086.75		1.43
ARGJ_PSEAE	4 (4)	146.87	Arginine biosynthesis bifunctional protein ArgJ	1.14E+04	2200.54	5.18	
FADB_PSEAB	5 (1)	146.76	Fatty acid oxidation complex subunit alpha	4032.63	132.43	30.45	
BCCP_PSEAE	3 (3)	146.67	Biotin carboxyl carrier protein of acetyl-CoA carboxylase	2.33E+04	1.11E+04	2.10	
ATSE4_PSEAE	4 (4)	146.62	Acetyltransferase PA5475	4369.71	5919.42		1.35
FER_PSEAE	2 (2)	144.86	2Fe-2S ferredoxin	4087.14	162.25	25.19	
COAD_PSEAB	4 (3)	142.89	Phosphopantetheine adenylyltransferase	4113.36	3510.1	1.17	
Y3332_PSEAE	3 (3)	141.81	Uncharacterized PhzA/B-like protein PA3332	37.56	2535.05		67.49
SYT_PSEA7	4 (3)	140.92	Threonine--tRNA ligase	937.19	185.62	5.05	
BETB_PSEAE	3 (2)	139.05	NAD/NADP-dependent betaine aldehyde dehydrogenase	775.85	278.03	2.79	
HSLO_PSEAB	3 (3)	137.45	33 kDa chaperonin	3776.46	857.72	4.40	
ESTA_PSEAE	2 (2)	136.7	Esterase EstA	618.59	1806.23		2.92
GCSH1_PSEAE	3 (3)	135.41	Glycine cleavage system H protein 1	2483.83	7982.1		3.21
PDXJ_PSEA7	3 (1)	134.18	Pyridoxine 5'-phosphate synthase	284.59	584.66		2.05
ASTB_PSEAB	5 (4)	132.75	N-succinylarginine dihydrolase	1737.72	5566.77		3.20
RS14_PSEA7	3 (2)	131.78	30S ribosomal protein S14	4416.98	32.78	134.75	
HSLU_PSEAB	7 (1)	131.09	ATP-dependent protease ATPase subunit HslU	819.86	243.01	3.37	
THRC_PSEAE	5 (2)	130.43	Threonine synthase	572.32	5.61	102.02	
DLDH3_PSEAE	5 (3)	129.96	Dihydrolipoyl dehydrogenase 3	185.38	1944.35		10.49
TPIS_PSEAB	2 (2)	127.71	Triosephosphate isomerase	2016.58	971.26	2.08	
PILH_PSEAE	2 (2)	127.47	Protein PilH	3330.2	91.73	36.30	
PURK_PSEAE	3 (2)	127.06	N5-carboxyaminoimidazole ribonucleotide synthase	1988.81	1191.36	1.67	
RDGC_PSEAB	4 (3)	126.78	Recombination-associated protein RdgC	5110.12	150.05	34.06	
CLPX_PSEAB	3 (1)	126.46	ATP-dependent Clp protease ATP-binding subunit ClpX	1755.7	35.23	49.84	
RNPA_PSEA7	3 (2)	125.35	Ribonuclease P protein component	572.23	706.31		1.23

RL20_PSEA7	2 (2)	124.51	50S ribosomal protein L20	4549.15	149.21	30.49	
HEM3_PSEA8	3 (3)	123.98	Porphobilinogen deaminase	703.1	85.94	8.18	
HIS7_PSEA7	3 (3)	122.74	Imidazoleglycerol-phosphate dehydratase	1699.09	1411.35	1.20	
PYRH_PSEAB	4 (2)	121.52	Uridylate kinase	5858.33	2369.29	2.47	
ATPD_PSEAB	2 (1)	121.01	ATP synthase subunit delta	5651.24	605.1	9.34	
RS3_PSEA7	3 (3)	120.71	30S ribosomal protein S3	5550.87	386.98	14.34	
SKPL_PSEAE	2 (1)	119.62	Skp-like protein	7201.45	3958.74	1.82	
RNPH_PSEAB	3 (2)	119.36	Ribonuclease PH	828.99	2737.87		3.30
RL16_PSEA7	3 (3)	118.83	50S ribosomal protein L16	2088.51	282.23	7.40	
RS9_PSEA7	2 (2)	118.71	30S ribosomal protein S9	7772.2	51.57	150.71	
MQO2_PSEAE	4 (4)	117.72	Probable malate:quinone oxidoreductase 2	1652.22	35.11	47.06	
UGND_PSEAE	2 (2)	116.89	UDP-N-acetyl-D-glucosamine 6-dehydrogenase	1824.62	684.39	2.67	
FLIA_PSEAE	5 (2)	116.55	RNA polymerase sigma factor FliA	1391.88	83.9	16.59	
PLPHP_PSEAE	4 (2)	116.3	Pyridoxal phosphate homeostasis protein	740.64	3368.32		4.55
RBFA_PSEAB	1 (1)	113.78	Ribosome-binding factor A	277.96	881.27		3.17
ACP1_PSEAE	2 (1)	113.52	Acyl carrier protein 1	3.51E+04	9.06E+04		2.58
FER1_PSEAE	2 (2)	112.77	Ferredoxin 1	2999.61	3890.47		1.30
PTA_PSEAE	6 (1)	112.74	Phosphate acetyltransferase	20.38	5532.15		271.45
TRMB_PSEAB	3 (2)	112.41	tRNA (guanine-N(7)-)-methyltransferase	2435.77	118.74	20.51	
ELAS_PSEAB	2 (2)	112.2	Elastase	657.19	917.64		1.40
OTCA_PSEAE	7 (5)	111.83	Ornithine carbamoyltransferase, anabolic	1.31E+04	1.01E+05		7.71
PDXA_PSEAE	2 (2)	111.7	4-hydroxythreonine-4-phosphate dehydrogenase	795.7	1318.83		1.66
MSRA_PSEAB	3 (3)	109.5	Peptide methionine sulfoxide reductase MsrA	1659.92	3663.09		2.21
ERPA_PSEAB	3 (3)	107.76	Iron-sulfur cluster insertion protein ErpA	1993.48	477.38	4.18	
RS13_PSEA7	2 (2)	106.36	30S ribosomal protein S13	8078.48	19.29	418.79	
THTR_PSEAE	2 (1)	104.77	Thiosulfate sulfurtransferase	1890.62	1308.38	1.45	
LGUL_PSEAE	2 (2)	103.15	Lactoylglutathione lyase	1322.72	3549.79		2.68
AROC_PSEAB	2 (2)	102.53	Chorismate synthase	33.43	1295.61		38.76
Y2770_PSEAE	3 (2)	101.27	Uncharacterized isomerase PA2770	7847.34	2.86E+04		3.64
ALR2_PSEAE	4 (2)	97.72	Alanine racemase, catabolic	2555.9	3655.8		1.43

QOR_PSEAE	2 (1)	96.3	Quinone oxidoreductase	308.94	51.32	6.02	
SYFB_PSEAE	3 (2)	95.85	Phenylalanine--tRNA ligase beta subunit	1069.12	23.95	44.64	
HCP1_PSEAE	2 (2)	95.55	Protein hcp1	1.35E+04	491.94	27.44	
AROB_PSEAB	3 (1)	93.69	3-dehydroquinate synthase	807.18	812.2		1.01
Y329_PSEAE	2 (2)	93.19	UPF0339 protein PA0329	3762.47	1884.88	2.00	
ASTD_PSEAE	2 (2)	91.56	N-succinylglutamate 5-semialdehyde dehydrogenase	627.2	12.16	51.58	
RS20_PSEA7	1 (1)	91.16	30S ribosomal protein S20	1.85E+04	6527.01	2.83	
PUR7_PSEA7	4 (1)	90.85	Phosphoribosylaminoimidazole-succinocarboxamide synthase	1968.6	1.05E+04		5.33
AROQ1_PSEAE	2 (1)	89.87	3-dehydroquinate dehydratase 1	1767.22	6600.29		3.73
CAPP_PSEAE	5 (5)	89.45	Phosphoenolpyruvate carboxylase	1858.88	1442.91	1.29	
PYS1_PSEAI	3 (1)	89.41	Pyocin-S1	1982.89	302.83	6.55	
PUR1_PSEAE	3 (3)	89.14	Amidophosphoribosyltransferase	514.49	35.44	14.52	
ASSY_PSEAB	4 (2)	87.93	Argininosuccinate synthase	1471.97	26.29	55.99	
CHEZ_PSEAB	4 (4)	83.19	Protein phosphatase CheZ	3622.56	244.4	14.82	
HIS4_PSEA7	3 (3)	83.18	1-(5-phosphoribosyl)-5-[(5-phosphoribosylamino)methylideneamino] imidazole-4-carboxamide isomerase	3595.37	295.57	12.16	
PMPR_PSEAE	3 (2)	82.44	Transcriptional regulatory protein PmpR	9713.42	557.21	17.43	
RL17_PSEA7	4 (3)	79.89	50S ribosomal protein L17	4962.1	239.24	20.74	
PTSN_PSEAE	2 (2)	79.88	Nitrogen regulatory protein	1279.24	254.81	5.02	
NIRC_PSEAE	1 (1)	78.24	Cytochrome c55X	454.71	2994.13		6.58
ECOT_PSEAB	4 (3)	78.2	Ecotin	2883.57	337.12	8.55	
HEMN_PSEAE	2 (1)	77.13	Oxygen-independent coproporphyrinogen III oxidase	546.39	25.59	21.35	
SYP_PSEAB	3 (3)	77.02	Proline--tRNA ligase	1167.02	1177.59		1.01
ALGR_PSEAE	3 (2)	76.95	Positive alginate biosynthesis regulatory protein	1922.49	36.2	53.11	
LDC_PSEAE	1 (1)	76.81	Murein tetrapeptide carboxypeptidase	785.13	5582.07		7.11
Y4465_PSEAE	4 (4)	76.55	Nucleotide-binding protein PA4465	954.73	450.85	2.12	
MURC_PSEAB	2 (2)	76.49	UDP-N-acetylmuramate--L-alanine ligase	198.42	37.37	5.31	
AROB_PSEA7	3 (1)	76.39	3-dehydroquinate synthase	1383.22	1864.43		1.35
NDPA_PSEAB	3 (3)	74.33	Nucleoid-associated protein PA14_59050	1.12E+04	4171.22	2.69	
RSMH_PSEAB	2 (2)	74.04	Ribosomal RNA small subunit methyltransferase H	2632.02	6090.72		2.31

ASTE_PSEAB	2 (2)	73.64	Succinylglutamate desuccinylase	547.73	911.31		1.66
OPRI_PSEAE	1 (1)	73.23	Major outer membrane lipoprotein	593.53	1177.49		1.98
HEMTB_PSEAB	2 (1)	73.2	Bacteriohemerythrin	222.85	277.57		1.25
AMIC_PSEAE	1 (1)	71.97	Aliphatic amidase expression-regulating protein	154.2	811.66		5.26
ALR1_PSEAE	2 (2)	71.6	Alanine racemase, biosynthetic	391	818.68		2.09
CATE_PSEAE	2 (1)	71.02	Catalase HPII	0	744.58		∞
LASA_PSEAE	2 (1)	69.96	Protease LasA	696.03	718.52		1.03
TRPA_PSEAB	3 (3)	69.72	Tryptophan synthase alpha chain	2059.41	427.76	4.81	
IXTPA_PSEAE	2 (2)	68.67	dITP/XTP pyrophosphatase	1111.43	29.87	37.21	
LTAE_PSEAE	2 (2)	67.59	Low specificity L-threonine aldolase	4618.48	396.86	11.64	
APAG_PSEAB	1 (1)	67.57	Protein ApaG	253.97	429.54		1.69
KGUA_PSEAE	1 (1)	66.64	Guanylate kinase	2277.73	391.36	5.82	
Y4473_PSEAE	2 (2)	66.59	UPF0307 protein PA4473	1061.95	269.02	3.95	
MRAZ_PSEA8	2 (1)	66.13	Transcriptional regulator MraZ	254.03	4263.43		16.78
PUR2_PSEAE	2 (1)	65.08	Phosphoribosylamine--glycine ligase	1943.18	471.82	4.12	
NQOR_PSEAE	1 (1)	64.3	NAD(P)H dehydrogenase (quinone)	634.11	258.06	2.46	
TRPG_PSEAE	1 (1)	64	Anthranilate synthase component 2	1026.84	116.99	8.78	
RPIA_PSEAB	2 (2)	63.61	Ribose-5-phosphate isomerase A	1813.36	8.45	214.60	
RSMG_PSEA7	2 (2)	63.49	Ribosomal RNA small subunit methyltransferase G	765.92	556.46	1.38	
Y2077_PSEAB	1 (1)	63.12	STAS-domain containing protein PA14_20770	76.5	1399.64		18.30
SYFA_PSEAB	2 (2)	61.17	Phenylalanine--tRNA ligase alpha subunit	214.5	24.69	8.69	
AMIE_PSEAB	3 (1)	60.57	Aliphatic amidase	408.8	63.93	6.39	
PURT_PSEAB	5 (2)	59.72	Formate-dependent phosphoribosylglycinamide formyltransferase	606.52	49.55	12.24	
HIS3_PSEA7	1 (1)	59.71	Phosphoribosyl-AMP cyclohydrolase	1819.37	4736.25		2.60
RS17_PSEA7	1 (1)	59.19	30S ribosomal protein S17	5020.08	1528.56	3.28	
GCSH2_PSEAE	2 (1)	59.12	Glycine cleavage system H protein 2	1431.52	4090.73		2.86
ARNB_PSEAB	3 (1)	58.94	UDP-4-amino-4-deoxy-L-arabinose--oxoglutarate aminotransferase	70.78	1354.06		19.13
IVY_PSEAE	1 (1)	58.69	Inhibitor of vertebrate lysozyme	558.75	3890.5		6.96
PHEA_PSEAE	1 (1)	58.21	P-protein	1036.89	506.92	2.05	
COBW_PSEAE	2 (1)	57.18	Protein CobW	958.69	57.57	16.65	

RS18_PSEA7	1 (1)	56.52	30S ribosomal protein S18	898.85	0	∞	
NQRF_PSEAB	2 (2)	56.13	Na(+)-translocating NADH-quinone reductase subunit F	14.13	1829.7		129.49
YGFB_PSEAE	1 (1)	55.74	UPF0149 protein PA5225	1686.3	386.62	4.36	
FOLD_PSEAB	1 (1)	55.73	Bifunctional protein FolD	5147.58	3707.66	1.39	
RL31_PSEA7	1 (1)	55.57	50S ribosomal protein L31	7196.89	126.34	56.96	
QUEA_PSEAB	3 (2)	54.59	S-adenosylmethionine:tRNA ribosyltransferase-isomerase	253.26	635.55		2.51
LASA_PSEAB	1 (1)	53.96	Protease LasA	699.26	1475.58		2.11
CHEB1_PSEAE	3 (1)	53.46	Chemotaxis response regulator protein-glutamate methylesterase of group 1 operon	741.33	2.16	343.21	
GBUA_PSEAE	1 (1)	52.37	Guanidinobutyrase	804.37	1517.39		1.89
CYC5_PSEME	1 (1)	51.93	Cytochrome c5	1519.49	2966.64		1.95
RMLC_PSEAE	2 (2)	50.79	dTDP-4-dehydrorhamnose 3,5-epimerase	618.57	513.48	1.20	
KYNB_PSEAB	1 (1)	49.67	Kynurenine formamidase	2420.57	4093.52		1.69
SYT_PSEAB	3 (1)	48.07	Threonine--tRNA ligase	0	225.17		∞
MOAC_PSEAB	1 (1)	47.72	Cyclic pyranopterin monophosphate synthase	1626.22	497.26	3.27	
NADC_PSEAE	2 (2)	46.2	Nicotinate-nucleotide pyrophosphorylase [carboxylating]	1035.79	30.04	34.48	
FABV_PSEAB	2 (1)	46.19	Enoyl-[acyl-carrier-protein] reductase [NADH]	855.32	59.28	14.43	
DCUP_PSEA7	2 (2)	44.77	Uroporphyrinogen decarboxylase	306.4	92.32	3.32	
COAX_PSEAB	2 (1)	43.78	Type III pantothenate kinase	1428.43	77.53	18.42	
GLMM_PSEA7	3 (1)	42.24	Phosphoglucosamine mutase	516.08	132.5	3.89	
RF2_PSEAE	1 (1)	42.22	Peptide chain release factor 2	351.15	9.24	38.00	
CATB_PSEAE	1 (1)	41.77	Catalase	0	1034.29		∞
Y2116_PSEAE	1 (1)	41.66	Putative hydro-lyase PA2116	2164.19	1109.28	1.95	
MTNA_PSEAB	1 (1)	41.2	Methylthioribose-1-phosphate isomerase	578.77	383.65	1.51	
RL30_PSEA7	1 (1)	40.6	50S ribosomal protein L30	568.91	2	284.46	
MUPP_PSEAE	1 (1)	39.33	N-acetylmuramic acid 6-phosphate phosphatase	145.67	23.31	6.25	
GCH11_PSEAE	1 (1)	38.98	GTP cyclohydrolase 1 1	1266.16	3092.33		2.44
CARA_PSEAE	1 (1)	38.94	Carbamoyl-phosphate synthase small chain	450.26	0	∞	
PORB_PSEAE	2 (1)	38.92	Porin B	104.61	585.09		5.59
ASTA_PSEAE	1 (1)	37.96	Arginine N-succinyltransferase subunit alpha	236.83	0	∞	
RL32_PSEA7	1 (1)	37.96	50S ribosomal protein L32	6017.55	109.37	55.02	

CHEY_PSEAE	1 (1)	37.8	Chemotaxis protein CheY	2439.48	3156.78		1.29
RPSH_PSEAE	1 (1)	36.76	RNA polymerase sigma-H factor	0	331.06		∞
MINE_PSEAB	1 (1)	33.94	Cell division topological specificity factor	264.42	6.05	43.71	
6PGL_PSEAE	1 (1)	33.85	6-phosphogluconolactonase	142.16	815.64		5.74
Y2112_PSEAE	1 (1)	33.27	UPF0271 protein PA2112	698.63	67.07	10.42	
Y3435_PSEAE	2 (1)	32.79	Uncharacterized protein PA3435	491.17	409.24	1.20	

Supplementary Table S3. Cell-free secretome of *P. aeruginosa* CF isolate 5 following growth under normoxia and anoxia. Data represents the average normalised abundances and fold increase from *N*=1. A fold change of 1.0 is indicative of no change between normoxia and anoxia. I5A = *P. aeruginosa* CF isolate 5 grown under anoxia. I5O = *P. aeruginosa* CF isolate 5 grown under normoxia.

Accession	Peptides (unique)	Score	Description	Average Normalised Abundances		Fold increase	
				I5A	I5O	I5A	I5O
FLICA_PSEAI	22 (19)	1347.86	A-type flagellin	5.51E+05	2.81E+05	1.96	
ELAS_PSEAE	18 (16)	1157.47	Elastase	1.04E+06	3.42E+05	3.04	
CH60_PSEA7	21 (5)	910.31	60 kDa chaperonin	1.44E+04	2.94E+04		2.04
ASPQ_PSEAE	15 (15)	907.55	Glutaminase-asparaginase	8691.33	2.72E+04		3.13
LAP_PSEAB	13 (11)	900.05	Aminopeptidase	5.03E+05	1.34E+05	3.75	
PORF_PSEAE	10 (10)	884.59	Outer membrane porin F	1.39E+05	4.98E+04	2.79	
P5217_PSEAE	13 (11)	821.83	Probable binding protein component of ABC iron transporter PA521	1592.75	2.12E+04		13.31
DSBA_PSEAB	14 (10)	753.24	Thiol:disulfide interchange protein DsbA	2.33E+04	9.48E+04		4.07
ATPA_PSEAB	13 (2)	740.06	ATP synthase subunit alpha	4351.48	1.90E+04		4.37
DAPA_PSEAE	11 (11)	696.03	4-hydroxy-tetrahydrodipicolinate synthase	2854.76	1.36E+04		4.76
THIO_PSEAE	12 (11)	644.87	Thioredoxin	2.14E+04	6.85E+04		3.20
CBPD_PSEAB	13 (12)	604.43	Chitin-binding protein CbpD	1.70E+06	6.08E+05	2.80	
NIRS_PSEAE	10 (9)	601.5	Nitrite reductase	1.53E+04	1.47E+04	1.04	

ATPA_PSEMY	12 (1)	585.79	ATP synthase subunit alpha	1216.18	1241.84		1.02
LYSC_PSEAB	11 (11)	579.89	Lysyl endopeptidase	3.74E+05	1.75E+05	2.14	
Y1579_PSEAE	9 (8)	577.11	Uncharacterized protein PA1579	4037.59	4.55E+04		11.27
Y421_PSEA8	9 (9)	573.69	UPF0312 protein PLES_04211	8709.97	3.33E+04		3.82
SAHH_PSEAB	12 (6)	542.94	Adenosylhomocysteinase	467.69	5341.65		11.42
BRAC_PSEAE	8 (7)	533.55	Leucine-, isoleucine-, valine-, threonine-, and alanine-binding protein	3.59E+04	1.08E+05		3.01
EFG1_PSEAE	9 (7)	485.44	Elongation factor G 1	1.35E+04	2.48E+04		1.84
FLID1_PSEAI	9 (8)	464.38	A-type flagellar hook-associated protein 2	3.01E+04	3.23E+04		1.07
AHPC_PSEAB	7 (7)	464.05	Alkyl hydroperoxide reductase C	2.68E+04	1.61E+05		6.01
ATOB_PSEAE	11 (11)	425.58	Acetyl-CoA acetyltransferase	4925.97	3.25E+04		6.60
APRA_PSEAE	8 (8)	421.89	Serralysin	2.45E+05	3.21E+04	7.63	
ARUC_PSEAE	7 (7)	418.12	Succinylornithine transaminase/acetylornithine aminotransferase	5284.31	2.41E+04		4.56
METK_PSEAB	10 (4)	413.48	S-adenosylmethionine synthase	1114.67	5048.93		4.53
ODP2_PSEAE	9 (7)	389.77	Dihydrolipoyllysine-residue acetyltransferase component of pyruvate dehydrogenase complex	2119.73	7432.06		3.51
GGT_PSEAE	7 (6)	389.69	Glutathione hydrolase proenzyme	1.23E+04	9250.34	1.33	
LOLA_PSEAB	7 (7)	375.56	Outer-membrane lipoprotein carrier protein	2161.36	6725.16		3.11
Y3922_PSEAE	7 (6)	369.63	Uncharacterized protein PA3922	5920.01	3.33E+04		5.62
DLDH1_PSEAE	9 (8)	366.92	Dihydrolipoyl dehydrogenase	3763.46	1.47E+04		3.91
IPYR_PSEAE	5 (5)	366.06	Inorganic pyrophosphatase	4847.01	2.83E+04		5.84
Y329_PSEAE	4 (4)	362.05	UPF0339 protein PA0329	4155.45	1.51E+04		3.63
LASA_PSEAB	6 (6)	357.97	Protease LasA	3.09E+05	1.04E+05	2.97	
SPUD_PSEAB	9 (7)	354.08	Putrescine-binding periplasmic protein SpuD	9906.37	4.24E+04		4.28
ATPB_PSEAB	7 (3)	351.44	ATP synthase subunit beta	772.97	1.18E+04		15.27
OTCC_PSEAE	8 (1)	349.8	Ornithine carbamoyltransferase, catabolic	1229.86	2997.71		2.44
SSB_PSEAE	5 (4)	334.23	Single-stranded DNA-binding protein	5027.51	1.74E+04		3.46
HUTU_PSEA7	6 (1)	329.11	Urocanate hydratase	1554.81	1518.81	1.02	
CATA_PSEAE	7 (4)	315.14	Catalase	6818.79	3867.39	1.76	
FUMC1_PSEAE	6 (6)	297.18	Fumarate hydratase class II 1	1736.54	6845.89		3.94
CLPB_PSEAE	7 (7)	278.97	Chaperone protein ClpB	2344.11	6898.33		2.94

OPRM_PSEAE	6 (4)	277.92	Outer membrane protein OprM	3254.26	341.25	9.54	
PHZB1_PSEAE	5 (5)	267.98	Phenazine biosynthesis protein PhzB 1	723.94	1.52E+04		21.00
PAL_PSEAE	5 (5)	260.61	Peptidoglycan-associated lipoprotein	3542.04	2.09E+04		5.90
AZUR_PSEAE	4 (3)	257.59	Azurin OS=Pseudomonas aeruginosa	2.47E+04	2.61E+04		1.06
PYRC_PSEAB	5 (4)	251.23	Dihydroorotase	3.32E+04	8389.97	3.96	
ETFB_PSEAE	3 (3)	249.15	Electron transfer flavoprotein subunit beta	6210.64	1903.72	3.26	
Y3753_PSEAE	3 (3)	248.08	Uncharacterized protein PA3753	5096.51	1.89E+04		3.71
HEM2_PSEAE	5 (4)	230.2	Delta-aminolevulinic acid dehydratase	4042.07	9080.51		2.25
PGK_PSEA8	8 (4)	229.71	Phosphoglycerate kinase	4417.29	9406.84		2.13
ILVC_PSEA7	5 (5)	217.23	Ketol-acid reductoisomerase (NADP(+))	3571.21	1.04E+04		2.91
T23O_PSEAB	3 (3)	217.07	Tryptophan 2,3-dioxygenase	293.74	3114.94		10.60
PROA_PSEAB	4 (3)	217.02	Gamma-glutamyl phosphate reductase	1955.91	7011.27		3.58
ILVE_PSEAE	5 (5)	213.7	Branched-chain-amino-acid aminotransferase	1837.86	6827.27		3.71
ISPF_PSEAE	3 (1)	211.77	2-C-methyl-D-erythritol 2,4-cyclodiphosphate synthase	409.27	5431.25		13.27
SODF_PSEAE	4 (4)	201.12	Superoxide dismutase [Fe]	1.28E+05	3.38E+05		2.64
HFQ_PSEAB	5 (5)	200.96	RNA-binding protein Hfq	1847.79	7761.8		4.20
CISY_PSEAE	9 (4)	200.13	Citrate synthase	605.12	1.21E+04		20.00
SPUE_PSEAE	5 (5)	198.78	Spermidine-binding periplasmic protein SpuE	1645.1	8256.11		5.02
IDH_PSEAB	4 (2)	198.41	Isocitrate dehydrogenase [NADP]	4648.36	1624.41	2.86	
MASZ_PSEAB	4 (1)	193.94	Malate synthase G	180.4	860.79		4.77
PURE_PSEAE	3 (3)	191.09	N5-carboxyaminoimidazole ribonucleotide mutase	2587.38	1.38E+04		5.33
DAVD_PSEAE	5 (3)	182.43	Glutarate-semialdehyde dehydrogenase DavD	1.70E+05	8.36E+04	2.03	
PCKA_PSEAE	5 (3)	181.45	Phosphoenolpyruvate carboxykinase (ATP)	2036.14	4633.38		2.28
PHS_PSEAB	4 (4)	179.58	Putative pterin-4-alpha-carbinolamine dehydratase	2462.79	4.26E+04		17.30
CYSNC_PSEAE	4 (3)	179.04	Bifunctional enzyme CysN/CysC	130.42	1690.25		12.96
FABZ_PSEAB	4 (4)	170.44	3-hydroxyacyl-[acyl-carrier-protein] dehydratase FabZ	856.02	4223.85		4.93
FABI_PSEAE	4 (2)	169.27	Enoyl-[acyl-carrier-protein] reductase [NADH] FabI	82.9	1734.03		20.92
NOSZ_PSEAE	4 (3)	165.98	Nitrous-oxide reductase	4180.25	2374.03	1.76	
TOP1_PSEAE	6 (5)	164.26	DNA topoisomerase 1	5599.09	1885.04	2.97	
ATPG_PSEAB	4 (1)	161.55	ATP synthase gamma chain	397	3387.07		8.53

CHMU_PSEAE	3 (3)	160.58	Monofunctional chorismate mutase	1621.12	4942.35		3.05
DAVD_PSEPK	4 (1)	155.83	Glutarate-semialdehyde dehydrogenase DavD	5.73E+04	4.62E+04	1.24	
EFTU_PSEAB	3 (2)	154.92	Elongation factor Tu	4963.33	3391.32	1.46	
ALKD_PSEAE	3 (3)	154.76	2-dehydro-3-deoxy-phosphogluconate aldolase	803.17	2947.19		3.70
OPRI_PSEAE	4 (4)	154.63	Major outer membrane lipoprotein	6728.66	4601.61	1.46	
FMK1_PSEAI	3 (3)	154.06	Fimbrial protein	1267.1	4194.9		3.31
LGUL_PSEAE	3 (2)	153.71	Lactoylglutathione lyase	148.09	4026.71		27.19
ZIPA_PSEAB	4 (4)	152.21	Cell division protein ZipA	1941.07	1.37E+04		7.06
FUR_PSEAE	2 (2)	150.63	Ferric uptake regulation protein	1252.99	2832.98		2.26
NDK_PSEAB	4 (4)	148.22	Nucleoside diphosphate kinase	5712.11	3.15E+04		5.51
ODB2_PSEAE	3 (3)	142.98	Lipoamide acyltransferase component of branched-chain alpha-keto acid dehydrogenase complex	1619.44	2229.45		1.38
SODM_PSEAE	2 (2)	140.02	Superoxide dismutase [Mn]	391.45	4.41E+04		112.66
CY551_PSEAE	2 (2)	134.69	Cytochrome c-551	1.25E+04	9647.96	1.30	
DAVT_PSEAE	6 (6)	133.76	5-aminovalerate aminotransferase DavT	1.51E+04	1.47E+04	1.03	
PORD_PSEAE	5 (5)	132.18	Porin D	4.80E+04	1.58E+04	3.04	
FLICB_PSEAE	5 (2)	130.89	B-type flagellin	1.30E+04	1.05E+05		8.08
BFR_PSEAE	3 (3)	130.5	Bacterioferritin	8965.38	3995.26	2.24	
MUCB_PSEAE	2 (2)	127.64	Sigma factor AlgU regulatory protein MucB	1855.62	8953.2		4.82
AROQ1_PSEAE	1 (1)	127.17	3-dehydroquinate dehydratase 1	804.74	4969.31		6.18
DNAK_PSEAB	6 (2)	126.73	Chaperone protein DnaK	2.19E+04	7141.13	3.07	
HPPD_PSEAE	2 (2)	125.88	4-hydroxyphenylpyruvate dioxygenase	398.77	1338.11		3.36
FER1_PSEAE	2 (2)	123.24	Ferredoxin 1	2888.54	1.20E+04		4.15
ACKA_PSEA7	4 (2)	122.83	Acetate kinase	1338.28	2495.67		1.86
EDD_PSEAE	3 (2)	122.63	Phosphogluconate dehydratase	592.6	2153.26		3.63
BCCP_PSEAE	2 (2)	119.46	Biotin carboxyl carrier protein of acetyl-CoA carboxylase	3044.5	1.31E+04		4.30
AMPA_PSEAB	3 (2)	117.54	Probable cytosol aminopeptidase	1067.14	5033.73		4.71
GCH12_PSEAE	3 (2)	113.24	GTP cyclohydrolase 1 2	464.43	1245.87		2.68
AGUA_PSEAE	2 (2)	110.49	Agmatine deiminase	622.91	2261.71		3.63
ETFA_PSEAE	2 (2)	106.09	Electron transfer flavoprotein subunit alpha	3467.17	401.17	8.64	

CLPP2_PSEAE	2 (2)	105.1	ATP-dependent Clp protease proteolytic subunit 2	2262.35	1539.82	1.47	
HEM6_PSEA7	3 (3)	103.42	Oxygen-dependent coproporphyrinogen-III oxidase	2282.85	2433.51		1.066
MMSA_PSEAE	2 (2)	102.9	Methylmalonate-semialdehyde dehydrogenase [acylating]	1.72E+05	2.16E+04	7.96	
HSLV_PSEAB	1 (1)	100.21	ATP-dependent protease subunit HslV	2514.41	9459.8		3.76
ACP1_PSEAE	2 (2)	99.43	Acyl carrier protein 1	4308.68	9679.52		2.25
PSTS_PSEAB	4 (3)	99	Phosphate-binding protein PstS	876.99	9024.94		10.29
FABB_PSEAB	2 (2)	94.36	3-oxoacyl-[acyl-carrier-protein] synthase 1	1862.16	8069.35		4.33
ETFD_PSEAE	4 (4)	91.86	Electron transfer flavoprotein-ubiquinone oxidoreductase	3478.9	2440.57	1.43	
FUMC2_PSEAE	5 (4)	86.67	Fumarate hydratase class II 2	1.08E+04	3822.43	2.83	
PAGL_PSEAE	1 (1)	78.49	Lipid A deacylase PagL	2232.44	36.21	61.65	
DNAK_PSEA7	5 (1)	77.96	Chaperone protein DnaK	5.45E+05	1.62E+05	3.36	
RMLC_PSEAE	1 (1)	76.07	dTDP-4-dehydrorhamnose 3,5-epimerase	0	2736.76		∞
CYSM_PSEAE	2 (1)	75.21	Cysteine synthase B	373.1	5489.46		14.71
LDC_PSEAE	1 (1)	74.59	Murein tetrapeptide carboxypeptidase	0	4672.8		∞
SUCD_PSEAE	4 (3)	73.23	Succinate--CoA ligase [ADP-forming] subunit alpha	683.54	1742.38		2.55
PANB2_PSEAB	1 (1)	72.63	3-methyl-2-oxobutanoate hydroxymethyltransferase 2	1752.18	9107.42		5.20
UVRA_PSEAE	4 (4)	71.27	UvrABC system protein A	1428.4	1431.88		1.00
DCTP_PSEAE	1 (1)	68.96	C4-dicarboxylate-binding periplasmic protein DctP	1157.27	2254.23		1.95
CYCH_PSEAE	1 (1)	61.52	Cytochrome c-type biogenesis protein CycH	16.73	1320.44		78.93
FMNRE_PSEAE	1 (1)	57.93	NAD(P)H-dependent FMN reductase PA1204	340.12	2925.31		8.60
HIS1_PSEA8	1 (1)	57.74	ATP phosphoribosyltransferase	1390.83	4427.97		3.18
GCSH1_PSEAE	1 (1)	57.14	Glycine cleavage system H protein 1	1502.36	3778.67		2.51
NUOCD_PSEAB	1 (1)	52.41	NADH-quinone oxidoreductase subunit C/D	835.07	3711.1		4.44
GMHA_PSEAB	1 (1)	47.44	Phosphoheptose isomerase	3577.56	1.22E+04		3.41
PILY1_PSEAE	1 (1)	46.66	Type IV pilus biogenesis factor PilY1	2841.63	1321.2	2.15	
PLPHP_PSEAE	2 (1)	46	Pyridoxal phosphate homeostasis protein	65.41	1033.07		15.79
RISB_PSEAB	2 (2)	39.47	6,7-dimethyl-8-ribityllumazine synthase	775.56	4642.1		5.98
NADE_PSEAB	1 (1)	37.97	NH(3)-dependent NAD(+) synthetase	2064.82	6243.17		3.02
TPX_PSEAE	1 (1)	37.87	Thiol peroxidase	3501.18	768.79	4.55	
AHPF_PSEAE	2 (1)	37.39	Alkyl hydroperoxide reductase subunit F	87.03	2110.56		24.25

HGD_PSEAE	1 (1)	36.11	Homogentisate 1,2-dioxygenase	371.9	2289.1		6.15
GCH1L_PSEAE	1 (1)	33.98	GTP cyclohydrolase 1 type 2 homolog	752.81	4290.5		5.70
GCSH2_PSEAE	1 (1)	33.6	Glycine cleavage system H protein 2	1286.84	1214.81	1.06	
PPIA_PSEAE	1 (1)	30.93	Peptidyl-prolyl cis-trans isomerase A	5343.06	7055.94		1.32

Supplementary Table S4. Cell-free secretome of *P. aeruginosa* CF isolate 6 following growth under normoxia and anoxia. Data represents the average normalised abundances and fold increase from *N*=1. A fold change of 1.0 is indicative of no change between normoxia and anoxia.. I6A = *P. aeruginosa* CF isolate 6 grown under anoxia. I6O = *P. aeruginosa* CF isolate 6 grown under normoxia.

Accession	Peptides (unique)	Score	Description	Average Normalised Abundances		Fold increase	
				I6A	I6O	I6A	I6O
NIRS_PSEAE	46 (41)	3926.07	Nitrite reductase	3.51E+05	3.95E+05		1.13
DNAK_PSEAB	41 (7)	3051.93	Chaperone protein DnaK	9.38E+04	8.92E+04	1.05	
CLPB_PSEAE	44 (21)	2778.56	Chaperone protein ClpB	2.42E+04	2.30E+04	1.05	
EFG1_PSEAE	48 (12)	2717.96	Elongation factor G 1	9.14E+04	1.16E+05		1.27
DHE2_PSEAE	42 (30)	2091.59	NAD-specific glutamate dehydrogenase	4.15E+04	1.63E+04	2.55	
NOSZ_PSEAE	32 (15)	1982.41	Nitrous-oxide reductase	1.13E+05	1.34E+05		1.19
Y421_PSEA8	22 (6)	1977.61	UPF0312 protein PLES_04211	8.32E+04	5.48E+04	1.52	
ACNB_PSEAE	31 (23)	1839.54	Aconitate hydratase B	8.79E+04	2.45E+04	3.59	
RPOB_PSEAB	40 (8)	1820.31	DNA-directed RNA polymerase subunit beta	1.63E+04	8852.14	1.84	
IDH_PSEAB	27 (19)	1646.45	Isocitrate dehydrogenase [NADP]	8.57E+04	7.81E+04	1.10	
HTPG_PSEAE	27 (6)	1634.94	Chaperone protein HtpG	2.83E+04	4.37E+04		1.54
RPOC_PSEAB	33 (7)	1554.88	DNA-directed RNA polymerase subunit beta'	1.26E+04	9488.99	1.33	
PNP_PSEAB	26 (8)	1465.56	Polyribonucleotide nucleotidyltransferase	1.79E+04	1.30E+04	1.38	
HPPD_PSEAE	21 (17)	1463.22	4-hydroxyphenylpyruvate dioxygenase	1.75E+04	1.06E+05		6.06
DAVT_PSEAE	21 (12)	1455.1	5-aminovalerate aminotransferase DavT	9.07E+04	1.17E+05		1.29
DAVD_PSEAE	24 (16)	1448.46	Glutarate-semialdehyde dehydrogenase DavD	5.34E+04	5.17E+04	1.03	

FLICA_PSEAI	18 (16)	1445.7	A-type flagellin	4.50E+04	3.21E+04	1.40	
DLDH1_PSEAE	24 (18)	1346.67	Dihydrolipoyl dehydrogenase	2.52E+04	6.22E+04		2.47
EFTS_PSEAB	20 (8)	1318.89	Elongation factor Ts	3.90E+04	4.58E+04		1.17
CATA_PSEAE	22 (17)	1293.81	Catalase	5.87E+04	8.90E+04		1.52
EFTU_PSEAB	20 (5)	1274.31	Elongation factor Tu	9.26E+04	7.84E+04	1.18	
ACNA_PSEAE	23 (16)	1244.52	Aconitate hydratase A	5.14E+04	3.15E+04	1.63	
CH60_PSEA7	25 (2)	1228.89	60 kDa chaperonin	7185.77	2535.63	2.83	
Y3922_PSEAE	21 (17)	1223.59	Uncharacterized protein PA3922	4.95E+04	5.87E+04		1.19
DSBA_PSEAB	16 (15)	1191.11	Thiol:disulfide interchange protein DsbA	1.15E+05	7.89E+04	1.46	
RRF_PSEAB	16 (14)	1130.73	Ribosome-recycling factor	4.83E+04	8.35E+04		1.73
ATPB_PSEAB	20 (6)	1116.71	ATP synthase subunit beta	2744.58	3756.19		1.37
SYL_PSEAB	19 (10)	1100.15	Leucine--tRNA ligase	8177.06	9805.77		1.20
FADB_PSEAB	23 (9)	1092.52	Fatty acid oxidation complex subunit alpha	1.71E+04	1.29E+04	1.33	
ATPA_PSEAB	18 (2)	1091.07	ATP synthase subunit alpha	1.71E+04	2.33E+04		1.36
EFG2_PSEAE	19 (2)	1084.94	Elongation factor G 2	1197.54	2236.07		1.87
MASZ_PSEAB	18 (8)	1065.06	Malate synthase G	2.95E+04	4.66E+04		1.58
FABY_PSEAE	16 (14)	1064.02	Beta-ketoacyl-[acyl-carrier-protein] synthase FabY	1.05E+04	2.25E+04		2.14
ETFA_PSEAE	12 (11)	1050.27	Electron transfer flavoprotein subunit alpha	1.60E+05	1.59E+05	1.01	
BRAC_PSEAE	14 (7)	1029.12	Leucine-, isoleucine-, valine-, threonine-, and alanine-binding protein	1.36E+05	1.03E+05	1.32	
RS1_PSEAE	22 (20)	1017.46	30S ribosomal protein S1	5.66E+04	8.43E+04		1.49
DCTP_PSEAE	19 (15)	1016.64	C4-dicarboxylate-binding periplasmic protein DctP	8.38E+04	5.87E+04	1.43	
SYI_PSEAB	20 (2)	989.18	Isoleucine--tRNA ligase	2802	4436.1		1.58
ARCA_PSEAE	15 (8)	988	Arginine deiminase	2.44E+04	2.49E+04		1.02
ETFB_PSEAE	13 (8)	986.43	Electron transfer flavoprotein subunit beta	1.32E+05	1.80E+05		1.36
PPSA_PSEAB	19 (14)	969.97	Phosphoenolpyruvate synthase	3.27E+04	2.14E+04	1.53	
PHHC_PSEAE	17 (13)	921.36	Aromatic-amino-acid aminotransferase	2.83E+04	3.11E+04		1.10
SYP_PSEAE	15 (4)	857.67	Proline--tRNA ligase	1.38E+04	2.26E+04		1.64
RL1_PSEA7	14 (8)	849.1	50S ribosomal protein L1	1.02E+04	2.00E+04		1.96
GCSP1_PSEAE	17 (5)	845.26	Glycine dehydrogenase (decarboxylating) 1	2.27E+04	5827.6	3.90	

ACSA1_PSEAE	19 (10)	841.09	Acetyl-coenzyme A synthetase 1	1.16E+04	1.08E+04	1.07	
METK_PSEAB	15 (7)	840.53	S-adenosylmethionine synthase	2.48E+04	1.83E+04	1.36	
SAHH_PSEAB	17 (8)	836.44	Adenosylhomocysteinase	3.64E+04	4.30E+04		1.18
SPUD_PSEAB	16 (13)	834.85	Putrescine-binding periplasmic protein SpuD	7.03E+04	8.35E+04		1.19
METE_PSEAB	17 (9)	832.01	5-methyltetrahydropteroyltriglutamate--homocysteine methyltransferase	1.38E+04	2.87E+04		2.08
ATOB_PSEAE	18 (16)	824.87	Acetyl-CoA acetyltransferase	2.73E+04	6.03E+04		2.21
ASPQ_PSEAE	15 (6)	803.62	Glutaminase-asparaginase	2.94E+04	4.86E+04		1.65
SYV_PSEAE	18 (7)	797.74	Valine--tRNA ligase	2.57E+04	2.16E+04	1.19	
ODP1_PSEAE	18 (16)	791.89	Pyruvate dehydrogenase E1 component	1.95E+04	1.65E+04	1.18	
NUOG_PSEAE	18 (10)	766.43	NADH-quinone oxidoreductase subunit G	8138.43	8287.99		1.02
FLID1_PSEAI	15 (10)	762.33	A-type flagellar hook-associated protein 2	2.19E+04	2.94E+04		1.34
ODO2_PSEAE	12 (10)	757.77	Dihydrolipoyllysine-residue succinyltransferase component of 2-oxoglutarate dehydrogenase complex	1.74E+04	5837.01	2.98	
ISCS_PSEAB	12 (2)	753.02	Cysteine desulfurase IscS	6077.91	1.33E+04		2.19
TIG_PSEAB	16 (2)	750.85	Trigger factor	2855.81	8453.05		2.96
GYRA_PSEAE	14 (7)	736.16	DNA gyrase subunit A	8775.53	8120.61	1.08	
SYL_PSEAE	13 (1)	734.3	Leucine--tRNA ligase	5234.86	5110.83	1.02	
LOLA_PSEAB	13 (11)	731.34	Outer-membrane lipoprotein carrier protein	2.79E+04	1.99E+04	1.40	
TAL_PSEA8	13 (2)	720.4	Transaldolase	1.40E+04	1.54E+04		1.10
ACCA_PSEAB	12 (11)	717.91	Acetyl-coenzyme A carboxylase carboxyl transferase subunit alpha	1.49E+04	1.27E+04	1.17	
PORF_PSEAE	11 (11)	712.74	Outer membrane porin F	4.26E+04	5.25E+04		1.23
RPOA_PSEAB	14 (8)	712.69	DNA-directed RNA polymerase subunit alpha	1.07E+04	2.02E+04		1.89
SUCC_PSEAB	16 (2)	699.96	Succinate--CoA ligase [ADP-forming] subunit beta	3453.08	2460.04	1.40	
FABG_PSEAE	13 (11)	699.68	3-oxoacyl-[acyl-carrier-protein] reductase FabG	2.55E+04	6.41E+04		2.51
ARNB_PSEAB	10 (8)	688.59	UDP-4-amino-4-deoxy-L-arabinose--oxoglutarate aminotransferase	2.46E+04	2.48E+04		1.01
PCKA_PSEAE	14 (11)	685.84	Phosphoenolpyruvate carboxykinase (ATP)	1.80E+04	2.79E+04		1.55
P5217_PSEAE	11 (9)	676.19	Probable binding protein component of ABC iron transporter PA5217	1.57E+04	1.80E+04		1.15
CISY_PSEAE	16 (9)	666	Citrate synthase	1.48E+04	1.76E+04		1.19
SUCD_PSEAE	12 (9)	614.81	Succinate--CoA ligase [ADP-forming] subunit alpha	1.17E+05	1.19E+05		1.02
DPO3B_PSEAE	7 (6)	614.31	Beta sliding clamp	1.36E+04	2.48E+04		1.82

RL6_PSEA7	10 (8)	609.49	50S ribosomal protein L6	1.60E+04	5.88E+04		3.68
GREA_PSEAE	7 (5)	594.26	Transcription elongation factor GreA	2.44E+04	2.39E+04	1.02	
ODB2_PSEAE	10 (7)	591.02	Lipoamide acyltransferase component of branched-chain alpha-keto acid dehydrogenase complex	1.24E+04	2953.23	4.20	
THIO_PSEAE	12 (7)	580.45	Thioredoxin	6.02E+04	4.65E+04	1.29	
IF2_PSEA7	13 (2)	573.08	Translation initiation factor IF-2	4970.51	4140.37	1.20	
SPEE2_PSEAE	7 (3)	563.64	Polyamine aminopropyltransferase 2	3.71E+04	1.77E+04	2.10	
GLN1B_PSEAE	10 (6)	561.1	Glutamine synthetase	2.40E+04	3.17E+04		1.32
DAPA_PSEAE	8 (3)	557.93	4-hydroxy-tetrahydrodipicolinate synthase	1.07E+04	8716.06	1.23	
FUR_PSEAE	6 (6)	553.28	Ferric uptake regulation protein	3.32E+04	5.76E+04		1.73
PYRG_PSEAB	9 (4)	552.34	CTP synthase	6358.18	3235.86	1.96	
IF3_PSEAE	8 (4)	550.92	Translation initiation factor IF-3	1.84E+04	2.73E+04		1.48
AHPC_PSEAB	12 (10)	548.27	Alkyl hydroperoxide reductase C	1.28E+05	2.97E+05		2.32
TAL_PSEAB	11 (1)	541.47	Transaldolase	4563.31	5645.1		1.24
SYR_PSEA7	11 (4)	528.15	Arginine--tRNA ligase	3778.01	2597.07	1.45	
ETFD_PSEAE	11 (10)	524.29	Electron transfer flavoprotein-ubiquinone oxidoreductase	4939.44	1.19E+04		2.41
GRPE_PSEAB	7 (7)	523.42	Protein GrpE	1.48E+04	1.56E+04		1.05
PYS2_PSEAE	13 (9)	522.34	Pyocin-S2	1.55E+04	5645.46	2.75	
ILVD_PSEAB	9 (1)	512.24	Dihydroxy-acid dehydratase	3514.03	5302.22		1.51
KPRS_PSEAE	7 (7)	507.9	Ribose-phosphate pyrophosphokinase	8631.07	1.01E+04		1.17
ATPB_ALCBS	8 (1)	500.39	ATP synthase subunit beta	0.79	2219.1		2808.99
OTCC_PSEAE	11 (4)	498.67	Ornithine carbamoyltransferase, catabolic	3.80E+04	1.10E+04	3.45	
MMSA_PSEAE	7 (6)	493.68	Methylmalonate-semialdehyde dehydrogenase [acylating]	1.89E+04	1.68E+04	1.13	
ODP2_PSEAE	10 (6)	492.21	Dihydrolipoyllysine-residue acetyltransferase component of pyruvate dehydrogenase complex	1.45E+04	4023.28	3.60	
DAPB_PSEAB	10 (6)	491.77	4-hydroxy-tetrahydrodipicolinate reductase	1.26E+04	1.30E+04		1.03
NADE_PSEAB	9 (7)	491.04	NH(3)-dependent NAD(+) synthetase	1.68E+04	1.84E+04		1.10
OPGG_PSEAB	7 (5)	484.91	Glucans biosynthesis protein G	1.26E+04	1.40E+04		1.11
SECB_PSEAB	7 (7)	472.61	Protein-export protein SecB	2.00E+04	2.46E+04		1.23
HIS1_PSEAB	9 (2)	467.42	ATP phosphoribosyltransferase	2745.18	4464.05		1.63
AMPA_PSEAB	8 (2)	466.2	Probable cytosol aminopeptidase	5179.35	5350.63		1.03
RECA_PSEAB	9 (3)	462.09	Protein RecA	1.72E+04	7089.29	2.43	

DPO1_PSEAE	11 (7)	452.27	DNA polymerase I	5463.46	5618.92		1.03
ACCC_PSEAE	11 (10)	450.88	Biotin carboxylase	2.15E+04	1.75E+04	1.23	
ECOT_PSEAB	8 (4)	449.08	Ecotin	4.46E+04	2.03E+04	2.20	
GGT_PSEAE	8 (5)	435.04	Gamma-glutamyltranspeptidase	1.13E+04	1.17E+04		1.04
HUTU_PSEAB	10 (1)	434.15	Urocanate hydratase	2071.58	1383.37	1.50	
AZUR_PSEAE	5 (3)	434.1	Azurin	3.99E+05	2.97E+05	1.34	
CH10_PSEA7	8 (5)	431.92	10 kDa chaperonin	9.20E+04	8.63E+04	1.07	
KAD_PSEAB	8 (1)	431.41	Adenylate kinase	2863.62	1876.68	1.53	
GSHB_PSEAE	11 (8)	428.33	Glutathione synthetase	7320.31	6010.22	1.22	
TPX_PSEAE	8 (6)	424.99	Thiol peroxidase	5.82E+04	8.25E+04		1.42
ARUC_PSEAE	12 (11)	421.57	Succinylornithine transaminase/acetylornithine aminotransferase	1.23E+04	1.86E+04		1.51
Y1579_PSEAE	9 (8)	417.56	Uncharacterized protein PA1579	4.41E+04	2.16E+04	2.04	
MMSB_PSEAE	10 (8)	417.47	3-hydroxyisobutyrate dehydrogenase	5.47E+04	3.66E+04	1.49	
DBHB_PSEAE	6 (3)	413.62	DNA-binding protein HU-beta	9.10E+04	4.70E+04	1.94	
RL9_PSEA7	7 (5)	413.31	50S ribosomal protein L9	2.54E+04	4.99E+04		1.96
NUSG_PSEAE	8 (5)	413.07	Transcription termination/antitermination protein NusG	4583	1.01E+04		2.20
RL5_PSEA8	7 (1)	411.52	50S ribosomal protein L5	1.33E+04	2.31E+04		1.74
RL25_PSEA8	8 (2)	406.94	50S ribosomal protein L25	1.23E+04	6979.58	1.76	
PGK_PSEAB	7 (3)	400.45	Phosphoglycerate kinase	4847.58	6179.27		1.27
TAL_PSEA7	9 (1)	399.28	Transaldolase	1797.22	2130.44		1.19
RS8_PSEA7	5 (5)	398.36	30S ribosomal protein S8	2.10E+04	2.06E+04	1.02	
AAT_PSEAE	7 (5)	393.37	Aspartate aminotransferase	5623.48	5546.68	1.01	
SURA_PSEAE	8 (6)	388.75	Chaperone SurA	8547.91	1.34E+04		1.57
RL7_PSEA7	9 (9)	388.04	50S ribosomal protein L7/L12	1.97E+04	2069.11	9.52	
HEM6_PSEA8	9 (7)	387.92	Oxygen-dependent coproporphyrinogen-III oxidase	5.49E+04	2.94E+04	1.87	
PH4H_PSEAE	8 (7)	383.57	Phenylalanine-4-hydroxylase	1.62E+04	2.89E+04		1.78
NDK_PSEAB	7 (6)	377.96	Nucleoside diphosphate kinase	8.48E+04	1.51E+05		1.78
IPYR_PSEAE	6 (5)	372.96	Inorganic pyrophosphatase	1.98E+04	3.63E+04		1.83
ACCD_PSEAB	6 (2)	372.67	Acetyl-coenzyme A carboxylase carboxyl transferase subunit beta	9875.83	9872.35	1.00	
RPOC_WIGBR	11 (1)	371.29	DNA-directed RNA polymerase subunit beta'	0	451.79		∞

SYK_PSEAE	7 (2)	369.73	Lysine--tRNA ligase	2952.4	4870.33		1.65
SKPL_PSEAE	4 (3)	362.98	Skp-like protein	1.94E+04	1.72E+04	1.13	
RS6_PSEA7	5 (4)	361.9	30S ribosomal protein S6	1.01E+04	1.41E+04		1.40
PURA_PSEAB	5 (2)	359.93	Adenylosuccinate synthetase	8507.77	1.36E+04		1.60
SODF_PSEAE	7 (6)	359.09	Superoxide dismutase [Fe]	8.44E+04	1.32E+05		1.56
SUCC_PSEAE	10 (2)	351.04	Succinate--CoA ligase [ADP-forming] subunit beta	5882.18	2523.52	2.33	
PHS_PSEAB	6 (6)	349.57	Putative pterin-4-alpha-carbinolamine dehydratase	9.66E+04	9.33E+04	1.04	
ARCC_PSEAE	5 (3)	349.52	Carbamate kinase	1.22E+04	6624.67	1.84	
SSB_PSEAE	7 (7)	346.74	Single-stranded DNA-binding protein	3.43E+04	5.30E+04		1.55
HEM2_PSEAE	6 (4)	343.44	Delta-aminolevulinic acid dehydratase	5092.04	6972.84		1.37
NIRQ_PSEAE	7 (7)	340.65	Denitrification regulatory protein NirQ	1.53E+04	3616.07	4.23	
FADA_PSEAB	5 (4)	339.96	3-ketoacyl-CoA thiolase	6239.5	5428.93	1.15	
STHA_PSEAB	7 (6)	335.15	Soluble pyridine nucleotide transhydrogenase	4272.6	6423.06		1.50
CLPP1_PSEAE	6 (5)	334.95	ATP-dependent Clp protease proteolytic subunit 1	1.83E+04	1.13E+04	1.62	
SYGA_PSEAB	5 (5)	333.22	Glycine--tRNA ligase alpha subunit	4696.47	5627.9	0.83	1.20
PPIA_PSEAE	6 (5)	331.2	Peptidyl-prolyl cis-trans isomerase A	2.19E+04	1.35E+04	1.62	
CYNS_PSEAE	7 (2)	330.23	Cyanate hydratase	7693.32	1.60E+04		2.08
RL25_PSEA7	7 (2)	329.59	50S ribosomal protein L25	5.74E+04	8080.55	7.10	
NFUA_PSEAB	6 (5)	328.6	Fe/S biogenesis protein NfuA	1.12E+04	1.04E+04	1.08	
SYW_PSEAE	5 (4)	326.71	Tryptophan--tRNA ligase	4303.23	7981.1		1.85
EDD_PSEAE	7 (5)	325.84	Phosphogluconate dehydratas	1.03E+04	1.42E+04		1.38
DHOM_PSEAE	5 (4)	320.51	Homoserine dehydrogenase	3282.37	5097.24		1.55
AK_PSEAE	10 (4)	319.6	Aspartokinase	1.77E+04	1.92E+04		1.08
PYRE_PSEAB	5 (1)	313.66	Orotate phosphoribosyltransferase	1.68E+04	2.49E+04		1.48
CARB_PSEAE	7 (3)	310.67	Carbamoyl-phosphate synthase large chain	4451.2	1415.82	3.14	
GPMI_PSEAE	5 (1)	309.41	2,3-bisphosphoglycerate-independent phosphoglycerate mutase	3846.13	5155.66		1.34
HSCA_PSEA8	8 (5)	309.34	Chaperone protein HscA homolog	3357.62	6098.37		1.82
SYT_PSEAB	8 (2)	301.28	Threonine--tRNA ligase	1986.78	2137.08		1.08

LIUE_PSEAE	6 (4)	298.31	3-hydroxy-3-isohexenylglutaryl-CoA/hydroxy-methylglutaryl-CoA lyase	5797.97	5138.89	1.13	
GLMS_PSEAE	6 (5)	295.78	Glutamine--fructose-6-phosphate aminotransferase [isomerizing]	2732.86	3696.9		1.35
GAP2_PSEAB	12 (7)	295.31	Glyceraldehyde-3-phosphate dehydrogenase-like protein	3.17E+04	3.70E+04		1.17
ARGJ_PSEAE	5 (5)	294.91	Arginine biosynthesis bifunctional protein ArgJ	8066.95	7029.44	1.15	
AMPC_PSEAE	6 (5)	290.82	Beta-lactamase	1.07E+04	3359.88	3.18	
GPMI_PSEAB	5 (1)	288.27	2,3-bisphosphoglycerate-independent phosphoglycerate mutase	2096.44	3298.42		1.57
PAL_PSEAE	6 (5)	288.11	Peptidoglycan-associated lipoprotein	1.49E+04	2007.79	7.42	
RL3_PSEA7	7 (4)	287.29	50S ribosomal protein L3	1.19E+04	1.54E+04		1.29
PDXH_PSEA8	9 (7)	282.32	Pyridoxine/pyridoxamine 5'-phosphate oxidase	1.03E+04	9836.74	1.05	
ILVC_PSEAB	7 (5)	281.28	Ketol-acid reductoisomerase (NADP(+))	6495.11	2831.48	2.29	
EFP_PSEAB	5 (4)	278.31	Elongation factor P	2.09E+04	1.52E+04	1.38	
GSHR_PSEAE	5 (4)	277.37	Glutathione reductase	7148.56	5108.21	1.40	
SPUE_PSEAE	8 (5)	271.33	Spermidine-binding periplasmic protein SpuE	1.78E+04	1.70E+04	1.05	
SYY2_PSEAE	6 (1)	271.25	Tyrosine--tRNA ligase 2	1467.32	3952.98		2.69
ARNA_PSEA7	6 (1)	269.02	Bifunctional polymyxin resistance protein ArnA	2659.5	374.08	7.11	
THIG_PSEAB	6 (6)	267.8	Thiazole synthase	9070.97	8488.37	1.07	
HEMTB_PSEAB	5 (3)	266.79	Bacteriohemerythrin	5734.71	2.11E+04		3.68
EST_PSEAE	4 (4)	266.04	Esterase TesA	6449.51	3168.14	2.04	
ACKA_PSEA7	6 (5)	263.69	Acetate kinase	4934.95	7203.99		1.46
ENO_PSEAB	5 (2)	259.9	Enolase	3856.19	3095.77	1.25	
PANB2_PSEAB	6 (6)	259.18	3-methyl-2-oxobutanoate hydroxymethyltransferase 2	1.26E+04	8243.72	1.53	
Y4395_PSEAE	6 (3)	254.32	UPF0234 protein PA4395	9633.76	6039.82	1.60	
FABA_PSEAB	5 (4)	251.54	3-hydroxydecanoyl-[acyl-carrier-protein] dehydratase	1.41E+04	3.07E+04		2.18
FABB_PSEAB	4 (4)	251.23	3-oxoacyl-[acyl-carrier-protein] synthase 1	1.40E+04	3.00E+04		2.14
RS7_PSEA7	6 (1)	246.72	30S ribosomal protein S7	7041.66	4160.74	1.69	
ISPF_PSEAE	3 (1)	245.98	2-C-methyl-D-erythritol 2,4-cyclodiphosphate synthase	8084.96	9846.36		1.22
DAPD_PSEAE	6 (2)	245.64	2,3,4,5-tetrahydropyridine-2,6-dicarboxylate N-succinyltransferase	4575.19	3907.22	1.17	
RL11_PSEA7	3 (1)	244.82	50S ribosomal protein L11	1.43E+04	2.16E+04		1.51
P5CR_PSEAE	5 (5)	241.96	Pyrroline-5-carboxylate reductase	6272.59	7823.96		1.25
Y329_PSEAE	4 (4)	237.64	UPF0339 protein PA0329	1.39E+04	1.14E+04	1.22	

PURE_PSEAE	4 (3)	237.38	N5-carboxyaminoimidazole ribonucleotide mutase	4163.8	4709.89		1.13
RL18_PSEA7	3 (1)	237.2	50S ribosomal protein L18	7362.17	4736.09	1.55	
TRPC_PSEAE	4 (2)	237.15	Indole-3-glycerol phosphate synthase	2528.71	5527.89		2.19
GUAA_PSEAB	4 (3)	237.07	GMP synthase [glutamine-hydrolyzing]	1.69E+04	1.90E+04		1.12
PUR7_PSEA8	5 (3)	236.9	Phosphoribosylaminoimidazole-succinocarboxamide synthase	7905.56	5686.18	1.39	
RRAAH_PSEAB	5 (3)	236.5	Putative 4-hydroxy-4-methyl-2-oxoglutarate aldolase	1.82E+04	3.17E+04		1.74
ACEA_PSEAE	6 (5)	236.48	Isocitrate lyase	6225.4	2142.21	2.91	
BAUC_PSEAE	6 (5)	236.29	Putative 3-oxopropanoate dehydrogenase	1.20E+04	4565.74	2.63	
PYRE_PSEA7	5 (1)	235.39	Orotate phosphoribosyltransferase	1489.23	1342.12	1.11	
G3P_PSEAE	4 (3)	233.25	Glyceraldehyde-3-phosphate dehydrogenase	9565.61	4021.94	2.38	
ASPA_PSEAE	9 (6)	229.08	Aspartate ammonia-lyase	6780.96	1.01E+04		1.49
DEF_PSEAE	4 (4)	229.04	Peptide deformylase	4196.66	5942.49		1.42
FMNRE_PSEAE	5 (5)	227.89	NAD(P)H-dependent FMN reductase PA1204	2.54E+05	1.19E+04	21.34	
QOR_PSEAE	4 (3)	227.66	Quinone oxidoreductase	2502.61	761.04	3.29	
LEU1_PSEAE	6 (5)	225.99	2-isopropylmalate synthase	4360.91	1613.55	2.70	
CYC4_PSEAE	5 (2)	225.63	Cytochrome c4	5180.75	2444.33	2.12	
FADB_PSESM	6 (1)	224.77	Fatty acid oxidation complex subunit alpha	2.11E+04	1.50E+04	1.41	
GATB_PSEA7	4 (3)	222.43	Aspartyl/glutamyl-tRNA(Asn/Gln) amidotransferase subunit B	3395.07	5646.2		1.66
RS7_PSE14	6 (1)	221.91	30S ribosomal protein S7	1.48E+04	1.20E+04	1.23	
MUCB_PSEAE	4 (4)	220.75	Sigma factor AlgU regulatory protein MucB	7846.6	3739.36	2.10	
PSTS_PSEAB	4 (4)	220.27	Phosphate-binding protein PstS	1.27E+04	9833.48	1.29	
UPP_PSEAB	6 (1)	218.9	Uracil phosphoribosyltransferase	3.28E+04	5.93E+04		1.81
ELAS_PSEAB	4 (3)	217.95	Elastase	3.67E+04	2.04E+04	1.80	
NUOB_PSEAB	5 (3)	216.74	NADH-quinone oxidoreductase subunit B	1849.28	2993.59		1.62
ODBB_PSEAE	6 (4)	216.63	2-oxoisovalerate dehydrogenase subunit beta	7444.49	5097.76	1.46	
HCHA_PSEAB	5 (4)	213.8	Protein deglycase HchA	6000.01	1894.32	3.17	
FABZ_PSEAB	5 (4)	213.8	3-hydroxyacyl-[acyl-carrier-protein] dehydratase FabZ	5298.18	7111.24		1.34
RISB_PSEAB	5 (5)	212.32	6,7-dimethyl-8-ribityllumazine synthase	1.14E+04	6465.79	1.76	
SYFA_PSEA8	6 (2)	209.71	Phenylalanine--tRNA ligase alpha subunit	741.59	2288.72		3.09
HCP1_PSEAE	6 (3)	209.57	Protein hcp1	4483.28	1.80E+04		4.01

UBIE_PSEAB	3 (3)	208.05	Ubiquinone/menaquinone biosynthesis C-methyltransferase UbiE	4990.16	6069.48		1.22
AMIE_PSEAE	4 (3)	204.49	Aliphatic amidase	3276.9	1.10E+04		3.36
MTIP_PSEAE	4 (4)	202.16	S-methyl-5'-thioinosine phosphorylase	2934.62	2914.36	1.01	
GMHA_PSEAB	3 (3)	198.53	Phosphoheptose isomerase	9468.14	1.34E+04		1.42
PYRR_PSEAB	5 (5)	198.07	Bifunctional protein PyrR	1.23E+04	1.16E+04	1.06	
CYSM_PSEAE	6 (5)	196.84	Cysteine synthase B	1.58E+04	9042.08	1.75	
GATA_PSEA7	4 (2)	195.39	Glutamyl-tRNA(Gln) amidotransferase subunit A	668.87	1368.24		2.05
ACP1_PSEAE	2 (1)	195.3	Acyl carrier protein 1	1.20E+05	1.57E+05		1.31
SYQ_PSEAB	5 (2)	195.27	Glutamine--tRNA ligase	2.20E+04	1.86E+04	1.18	
SYFB_PSEAE	7 (4)	195.22	Phenylalanine--tRNA ligase beta subunit	2682.02	2891.91		1.08
PROA_PSEAB	4 (3)	194.45	Gamma-glutamyl phosphate reductase	2142.72	3763.24		1.76
DHAS_PSEAE	4 (2)	193.89	Aspartate-semialdehyde dehydrogenase	3.29E+04	3.23E+04	1.02	
PILH_PSEAE	3 (2)	191.49	Protein PilH	5006.9	1495.94	3.35	
RL24_PSEA7	6 (6)	190.81	50S ribosomal protein L24	9267.1	1.07E+04		1.15
LAP_PSEAB	4 (2)	190.72	Aminopeptidase	5339.96	2941.41	1.82	
MSRA_PSEAE	4 (2)	187.52	Peptide methionine sulfoxide reductase MsrA	8414.31	9049.94		1.08
ILVE_PSEAE	4 (4)	184.41	Branched-chain-amino-acid aminotransferase	2.31E+04	2.41E+04		1.04
METZ_PSEAE	5 (3)	184.21	O-succinylhomoserine sulfhydrylase	1785.12	1633.36	1.09	
CYSNC_PSEAE	4 (2)	183.61	Bifunctional enzyme CysN/CysC	2004.09	2296.45		1.15
NUOCD_PSEAB	4 (4)	180.99	NADH-quinone oxidoreductase subunit C/D	1672.19	2335.19		1.40
RL19_PSEA7	4 (2)	179.15	50S ribosomal protein L19	6710.12	5009.57	1.34	
PURT_PSEAB	3 (1)	177.56	Formate-dependent phosphoribosylglycinamide formyltransferase	2420.16	4065.35		1.68
ALGP_PSEAE	3 (3)	176.77	Transcriptional regulatory protein AlgP	2561.65	3771.97		1.47
RPOB_ACIF2	14 (3)	175.57	DNA-directed RNA polymerase subunit beta	336.96	465.49		1.38
LPXC_PSEAB	4 (3)	171.36	UDP-3-O-acyl-N-acetylglucosamine deacetylase	2956.82	3405.28		1.15
DBHA_PSEAE	2 (1)	170.93	DNA-binding protein HU-alpha	2163.61	21.12	102.44	
GCSH1_PSEAE	3 (3)	170.92	Glycine cleavage system H protein 1	1.56E+04	4848.33	3.22	
Y3435_PSEAE	3 (3)	170.67	Uncharacterized protein PA3435	5499.06	3277.42	1.68	
COAD_PSEAB	3 (3)	169.13	Phosphopantetheine adenylyltransferase	3910.38	6638.45		1.70
Y4465_PSEAE	3 (2)	169.09	Nucleotide-binding protein PA4465	4179.44	1581.58	2.64	

ARGC_PSEA7	4 (1)	164.45	N-acetyl-gamma-glutamyl-phosphate reductase	1617.33	2608.91		1.61
THRC_PSEAE	4 (2)	162.56	Threonine synthase	1060.54	1815.48		1.71
PHEA_PSEAE	3 (2)	161.97	P-protein	1290.74	1856.03		1.44
CCPR_PSEAE	3 (3)	160.03	Cytochrome c551 peroxidase	9513.92	119.03	79.93	
MOAC_PSEAB	3 (2)	158.94	Cyclic pyranopterin monophosphate synthase	4504.06	9865.4		2.19
ATPG_PSEAB	6 (3)	158.77	ATP synthase gamma chain	843.34	874.22		1.04
HFQ_PSEAB	4 (4)	158.44	RNA-binding protein Hfq	7851.92	2684.42	2.92	
OTCA_PSEAE	4 (3)	156.56	Ornithine carbamoyltransferase, anabolic	1982.71	1820.23	1.09	
CHMU_PSEAE	3 (2)	155.43	Monofunctional chorismate mutase	3840.37	3053.91	1.26	
LEU3_PSEAE	4 (3)	154.78	3-isopropylmalate dehydrogenase	2541.32	3327.12		1.31
THTR_PSEAE	2 (2)	154.59	Thiosulfate sulfurtransferase	2669.62	991.96	2.69	
GLPK2_PSEAE	4 (4)	153.63	Glycerol kinase 2	4077	7616.46		1.87
FUMC1_PSEAE	6 (3)	153.23	Fumarate hydratase class II 1	4550.69	4839.75		1.06
KDSB_PSEAB	2 (2)	150.73	3-deoxy-manno-octulosonate cytidylyltransferase	1601.76	2047.4		1.28
ATPE_PSEAB	3 (3)	149.51	ATP synthase epsilon chain	9457.96	4492.74	2.11	
GSA_PSEAB	5 (2)	146.66	Glutamate-1-semialdehyde 2,1-aminomutase	927.94	3276.91		3.53
PORD_PSEAE	4 (4)	145.95	Porin D	2415.09	543.61	4.44	
ATPD_PSEAB	3 (2)	145.65	ATP synthase subunit delta	4974.3	1.35E+04		2.71
ODBA_PSEAE	4 (3)	144.44	2-oxoisovalerate dehydrogenase subunit alpha	3143.38	3458.41		1.10
RHO_PSEAE	6 (3)	141.08	Transcription termination factor Rho	1617.37	4537.28		2.81
PYRC_PSEAB	2 (2)	139.87	Dihydroorotase	2996.93	7169.31		2.39
Y2116_PSEAE	3 (2)	139.34	Putative hydro-lyase PA2116	7664.13	1.23E+04		1.60
PDXY_PSEAB	3 (2)	138.19	Pyridoxal kinase PdxY	3146.08	1591.48	1.98	
MAO1_PSEA7	4 (1)	137.79	NAD-dependent malic enzyme	3824.81	4443.71		1.16
SYA_PSEAB	6 (5)	136.16	Alanine--tRNA ligase	2.66E+04	1.59E+04	1.67	
BETB_PSEAB	2 (1)	133.52	NAD/NADP-dependent betaine aldehyde dehydrogenase	4023.32	3156.7	1.27	
Y1607_PSEA8	3 (3)	132.03	UPF0502 protein PLES_16071	1366.85	4447.1		3.25
SECA_PSEAB	6 (2)	131.11	Protein translocase subunit SecA	1733.4	80.47	21.54	
CY551_PSEAE	2 (2)	130.87	Cytochrome c-551	1.23E+04	9199.89	1.34	
GLO2_PSEA8	2 (2)	130.16	Hydroxyacylglutathione hydrolase	8582.73	6250	1.37	

SYM_PSEAB	4 (3)	129.46	Methionine--tRNA ligase	1522.1	445.05	3.42	
BFR_PSEAE	4 (4)	126.55	Bacterioferritin	2184.01	5793.98		2.65
KDSA_PSEAB	3 (1)	126.17	2-dehydro-3-deoxyphosphooctonate aldolase	1089.39	1241.66		1.14
HISX_PSEAE	3 (2)	124.92	Histidinol dehydrogenase	2.33E+04	1.50E+04	1.55	
FLIA_PSEAE	3 (2)	124.8	RNA polymerase sigma factor FliA	4558.44	3870.55	1.18	
PLPHP_PSEAE	2 (2)	123.99	Pyridoxal phosphate homeostasis protein	1939.13	3242.86		1.67
TOLB_PSEAE	3 (2)	122.19	Protein TolB	2172.9	6111.5		2.81
BCCP_PSEAE	2 (2)	121.86	Biotin carboxyl carrier protein of acetyl-CoA carboxylase	2.11E+04	4.52E+04		2.14
Y4473_PSEAE	2 (1)	121.22	UPF0307 protein PA4473	856.95	1330.09		1.55
Y3753_PSEAE	2 (2)	120	Uncharacterized protein PA3753	1.37E+04	1.34E+04	1.02	
MINE_PSEAB	3 (2)	118.49	Cell division topological specificity factor	2279.78	356.39	6.40	
RS2_PSEA7	2 (2)	116.29	30S ribosomal protein S2	5951.92	1241.66	4.79	
MURA_PSEAB	4 (1)	114.72	UDP-N-acetylglucosamine 1-carboxyvinyltransferase	292.76	820.74		2.80
SUHB_PSEAE	3 (1)	114.24	Inositol-1-monophosphatase	7154.75	1.08E+04		1.51
GCH12_PSEAE	3 (3)	113.28	GTP cyclohydrolase 1 2	2126.22	3912.12		1.84
RPIA_PSEAB	3 (2)	113.17	Ribose-5-phosphate isomerase A	2252.37	1008.16	2.23	
PUR8_PSEAE	4 (2)	113.15	Adenylosuccinate lyase	2760.35	1949.21	1.42	
ACSA2_PSEAE	3 (2)	112.99	Acetyl-coenzyme A synthetase 2	3769	3483.85	1.08	
PTSN_PSEAE	4 (3)	112.24	Nitrogen regulatory protein	1.07E+04	1.61E+04		1.50
SYA_PSEA7	5 (3)	108.06	Alanine--tRNA ligase	1.01E+04	4.34E+04		4.30
PUR4_PSEAE	7 (3)	107.81	Phosphoribosylformylglycinamidine synthase	5129.72	628.29	8.16	
SYE_PSEAB	3 (2)	106.79	Glutamate--tRNA ligase	2238.59	2441.28		1.09
RL16_PSEA7	3 (3)	104.57	50S ribosomal protein L16	1141.09	316.73	3.60	
TPIS_PSEAB	2 (1)	104.52	Triosephosphate isomerase	2177.28	2146.85	1.01	
PYRB_PSEAB	3 (1)	104.49	Aspartate carbamoyltransferase	1983.82	598	3.32	
RL22_PSEA7	3 (3)	104.02	50S ribosomal protein L22	5443.61	7067.85		1.30
PUR2_PSEAE	2 (2)	100.91	Phosphoribosylamine--glycine ligase	445.13	1491.72		3.35
RL21_PSEA7	3 (3)	100.06	50S ribosomal protein L21	3922.25	4801.24		1.22
RL20_PSEA7	2 (1)	99.59	50S ribosomal protein L20	6143.02	1631.31	3.77	
LIP_PSEAE	1 (1)	98.65	Triacylglycerol lipase	4142.15	61.6	67.24	

DDLB_PSEAE	1 (1)	97.69	D-alanine--D-alanine ligase B	799.87	152.98	5.23	
ANR_PSEAE	2 (2)	96.85	Transcriptional activator protein anr	1268.28	2774.62		2.19
PQSD_PSEAE	2 (2)	96.67	Anthraniloyl-CoA anthraniloyltransferase	1695.56	3175.86		1.87
SODM_PSEAE	2 (2)	95.28	Superoxide dismutase [Mn]	951.74	3602.31		3.78
6PGL_PSEPU	3 (1)	94.83	6-phosphogluconolactonase	2688.29	4048.74		1.51
SYGB_PSEAB	2 (2)	94.59	Glycine--tRNA ligase beta subunit	3692.65	3246.07	1.14	
NUOE_PSEAE	1 (1)	94.25	NADH-quinone oxidoreductase subunit E	3057.7	6175.55		2.02
MURD_PSEAE	4 (4)	94.07	UDP-N-acetylmuramoylalanine--D-glutamate ligase	1376.49	1368.5	1.01	
HIS4_PSEA7	2 (2)	92.91	1-(5-phosphoribosyl)-5-[(5-phosphoribosylamino)methylideneamino] imidazole-4-carboxamide isomerase	4454.88	3962.15	1.12	
Y1618_PSEAE	2 (2)	92.64	Putative esterase PA1618	706.97	1600.3		2.26
HGD_PSEAE	2 (1)	91.6	Homogentisate 1,2-dioxygenase	14.89	1750.94		117.59
CLPP2_PSEAE	1 (1)	91.09	ATP-dependent Clp protease proteolytic subunit 2	1288	461.16	2.79	
HISZ_PSEAB	2 (2)	90.98	ATP phosphoribosyltransferase regulatory subunit	6461.65	8702.23		1.35
TRPG_PSEAE	1 (1)	90.81	Anthranilate synthase component 2	5036.24	4987.34	1.01	
COAE_PSEAE	2 (2)	90.69	Dephospho-CoA kinase	932.39	841.31	1.11	
RSMH_PSEA8	3 (2)	90.06	Ribosomal RNA small subunit methyltransferase H	4412.18	5657.03		1.28
DCDA_PSEAE	1 (1)	89.02	Diaminopimelate decarboxylase	872.86	1047		1.20
FMCD_PSEAI	1 (1)	87.87	Fimbrial protein	1.12E+04	6779.89	1.65	
AROB_PSEAB	3 (1)	87.54	3-dehydroquinate synthase	2764.32	3210.57		1.16
PANC_PSEAB	2 (2)	87.32	Pantothenate synthetase	5200.57	6638.23		1.28
MURE_PSEAE	2 (2)	87.18	UDP-N-acetylmuramoyl-L-alanyl-D-glutamate--2,6-diaminopimelate ligase	217.63	342.22		1.57
HGD_PSEAB	3 (2)	85.02	Homogentisate 1,2-dioxygenase	451.95	7365.51		16.30
PYRX_PSEAE	4 (2)	83.95	Dihydroorotase-like protein	4099.27	958.31	4.28	
ASTB_PSEAB	2 (2)	83.54	N-succinylarginine dihydrolase	3250.66	3438.95		1.06
ACND_SHEON	1 (1)	82.65	2-methylcitrate dehydratase (2-methyl-trans-aconitate forming)	3432.91	1313.62	2.61	
APT_PSEAB	3 (2)	81.68	Adenine phosphoribosyltransferase	1798.76	2291.71		1.27
HEMN_PSEAE	2 (1)	81.66	Oxygen-independent coproporphyrinogen III oxidase	1003.36	149.27	6.72	
LDC_PSEAE	1 (1)	81.62	Murein tetrapeptide carboxypeptidase	1529.81	739.13	2.07	
RS9_PSEA7	2 (2)	81.53	30S ribosomal protein S9	2186.93	855.72	2.56	

EUTC_PSEAB	1 (1)	80.25	Ethanolamine ammonia-lyase light chain	88.47	572.84		6.47
DADA1_PSEAE	3 (2)	79.06	D-amino acid dehydrogenase 1	7919.32	2585.74	3.06	
PPK_PSEAE	3 (3)	78.82	Polyphosphate kinase	556.29	501.25	1.11	
CAPP_PSEAE	2 (1)	78.55	Phosphoenolpyruvate carboxylase	1.23E+04	1.44E+04		1.17
AROQ1_PSEAE	1 (1)	78.33	3-dehydroquinate dehydratase 1	1.71E+04	1.10E+04	1.55	
RS14_PSEA7	1 (1)	78.3	30S ribosomal protein S14	548.56	0	∞	
SYS_PSEAB	2 (2)	77.57	Serine--tRNA ligase	2794.55	4354.81		1.56
DCD_PSEAB	2 (2)	77.37	dCTP deaminase	6970.86	1.13E+04		1.62
PUR1_PSEAE	1 (1)	76.93	Amidophosphoribosyltransferase	2573.08	3.05	843.63	
TRPA_PSEAB	1 (1)	76.56	Tryptophan synthase alpha chain	8095.02	1.21E+04		1.49
ESTA_PSEAE	1 (1)	74.32	Esterase EstA	881.8	304.85	2.89	
KDSA1_PSEPK	4 (2)	74.23	2-dehydro-3-deoxyphosphooctonate aldolase 1	1.77E+04	1.93E+04		1.09
HSLV_PSEAB	1 (1)	74.14	ATP-dependent protease subunit HslV	1.35E+04	1.19E+04	1.13	
LGUL_PSEAE	2 (2)	74.13	Lactoylglutathione lyase	1757.47	609.92	2.88	
GPDA_PSEAB	2 (2)	73.58	Glycerol-3-phosphate dehydrogenase [NAD(P)+]	731.81	1386.62		1.89
RS20_PSEA7	1 (1)	71.66	30S ribosomal protein S20	1.25E+04	9441.53	1.32	
FER_PSEAE	1 (1)	71.52	2Fe-2S ferredoxin	881.16	3066.23		3.48
TRPB_PSEAE	2 (2)	70.41	Tryptophan synthase beta chain	1.72E+04	5240.46	3.28	
OADC_PSEAB	3 (2)	70.24	Oxaloacetate decarboxylase	1032.02	972.05	1.06	
HLDD_PSEAB	1 (1)	70.08	ADP-L-glycero-D-manno-heptose-6-epimerase	1734.4	203.33	8.53	
ERPA_PSEAB	1 (1)	70.02	Iron-sulfur cluster insertion protein ErpA	9462.38	8165.97	1.16	
BEKAS_PSEAE	3 (1)	69.15	Beta-ketodecanoyl-[acyl-carrier-protein] synthase	2712.18	4620.2		1.70
OPRI_PSEAE	1 (1)	68.66	Major outer membrane lipoprotein	368.13	324.99	1.13	
UVRB_PSEAE	2 (1)	68.08	UvrABC system protein B	0	386.56		∞
FOLD_PSEAB	1 (1)	67.79	Bifunctional protein FolD	4932.86	4630.81	1.07	
DSBG_PSEAE	1 (1)	67.3	Thiol:disulfide interchange protein DsbG	2232	3266.29	0.68	1.46
SURE_PSEAB	1 (1)	66.3	5'-nucleotidase SurE	2824.26	2613.95	1.08	
NUOF_PSEAE	1 (1)	65.2	NADH-quinone oxidoreductase subunit F	2039.46	287.3	7.10	
NORC_PSEAE	2 (2)	65.14	Nitric oxide reductase subunit C	4825.12	133.58	36.12	
F16PA_PSEA7	1 (1)	64.82	Fructose-1,6-bisphosphatase class 1	2045.23	4179.58		2.04

DNAJ_PSEAB	2 (1)	63.85	Chaperone protein DnaJ	4216.5	3326.05	1.27	
HUTI_PSEAB	2 (2)	63.21	Imidazolonepropionase	1773.85	629.57	2.82	
T23O_PSEAB	1 (1)	62.33	Tryptophan 2,3-dioxygenase	1744.13	1319.65	1.32	
COBN_PSEAE	3 (2)	61.19	Aerobic cobaltochelatase subunit CobN	743.38	669.21	1.11	
AGUA_PSEAB	1 (1)	61.08	Agmatine deiminase	3462.83	3340.99	1.04	
PRTN_PSEAE	2 (2)	60.87	Transcription regulatory protein PrtN	4.04E+04	4.49E+04		1.11
BAUA_PSEAE	2 (1)	60.7	Beta-alanine--pyruvate aminotransferase	6036.88	4824.03	1.25	
HIS7_PSEA7	2 (1)	60.31	Imidazoleglycerol-phosphate dehydratase	1449.94	175.09	8.28	
MTND_PSEAB	1 (1)	59.33	Acireductone dioxygenase	4197.37	1304.43	3.22	
ASTD_PSEAE	1 (1)	58.06	N-succinylglutamate 5-semialdehyde dehydrogenase	2189.27	724.68	3.02	
RS17_PSEA7	1 (1)	56.64	30S ribosomal protein S17	2694.45	1313.2	2.05	
Y2077_PSEAB	1 (1)	56.51	STAS-domain containing protein PA14_20770	1481	303.15	4.89	
GLMU_PSEAB	2 (2)	55.44	Bifunctional protein GlmU	2778.68	1273.58	2.18	
GALU_PSEAE	2 (1)	54.72	UTP--glucose-1-phosphate uridylyltransferase	479.76	520.09		1.08
G6PI_PSEA7	3 (1)	54.61	Glucose-6-phosphate isomerase	1812.76	1469.3	1.23	
PHEC_PSEAE	1 (1)	53.92	Cyclohexadienyl dehydratase	2652.87	676.01	3.92	
YGFB_PSEAE	1 (1)	53.24	UPF0149 protein PA5225	2497.78	362.97	6.88	
HIS3_PSEA7	1 (1)	52.3	Phosphoribosyl-AMP cyclohydrolase	6338.31	785.18	8.07	
Y3332_PSEAE	1 (1)	50.99	Uncharacterized PhzA/B-like protein PA3332	1542.84	1574.66		1.02
CSY3_PSEAB	1 (1)	49.73	CRISPR-associated protein Csy3	2949.66	2751.96	1.07	
Y2972_PSEAE	1 (1)	48.35	Maf-like protein PA2972	377.27	303.83	1.24	
ARNA_PSEAB	2 (1)	47.18	Bifunctional polymyxin resistance protein ArnA	7389.39	5375.54	1.37	
Y978_PSEA8	1 (1)	46.79	UPF0250 protein PLES_09781	1799.46	541.38	3.32	
FLIN_PSEAE	1 (1)	46.57	Flagellar motor switch protein FliN	2785.12	1607.54	1.73	
RS3_PSEA7	2 (2)	44.87	30S ribosomal protein S3	2222.98	2327.11		1.05
ALKD_PSEAE	1 (1)	44.72	2-dehydro-3-deoxy-phosphogluconate aldolase	619.82	518.61	1.20	
GCH11_PSEAE	1 (1)	44.4	GTP cyclohydrolase 1 1	2509.17	3171.8		1.26
CYC5_PSEME	1 (1)	44.17	Cytochrome c5	6877.14	5184.34	1.33	
CAS6_PSEAB	1 (1)	44.1	CRISPR-associated endonuclease Cas6/Csy4	587.99	827.33		1.41
RL32_PSEA7	1 (1)	42.75	50S ribosomal protein L32	6330.02	1420.99	4.45	

TIG_PSEA6	3 (2)	41.73	Trigger factor	2472.36	2232.45	1.11	
NADC_PSEAE	1 (1)	41.27	Nicotinate-nucleotide pyrophosphorylase [carboxylating]	793.29	20.13	39.41	
ARNB_PSEAE	3 (3)	40.8	UDP-4-amino-4-deoxy-L-arabinose--oxoglutarate aminotransferase	6876.78	2084.46	3.30	
RS19_PSE14	1 (1)	39.03	30S ribosomal protein S19	1911.82	456.18	4.19	
HIS81_PSEAE	2 (1)	38.56	Histidinol-phosphate aminotransferase 1	1210.71	14.87	81.42	
ASTE_PSEAB	2 (1)	38.41	Succinylglutamate desuccinylase	0	1063.02		∞
RLUC_PSEAE	1 (1)	37.41	Ribosomal large subunit pseudouridine synthase C	1860.74	1200.18	1.55	
OBG_PSEAB	2 (1)	37.07	GTPase Obg	11.43	2899.72		253.69
PGK_PSEA8	2 (1)	36.95	Phosphoglycerate kinase	40.9	705.77		17.26
FETP_PSEAB	1 (1)	35.88	Probable Fe(2+)-trafficking protein	2763.53	344.38	8.02	
YID1_PSEAE	3 (3)	35.86	Uncharacterized protein PA5481	4845.02	2014.49	2.41	
ACKA_PSEAB	2 (2)	31.32	Acetate kinase	49.76	338.7		6.81

Supplementary Table S5. Cell-free secretome of *P. aeruginosa* CF isolate 7 following growth under normoxia and anoxia. Data represents the average normalised abundances and fold increase from *N*=1. A fold change of 1.0 is indicative of no change between normoxia and anoxia.. I7A = *P. aeruginosa* CF isolate 7 grown under anoxia. I7O = *P. aeruginosa* CF isolate 7 grown under normoxia.

Accession	Peptides (unique)	Score	Description	Average Normalised Abundances		Fold change	
				I7O	I7A	I7O	I7A
FLICA_PSEAI	37 (33)	3589.39	A-type flagellin	4.63E+06	6.96E+06		1.50
IMPA_PSEAE	49 (48)	3562.38	Immunomodulating metalloprotease	6.07E+05	2.73E+06		4.50
ELAS_PSEAE	29 (28)	3120.58	Elastase	4.59E+06	3.21E+06	1.43	
BRAC_PSEAE	25 (23)	2328.65	Leucine-, isoleucine-, valine-, threonine-, and alanine-binding protein	1.66E+06	8.05E+05	2.06	
Y807_PSEAB	26 (25)	2170.15	Putative prophage major tail sheath protein	8.64E+05	3.44E+06		3.98
LAP_PSEAB	25 (25)	1954.53	Aminopeptidase	2.89E+06	4.30E+06		1.49
ATPB_PSEAB	22 (22)	1807.3	ATP synthase subunit beta	2.91E+05	3.82E+05		1.31
NIRS_PSEAE	26 (26)	1725.26	Nitrite reductase	2.54E+05	4.25E+05		1.67

APRA_PSEAE	21 (21)	1721.13	Serralysin	7.21E+05	2.52E+06		3.50
EFG1_PSEAE	30 (22)	1584.83	Elongation factor G 1	3.07E+05	8.89E+04	3.45	
GGT_PSEAE	30 (29)	1578.55	Glutathione hydrolase proenzyme	1.68E+05	2.55E+05		1.52
CATA_PSEAE	27 (26)	1545.15	Catalase	1.77E+05	3.40E+05		1.92
CH60_PSEA7	26 (25)	1433.25	60 kDa chaperonin	2.69E+05	4.43E+05		1.65
Y421_PSEA8	16 (15)	1358.82	UPF0312 protein PLES_04211	3.01E+05	6.05E+04	4.98	
ASPQ_PSEAE	23 (22)	1314.67	Glutaminase-asparaginase	2.75E+05	1.71E+05	1.61	
FUMC2_PSEAE	20 (17)	1281.58	Fumarate hydratase class II 2	3.00E+05	1.10E+05	2.73	
DAVT_PSEAE	16 (16)	1266.37	5-aminovalerate aminotransferase DavT	2.29E+05	2.07E+05	1.11	
ATOB_PSEAE	17 (16)	1261.18	Acetyl-CoA acetyltransferase	2.44E+05	1.46E+05	1.67	
Y3922_PSEAE	19 (19)	1244.88	Uncharacterized protein PA3922	2.23E+05	1.73E+05	1.29	
LYSC_PSEAB	11 (11)	1228.7	Lysyl endopeptidase	7.29E+05	4.97E+05	1.47	
DHE2_PSEAE	24 (22)	1220.4	NAD-specific glutamate dehydrogenase	4.82E+04	8.09E+04		1.68
P5217_PSEAE	17 (14)	1192.1	Probable binding protein component of ABC iron transporter PA5217	2.65E+05	3.16E+04	8.39	
DLDH1_PSEAE	17 (17)	1180.89	Dihydrolipoyl dehydrogenase	1.12E+05	8.04E+04	1.39	
MASZ_PSEA7	22 (16)	1150.9	Malate synthase G	5.47E+04	2.90E+04	1.89	
PILY1_PSEAW	15 (14)	1109.35	Type IV pilus biogenesis factor PilY1	4.14E+04	6.77E+04		1.64
ATPA_PSEAB	19 (18)	1106.82	ATP synthase subunit alpha	2.42E+05	1.89E+05	1.28	
CBPD_PSEAE	14 (3)	1100.26	Chitin-binding protein CbpD	1222.8	6318.03		5.17
FABY_PSEAE	16 (16)	1099.54	Beta-ketoacyl-[acyl-carrier-protein] synthase FabY	9.96E+04	4.92E+04	2.02	
PORF_PSEAE	15 (15)	1089.51	Outer membrane porin F	2.10E+05	8.96E+04	2.34	
AMPA_PSEAB	15 (8)	1061.24	Probable cytosol aminopeptidase	3.38E+04	5.04E+04		1.49
PNP_PSEAB	16 (16)	1022.98	Polyribonucleotide nucleotidyltransferase	1.39E+05	9.93E+04	1.40	
MASZ_PSEAB	18 (13)	994.58	Malate synthase G	5.21E+04	2.06E+04	2.53	
NOSZ_PSEAE	21 (17)	976.55	Nitrous-oxide reductase	5.89E+04	1.28E+05		2.17
SAHH_PSEAB	16 (14)	904.5	Adenosylhomocysteinase	9.99E+04	1.62E+05		1.62
CBPD_PSEAB	12 (9)	898.73	Chitin-binding protein CbpD	7.71E+04	1.41E+05		1.83
OTCC_PSEAE	19 (19)	892.45	Ornithine carbamoyltransferase, catabolic	2.35E+05	1.30E+05	1.81	
PGK_PSEA8	14 (14)	858.35	Phosphoglycerate kinase	1.95E+05	9.81E+04	1.99	

FLID1_PSEAI	14 (13)	850.79	A-type flagellar hook-associated protein 2	4.52E+04	2.49E+04	1.82	
DSBA_PSEAB	14 (13)	812.31	Thiol:disulfide interchange protein DsbA	2.29E+05	4.06E+04	5.64	
SPUD_PSEAB	17 (16)	807.21	Putrescine-binding periplasmic protein SpuD	4.03E+05	1.07E+05	3.77	
DAPA_PSEAE	11 (11)	776.34	4-hydroxy-tetrahydrodipicolinate synthase	8.46E+04	4.10E+04	2.06	
HUTU_PSEAB	16 (15)	761.29	Urocanate hydratase	5.67E+04	5.69E+04		1.00
GSHR_PSEAE	10 (9)	742.12	Glutathione reductase	5.46E+04	6.58E+04		1.21
DAVD_PSEAE	13 (10)	739.95	Glutarate-semialdehyde dehydrogenase DavD	6.78E+04	5.64E+04	1.20	
CISY_PSEAE	17 (17)	733.52	Citrate synthase	9.88E+04	9.84E+04	1.00	
IDH_PSEAB	11 (11)	725.1	Isocitrate dehydrogenase [NADP]	6461.41	7.71E+04		11.93
SYI_PSEAE	13 (3)	709.6	Isoleucine--tRNA ligase	3140.55	2067.53	1.52	
RPOC_PSEAB	16 (14)	709.47	DNA-directed RNA polymerase subunit beta'	5332.75	1.87E+04		3.51
SYI_PSEA7	20 (9)	669.64	Isoleucine--tRNA ligase	6.48E+04	4.09E+04	1.58	
ARUC_PSEAE	12 (10)	660.95	Succinylornithine transaminase/acetylornithine aminotransferase	1.10E+05	2.94E+04	3.74	
TOLB_PSEAE	10 (9)	643.23	Protein TolB	2.13E+04	3.08E+04		1.45
EFG2_PSEAE	10 (4)	624.24	Elongation factor G 2	1.70E+04	1.67E+04	1.02	
THIO_PSEAE	9 (8)	622	Thioredoxin	6.31E+04	8907.59	7.08	
SYI_PSEAB	11 (3)	619.52	Isoleucine--tRNA ligase	3.49E+04	1.66E+04	2.10	
ODP2_PSEAE	10 (10)	617.39	Dihydrolipoyllysine-residue acetyltransferase component of pyruvate dehydrogenase complex	1.83E+04	2.83E+04		1.55
GPMI_PSEAE	11 (8)	608.47	2,3-bisphosphoglycerate-independent phosphoglycerate mutase	7.32E+04	2.84E+04	2.58	
AAT_PSEAE	10 (9)	596.02	Aspartate aminotransferase	5564.44	2.82E+04		5.07
Y1579_PSEAE	13 (12)	593.12	Uncharacterized protein PA1579	6.36E+04	7269.84	8.75	
PCKA_PSEAE	14 (11)	574.99	Phosphoenolpyruvate carboxykinase (ATP)	3.30E+04	1.95E+04	1.69	
GPMI_PSEAB	8 (2)	540.63	2,3-bisphosphoglycerate-independent phosphoglycerate mutase	7290.43	8764.32		1.20
PROA_PSEAB	10 (1)	536.13	Gamma-glutamyl phosphate reductase	6987.28	4299.79	1.63	
GCSP1_PSEAE	15 (11)	533.16	Glycine dehydrogenase (decarboxylating) 1	3.76E+05	1.20E+05	3.13	
ILVC_PSEAB	10 (9)	526.34	Ketol-acid reductoisomerase (NADP(+))	4.03E+04	2.30E+04	1.75	

CYSNC_PSEAE	9 (9)	523.06	Bifunctional enzyme CysN/CysC	3.26E+04	1.49E+04	2.19	
FLICB_PSEAE	7 (2)	513.41	B-type flagellin	3.90E+04	7480.17	5.21	
METK_PSEAB	10 (9)	495.62	S-adenosylmethionine synthase	6.79E+04	1.71E+04	3.97	
SYV_PSEAE	13 (11)	495.56	Valine--tRNA ligase	2.63E+04	1.42E+04	1.85	
PURE_PSEAE	5 (5)	495.13	N5-carboxyaminoimidazole ribonucleotide mutase	3.12E+04	3624.23	8.61	
PROA_PSEA7	10 (2)	488.5	Gamma-glutamyl phosphate reductase	3208.65	4325.88		1.35
SYR_PSEAB	11 (11)	463.78	Arginine--tRNA ligase	9412.27	8.65E+04		9.19
PORD_PSEAE	8 (8)	457.18	Porin D	6.67E+04	4.41E+04	1.51	
FABB_PSEAB	7 (6)	452.02	3-oxoacyl-[acyl-carrier-protein] synthase 1	6.89E+04	2.73E+04	2.52	
SPUE_PSEAE	10 (8)	446.16	Spermidine-binding periplasmic protein SpuE	4.93E+04	2.55E+04	-1.93	
HEM2_PSEAE	5 (5)	427.89	Delta-aminolevulinic acid dehydratase	1.97E+04	2.24E+04		1.14
PQSB_PSEAE	7 (7)	427.79	2-heptyl-4(1H)-quinolone synthase subunit PqsB	3.23E+04	3439.98	9.39	
IPYR_PSEAE	8 (8)	420.41	Inorganic pyrophosphatase	2.94E+04	3062.3	9.60	
AHPC_PSEAB	7 (6)	410.3	Alkyl hydroperoxide reductase C	1.02E+05	2.69E+04	3.79	
DNAK_PSEAB	8 (8)	408.35	Chaperone protein DnaK	4213.8	1.74E+04		4.13
GATA_PSEAE	6 (2)	404.01	Glutamyl-tRNA(Gln) amidotransferase subunit A	5954.4	4121.81	1.44	
DCTP_PSEAE	7 (6)	399.98	C4-dicarboxylate-binding periplasmic protein DctP	3.85E+04	1.17E+04	3.29	
NIRF_PSEAE	5 (5)	399.93	Protein NirF	3061.88	1.69E+04		5.52
PYRC_PSEAE	6 (6)	397.51	Dihydroorotase	1.76E+04	6411.51	2.75	
LIP_PSEAE	6 (5)	396.13	Triacylglycerol lipase	1.46E+04	2.07E+04		1.42
SODM_PSEAE	8 (7)	395.32	Superoxide dismutase [Mn]	1.32E+05	1.49E+04	8.86	
DHAS_PSEAE	7 (7)	387.06	Aspartate-semialdehyde dehydrogenase	3.45E+04	2.61E+04	1.32	
ACNB_PSEAE	7 (7)	386.3	Aconitate hydratase B	8600.11	4.86E+04		5.65
SODF_PSEAE	4 (4)	383.5	Superoxide dismutase [Fe]	4.51E+05	2.93E+05	1.54	
FADB_PSEAB	8 (8)	381.96	Fatty acid oxidation complex subunit alpha	1177.03	9861.66		8.38
GATB_PSEAB	7 (4)	364.53	Aspartyl/glutamyl-tRNA(Asn/Gln) amidotransferase subunit B	1.52E+04	2208.17	6.88	
LASA_PSEAB	7 (7)	354.17	Protease LasAe	1.24E+05	9.37E+04	1.32	
ETFA_PSEAE	6 (6)	353.4	Electron transfer flavoprotein subunit alpha	2.80E+04	3628.91	7.72	
ACKA_PSEAB	8 (3)	353.31	Acetate kinase	2061.32	4400.38		2.13

NLPD_PSEAE	5 (5)	352.7	Lipoprotein NlpD/LppB homolog	3899.47	9122.23		2.34
HEM6_PSEA8	7 (7)	347.43	Oxygen-dependent coproporphyrinogen-III oxidase	1.97E+04	2.78E+04		1.41
ETFB_PSEAE	7 (6)	347.05	Electron transfer flavoprotein subunit beta	2.73E+04	5334.8	5.12	
SPUC_PSEAE	6 (6)	335.12	Putrescine--pyruvate aminotransferase	9135.18	7493.05	1.22	
ACKA_PSEA7	7 (2)	332.9	Acetate kinase	1.23E+04	3.42E+04		2.78
DPO3B_PSEAE	5 (5)	319.48	Beta sliding clamp	1.95E+04	8751.74	2.23	
MMSA_PSEAE	5 (5)	308.96	Methylmalonate-semialdehyde dehydrogenase [acylating]	1.40E+04	2.86E+04		2.04
EDD_PSEAE	6 (6)	290.45	Phosphogluconate dehydratase	2.11E+04	2.74E+04		1.30
AGUA_PSEAE	4 (2)	280.84	Agmatine deiminase	3324.84	4340.32		1.31
GLN1B_PSEAE	7 (7)	264.25	Glutamine synthetase	2.27E+04	2.58E+04		1.14
PILY1_PSEAE	7 (7)	257.75	Type IV pilus biogenesis factor PilY1	2.79E+04	3.20E+04		1.15
PH4H_PSEAE	4 (4)	257.36	Phenylalanine-4-hydroxylase	2.15E+04	2.32E+04		1.08
GATA_PSEAB	4 (1)	255.27	Glutamyl-tRNA(Gln) amidotransferase subunit A	1197.02	950.76	1.26	
ARGC_PSEAB	4 (3)	253.56	N-acetyl-gamma-glutamyl-phosphate reductase	6176.89	5874.67	1.05	
ILVE_PSEAE	4 (4)	243.49	Branched-chain-amino-acid aminotransferase	3.52E+04	1.76E+04	2.00	
FPTA_PSEAE	4 (4)	242.95	Fe(3+)-pyochelin receptor	8866.12	1.10E+04		1.24
FUMC1_PSEAE	5 (5)	239.27	Fumarate hydratase class II 1	1.74E+04	1.54E+04	1.13	
LOLA_PSEAB	4 (3)	238.48	Outer-membrane lipoprotein carrier protein	8102.86	2745.99	2.95	
FADA_PSEAB	4 (4)	237.78	3-ketoacyl-CoA thiolase	842	6639.63		7.89
SYE_PSEA7	4 (4)	234.54	Glutamate--tRNA ligase	7665.45	1679.85	4.56	
TOP1_PSEAE	7 (5)	234.21	DNA topoisomerase 1	4.33E+04	1.72E+04	2.52	
CHMU_PSEAE	4 (4)	232.63	Monofunctional chorismate mutase	8706.94	7995.06	1.09	
SSB_PSEAE	4 (3)	232.17	Single-stranded DNA-binding protein	1.27E+04	2250.29	5.64	
AK_PSEAE	8 (8)	232.15	Aspartokinase	1.96E+04	5192.46	3.77	
LEU3_PSEAE	3 (3)	227.62	3-isopropylmalate dehydrogenase	2714.73	7035.58		2.59
PAGL_PSEAE	3 (3)	226.88	Lipid A deacylase PagL	6511.24	3596.35	1.81	
GSHB_PSEAE	4 (4)	225.42	Glutathione synthetase	7657.24	6003.19	1.28	
SYL_PSEAB	5 (5)	223.06	Leucine--tRNA ligase	1.70E+04	2576.54	6.60	
GYRA_PSEAE	5 (4)	222.85	DNA gyrase subunit A	7084.7	5449.5	1.30	

DEF_PSEAE	3 (3)	210.39	Peptide deformylase	1.09E+05	1.16E+05		1.06
FABI_PSEAE	3 (2)	208.14	Enoyl-[acyl-carrier-protein] reductase [NADH] FabI	1626.69	311.25	5.23	
PAL_PSEAE	4 (4)	205.71	Peptidoglycan-associated lipoprotein	1.08E+04	3211.83	3.36	
GLMU_PSEAE	5 (4)	198.94	Bifunctional protein GlmU	1.46E+04	1103.31	13.23	
SUHB_PSEAE	4 (4)	197.98	Inositol-1-monophosphatase	1.05E+04	3061.8	3.43	
ALKD_PSEAE	3 (3)	195.98	2-dehydro-3-deoxy-phosphogluconate aldolase	5541.55	1775.8	3.12	
ARGC_PSEAE	3 (3)	194.73	N-acetyl-gamma-glutamyl-phosphate reductase	2313.97	2770.43		1.20
HEM3_PSEA8	4 (4)	192.9	Porphobilinogen deaminase	1364.2	1874.19		1.37
RNPH_PSEAB	2 (2)	191.57	Ribonuclease PH	6358.46	693.89	9.16	
BAUA_PSEAE	5 (4)	190.16	Beta-alanine--pyruvate aminotransferase	8043.6	1.52E+04		1.89
BFR_PSEAE	4 (3)	186.41	Bacterioferritin	6536.28	6499.07	1.01	
AZUR_PSEAE	4 (3)	185.92	Azurin	2.96E+05	7.51E+04	3.94	
PCKA_PSEA7	7 (6)	181.54	Phosphoenolpyruvate carboxykinase (ATP)	8356.54	9572.2		1.15
ISCS_PSEAB	4 (3)	173.5	Cysteine desulfurase IscS	1.08E+04	7.56E+04		7.00
STHA_PSEAB	3 (3)	171.53	Soluble pyridine nucleotide transhydrogenase	3260.97	4715.82		1.45
AHPF_PSEAE	6 (5)	170.53	Alkyl hydroperoxide reductase subunit F	9307.1	856.16	10.87	
RRAAH_PSEA7	4 (3)	160.88	Putative 4-hydroxy-4-methyl-2-oxoglutarate aldolase	6.53E+04	7.18E+04		1.10
CATE_PSEAE	3 (2)	159.59	Catalase HPII	947.59	2818.09		2.97
NAPA_PSEAB	5 (5)	158.75	Periplasmic nitrate reductase	3810.71	4172.73		1.10
LDC_PSEAE	2 (2)	155.3	Murein tetrapeptide carboxypeptidase	4678.6	1529.34	3.06	
PPIA_PSEAE	3 (3)	154.38	Peptidyl-prolyl cis-trans isomerase A	7697.21	1939.43	3.97	
PDXJ_PSEAB	3 (3)	152.79	Pyridoxine 5'-phosphate synthase	1.56E+04	3644.7	4.28	
ESTA_PSEAE	2 (2)	148.08	Esterase EstA	3507.64	2461.11	1.43	
FABZ_PSEAB	4 (4)	146.05	3-hydroxyacyl-[acyl-carrier-protein] dehydratase FabZ	1.05E+04	1100.25	9.54	
PURK_PSEAE	3 (3)	142.88	N5-carboxyaminoimidazole ribonucleotide synthase	1.36E+04	1.28E+04	1.06	
FUR_PSEAE	2 (2)	141.92	Ferric uptake regulation protein	5959.49	384.45	15.50	
PHS_PSEAB	3 (3)	139.29	Putative pterin-4-alpha-carbinolamine dehydratase	5554.42	1113.98	4.99	
DAPD_PSEAE	2 (1)	137.02	2,3,4,5-tetrahydropyridine-2,6-dicarboxylate N-succinyltransferase	4050.49	2521.48	1.61	

LGUL_PSEAE	4 (3)	135.46	Lactoylglutathione lyase	2625.19	377.86	6.95	
LEU1_PSEA7	3 (3)	130.19	2-isopropylmalate synthase	2734.51	9217.13		3.37
BCCP_PSEAE	2 (2)	128.68	Biotin carboxyl carrier protein of acetyl-CoA carboxylase	1.48E+04	1382.25	10.71	
PYRB_PSEAB	2 (2)	127.56	Aspartate carbamoyltransferase	1776.2	1790.56		1.01
BAMB_PSEAE	3 (2)	126.01	Outer membrane protein assembly factor BamB	372.5	1907.89		5.12
GMHA_PSEAB	3 (3)	124.92	Phosphoheptose isomerase	9712.96	1077.8	9.01	
SYT_PSEA7	4 (4)	124.46	Threonine--tRNA ligase	4836.97	1860.35	2.60	
GATB_PSEA7	4 (2)	123.06	Aspartyl/glutamyl-tRNA(Asn/Gln) amidotransferase subunit B	5170.5	838.27	6.17	
HFQ_PSEAB	3 (3)	122.28	RNA-binding protein Hfq	6416.85	3111.37	2.06	
ILVD_PSEAB	3 (3)	119.3	Dihydroxy-acid dehydratase	804.5	722.81	1.11	
NQRF_PSEAE	2 (2)	116.24	Na(+)-translocating NADH-quinone reductase subunit F	3248	714.97	4.54	
GCH12_PSEAE	4 (4)	115.66	GTP cyclohydrolase 1 2	4425.36	1249.16	3.54	
RLPA_PSEAB	3 (3)	115.15	Endolytic peptidoglycan transglycosylase RlpA	1446.96	2795.51		1.93
SECB_PSEAB	2 (2)	115.06	Protein-export protein SecB	3199.21	418.59	7.64	
Y3753_PSEAE	2 (2)	114.84	Uncharacterized protein PA3753	7499.59	3650.27	2.05	
TAL_PSEA7	3 (1)	114.66	Transaldolase	1647.57	3519.79		2.14
MURD_PSEAE	2 (2)	112.38	UDP-N-acetylmuramoylalanine--D-glutamate ligase	4434.5	2332.82	1.90	
PYRX_PSEAE	5 (4)	110.76	Dihydroorotase-like protein	7024.61	6361.23	1.10	
RISB_PSEAB	3 (2)	110.74	6,7-dimethyl-8-ribityllumazine synthase	3579.49	2701.37	1.33	
FMNRE_PSEAE	3 (3)	110.19	NAD(P)H-dependent FMN reductase PA1204	8469.06	3805.84	2.23	
NDK_PSEAB	5 (5)	108.38	Nucleoside diphosphate kinase	1.02E+04	1885.3	5.41	
CLPB_PSEAE	3 (3)	107.07	Chaperone protein ClpB	4561.05	1524.99	2.99	
TAL_PSEAB	2 (1)	106.51	Transaldolase O	227.24	227.36		1.00
TOLQ_PSEAE	1 (1)	101.97	Protein TolQ	297.22	390.52		1.31
MTIP_PSEAE	2 (2)	100.73	S-methyl-5'-thioinosine phosphorylase	1778.69	1051.62	1.69	
AROE_PSEAE	2 (2)	98.97	Shikimate dehydrogenase (NADP(+))	796.08	2001.48		2.51
NUOCD_PSEAB	3 (2)	97.78	NADH-quinone oxidoreductase subunit C/D	1.83E+04	1.98E+04		1.08
METZ_PSEAE	2 (1)	97.36	O-succinylhomoserine sulfhydrylase	90.49	794.09		8.78
COBN_PSEAE	3 (3)	96.92	Aerobic cobaltochelatase subunit CobN	317.72	6266.77		19.72
PSTS_PSEAB	3 (2)	94.55	Phosphate-binding protein PstS	5695.89	802.38	7.10	

ARCA_PSEAE	2 (2)	94.3	Arginine deiminase	494.85	2781.39		5.62
GCH1L_PSEAE	2 (2)	94.23	GTP cyclohydrolase 1 type 2 homolog	1555.48	329.59	4.72	
HISX_PSEAE	3 (2)	93.61	Histidinol dehydrogenase	788.82	2889.26		3.66
HTPX_PSEAB	1 (1)	93.37	Protease HtpX	89.02	181.45		2.04
BEKAS_PSEAE	3 (2)	91.38	Beta-ketodecanoyl-[acyl-carrier-protein] synthase	1217.02	4891.8		4.02
QOR_PSEAE	2 (2)	87.85	Quinone oxidoreductase O	578.68	507.47	1.14	
CYSM_PSEAE	3 (2)	86.17	Cysteine synthase B	1.28E+04	7889.75	1.62	
NORB_PSEAE	3 (1)	84.66	Nitric oxide reductase subunit B	172.97	2598.4		15.02
RPIA_PSEAB	2 (2)	84.01	Ribose-5-phosphate isomerase A	157.67	85.06	1.85	
AMIE_PSEA8	2 (2)	83.17	Aliphatic amidase	857.02	1871.26		2.18
ASTB_PSEAB	3 (3)	81.61	N-succinylarginine dihydrolase	1.03E+04	5476.26	1.88	
GLO2_PSEA7	2 (1)	79.54	Hydroxyacylglutathione hydrolase	571.74	284.15	2.01	
ACP1_PSEAE	2 (2)	76.32	Acyl carrier protein 1	7527.94	1435.74	5.24	
HISZ_PSEAB	1 (1)	75.84	ATP phosphoribosyltransferase regulatory subunit	1069.59	710.69	1.51	
FMK1_PSEAI	1 (1)	75.54	Fimbrial protein	3524.2	1141.47	3.09	
TYSY_PSEAB	2 (2)	74.88	Thymidylate synthase	7102.59	7641.33		1.08
Y2116_PSEAE	2 (2)	74.72	Putative hydro-lyase PA2116	4248.96	4127.6	1.03	
OPRM_PSEAE	1 (1)	70.94	Outer membrane protein OprM	296.31	1737.28		5.86
PQSC_PSEAE	3 (3)	69.54	2-heptyl-4(1H)-quinolone synthase subunit PqsC	3988.88	1.81E+04		4.54
Y2770_PSEAE	2 (1)	68.95	Uncharacterized isomerase PA2770	1453.01	447.82	3.24	
PDXA_PSEAE	1 (1)	68.71	4-hydroxythreonine-4-phosphate dehydrogenase	586.3	696.92		1.19
RPOB_PSEAB	4 (3)	66.99	DNA-directed RNA polymerase subunit beta	417.42	6529.77		15.64
CYNS_PSEAB	2 (2)	66.47	Cyanate hydratase	7814.67	7726.41	1.01	
MTNC_PSEAB	1 (1)	65.56	Enolase-phosphatase E1	361.13	0.61	592.02	
PDXY_PSEAB	1 (1)	64.89	Pyridoxal kinase PdxY	520.75	274.28	1.90	
HIS7_PSEA7	1 (1)	63.69	Imidazoleglycerol-phosphate dehydratase	792.19	496.45	1.60	
GCSH1_PSEAE	2 (2)	63.28	Glycine cleavage system H protein 1	7136.94	1373.74	5.20	
G6PI_PSEA7	2 (2)	56.35	Glucose-6-phosphate isomerase	1652.8	1305.12	1.27	
Y049_PSEAE	1 (1)	55.94	Uncharacterized protein PA0049	18.08	172.96		9.57

RMLC_PSEAE	1 (1)	54.26	dTDP-4-dehydrorhamnose 3,5-epimerase	779.69	329.53	2.37	
CPDA_PSEAI	1 (1)	54.04	3',5'-cyclic adenosine monophosphate phosphodiesterase CpdA	358.29	156.76	2.29	
ASTE_PSEAB	1 (1)	52.48	Succinylglutamate desuccinylase	581.57	346.64	1.68	
PIUC_PSEAE	1 (1)	51.47	PKHD-type hydroxylase PiuC	1697.81	88.07	19.28	
GCSH2_PSEAE	1 (1)	49.67	Glycine cleavage system H protein 2	1582.76	200.03	7.91	
AROB_PSEA7	2 (1)	48.44	3-dehydroquinate synthase	1982.18	1886.25	1.05	
TOXA_PSEAE	1 (1)	47.72	Exotoxin A	375.19	1283.95		3.42
OTCA_PSEAE	3 (3)	45.64	Ornithine carbamoyltransferase, anabolic	6.72E+04	3.37E+04	1.99	
LPTD_PSEA8	1 (1)	45.08	LPS-assembly protein LptD	481.84	1030.82		2.14
DAPB_PSEAB	1 (1)	43.16	4-hydroxy-tetrahydrodipicolinate reductase	2019.79	636.26	3.17	
APEB_PSEAB	2 (2)	37.64	Probable M18 family aminopeptidase 2	569.57	1212.15		2.13
HUTH_PSEAB	1 (1)	35.44	Histidine ammonia-lyase	599.18	981.35		1.64
NUOF_PSEAE	2 (2)	35.33	NADH-quinone oxidoreductase subunit F	2934.76	5397.42		1.84
KYNB_PSEAB	1 (1)	34.07	Kynurenine formamidase	2045.23	39.14	52.25	
THRH_PSEAE	2 (2)	32.42	Phosphoserine phosphatase ThrH	2.64E+04	5648.48	4.67	
